# Isolation and Characterization of Terpenoids with Promising Biopesticide Activity from *Dittrichia viscosa* (L.) Roots

**DOI:** 10.3390/ijms27072949

**Published:** 2026-03-24

**Authors:** María José Segura-Navarro, José Francisco Quílez del Moral, Alberto Galisteo, José Luis López-Pérez, Diego O. Molina Inzunza, María Fe Andrés, Azucena González-Coloma, Alejandro Fernández Barrero

**Affiliations:** 1 Departamento de Química Orgánica, Instituto de Biotecnología, Universidad de Granada, 18071 Granada, Spain; mariajseguranavarro@gmail.com (M.J.S.-N.); albertogapre@ugr.es (A.G.); diegomolina96@gmail.com (D.O.M.I.); 2 Departamento de Química Farmacéutica, Centro de Enfermedades Tropicales, Instituto de Investigación Biomédica de Salamanca, Universidad de Salamanca, 37007 Salamanca, Spain; lopez@usal.es; 3 Departamento de Farmacología, Facultad de Medicina, Centro de Investigaciones Psicofarmacológicas, Universidad de Panamá, Panama City 3366, Panama; 4 Instituto de Ciencias Agrarias, Centro Superior de Investigaciones Científicas, 28006 Madrid, Spain; mafay@ica.csic.es (M.F.A.); azu@ica.csic.es (A.G.-C.)

**Keywords:** *Dittrichia viscosa* roots, terpenes, sesquiterpene lactones, himachalanes, biopesticides

## Abstract

The natural product composition of the hexane and methyl *tert*-butyl ether extracts of *Dittrichia viscosa* roots was examined. Eight terpenoids were identified by nuclear magnetic resonance (NMR) and high resolution mass spectroscometry (HRMS) techniques, four of which (**1**, **5**, **6** and **8**) are reported here for the first time as natural products. Of these eight compounds, four are thymol derivatives (**1**–**4**), two are guaianolides (**5** and **7**) and two are himachalanes (**6** and **8**). Additionally, the occurrence of himachalanes in this species is reported for the first time. Furthermore, a study of the potential plant protection effects of some of these natural products and the chemical derivative **6a** was carried out. Promising preliminary results were obtained for compounds **1**–**3** and **6a** as antifeedant agents against *Spodoptera littoralis*; **1**–**3** and **5** against *Myzus persicae*; **1**–**3** against *Rhopalosiphum padi*; and **4** as nematicide against *Meloidogyne javanica*. Finally, the phytotoxic activity of compounds **4**, **5** and **6a** against the monocotyledonous species *Lolium perenne* was also proven.

## 1. Introduction

The exponential growth of the world’s population in recent decades makes it necessary to maximize agricultural production yields. In this context, the demand for pesticides that ensure a good harvest is continuously increasing; however, chemical pesticides can also have adverse effects on the environment and human health [1,2,3]. In this regard, biopesticides are natural products produced by organisms like bacteria, viruses, fungi or plants that are generally regarded as safe, efficient, highly targeted and biodegradable agents [4]. As a result of the gradual discontinuation of numerous chemical pesticides in Europe, the ongoing assessment of these chemicals, and the increasing demand for organic food, the production of biopesticides has witnessed a substantial rise in recent years [5]. Indeed, in the framework of the European Green Pact and, in particular, its “Farm to Fork” strategy, the European Commission plans to reduce the use of chemical pesticides by 50% by 2030 (https://www.europarl.europa.eu/factsheets/en/sheet/78/los-productos-quimicos-ylosplaguicidas (accessed on 18 March 2026).

Within this specific context, our research group is dedicated to the investigation of novel biopesticides from plants. For this study, we selected *Dittrichia viscosa* (L.) Greuter, a woody plant with yellow flowers belonging to the Asteraceae family, distributed throughout the Mediterranean area and commonly found in abandoned fields, roadsides, and disturbed areas, due to its excellent colonizing ability [6].

*D. viscosa* has been used in traditional medicine due to its anti-inflammatory, antipyretic, antiseptic, sedative, antispasmodic, antidiarrheic, antimicrobial, and anthelmintic properties [7,8]. More recently, its insecticidal activity against pests such as the wheat weevil (*Sitophilus granaries*) has been confirmed [9], as well as its allelopathic [10] and antidepressant potential [11]. Furthermore, *D. viscosa* provides ecosystem functions [12]. For example, *D. viscosa* is a host plant for the aphid predator *Macrolophus melanotoma* in agroecosystems [13].

Encouraged by this range of reported bioactivities and potential applications, several studies of the chemical composition of the aerial parts of this plant have been carried out, revealing the presence of dozens of natural compounds [14,15,16]. However, little is known about the composition of the roots, which have not been studied in detail, and only a few compounds such as chlorogenic acid, dicaffeoyl quinic acid, several germacranolides, coumaric acid and *p*-cymene derivatives **2** and **3** have been reported [17,18,19,20].

In this article, we present the results of an initial study on the chemical composition of the root constituents of *D. viscosa*, which led to the identification of four new natural products (**1**, **5**, **6**, and **8**). We also evaluated the biopesticidal activity of these compounds against several insect pests (*Rhopalosiphum padi*, *Myzus persicae*, *Spodoptera littoralis*) and against an economically important phytoparasitic nematode (*Meloidogyne javanica*). Furthermore, their herbicidal activity was assessed on both a monocotyledonous species (ryegrass, *Lolium perenne*) and a dicotyledonous species (lettuce, *Lactuca sativa*).

The target species were selected based on their economic importance and their availability in the laboratory. The insects *S. littoralis* and *M. persicae* feed on a wide range of horticultural crops [21,22]. *R. padi* is a major cereal pest [23]. The root-knot nematode *M. javanica* is among the most destructive among nematodes worldwide due to the formation of root galls in the host [24,25]. Plant-parasitic nematodes are the most devastating group of plant pathogens worldwide, and their control is extremely challenging. Thus, in the last decade, much effort has been focused on the study of natural nematicidal agents for the management of root-knot nematodes, *Meloidogyne* spp., one of the most economically damaging genera on horticultural and field crops [26]. Perennial ryegrass (*Lolium perenne*) is an important cultivated grass species that becomes a very competitive weed when growing in cultivated crops (https://www.cropscience.bayer.co.nz/pests/weeds/ryegrass---perennial (accessed on 18 March 2026) and is therefore a good weed model to detect herbicidal effects. *Lactuca satica* (lettuce) is used to compare the selectivity of the phytotoxic effects between the monocotyledoneous ryegrass and a horticultural dicotyledoneous crop (lettuce).

## 2. Results

### 2.1. Extraction of D. viscosa Roots

**Figure 1:**
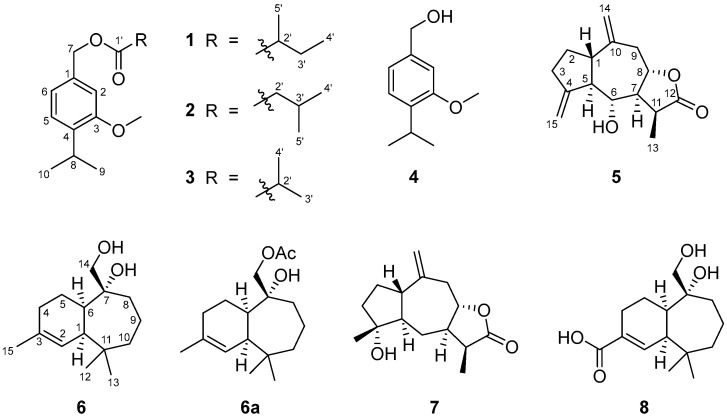
Compounds **1**–**8** isolated from *D. viscosa* roots. Derivative **6a** was obtained from natural diol **6** after acetylation.

Powdered roots of *D. viscosa* were successively extracted using a Soxhlet with hexane (H) and methyl *tert*-butyl ether (MTBE). Each extract was fractionated, and the presence of compounds **1**–**8** could be established (compound **6a** was obtained by acetylation of natural diol **6**) (Figure 1).

Compounds **1**, **5**, **6**, and **8** were found to be new natural products, whereas substances **2**, **3**, **4** and **7** were identified by comparing their spectroscopical data with those reported in the literature [20,27,28] (see NMR Figures S1–S43 in the Supporting Information). No biological activity has been previously reported for compounds **2**, **3** and **4**; however, sesquiterpene lactone **7** has been proven to possess anti-inflammatory and cytotoxic activity [29,30]. The spectroscopic data of compounds **5**, **6** and **8** is shown in Table 1 and Table 2.

### 2.2. Structural Elucidation of New Natural Products

Compounds **1**–**3** were difficult to separate due to their high similarity, so they had to be subjected to several high-performance liquid chromatography (HPLC) analyses to obtain enough of each product to achieve their identification (see Section 4).

**Figure 2:**
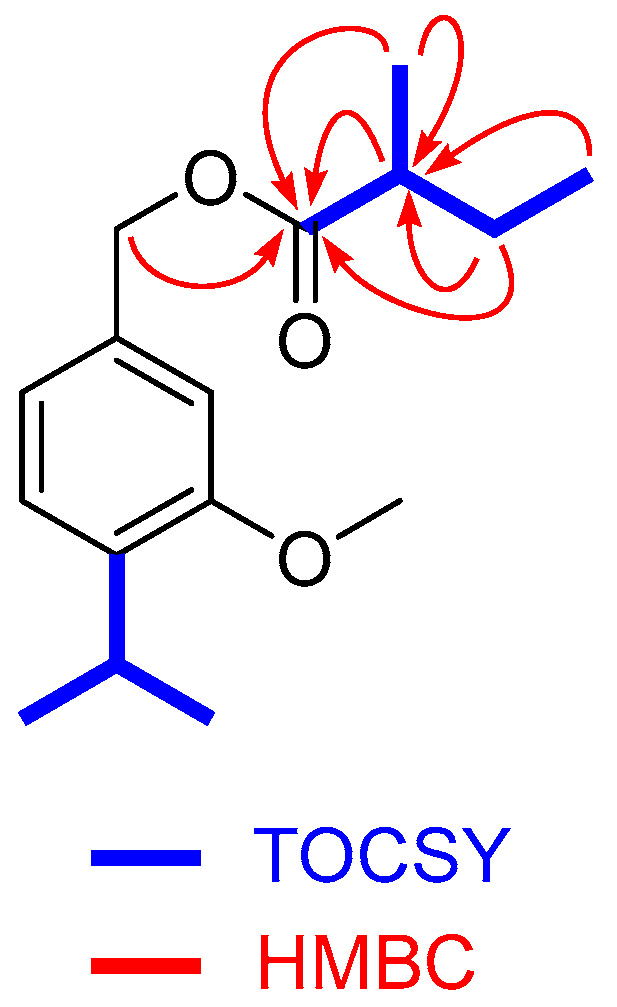
Key TOCSY and HMBC correlations for compound **1**.

Analysis of the NMR data of natural product **1** (Figures S1–S5 in the Supporting Information) confirmed this substance was an ester derivative of 3-methoxy-4-isopropylbenzylalcohol by comparison with data of those previously described **2**–**4** [20,27]. The esterifying moiety on the primary alcohol was determined to be a *sec*-butyl group, as inferred from the corresponding 1D total correlation spectroscopy (TOCSY) experiment after irradiating the methyl signal at δ 0.933 ppm ^1^H (see Figure S4 in the Supporting Information). Additionally, the heteronuclear multiple bond correlations (HMBC) shown in Figure 2 confirmed the location of the *sec*-butyl ester moiety.

**Figure 3:**
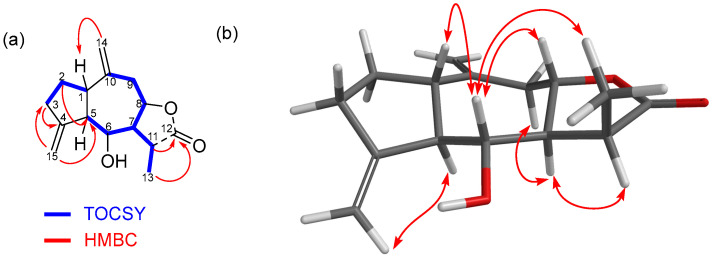
(**a**) Key TOCSY and HMBC correlations for compound **5**. (**b**) Selected 1D NOE correlations.

The molecular formula of compound **5** was C_15_H_20_O_3_, as deduced from the HRMS [M + H]^+^ ion at *m*/*z* 249.1491. This datum, together with the analysis of its ^1^H and ^13^C NMR spectra (Table 1 and Table 2), evidenced that compound **5** was a tricyclic molecule with three unsaturations. The fact that two of these unsaturations were due to the presence of two di-substituted double bonds (δ 4.99; 5.10 and 4.93; 4.91 ppm ^1^H), together with the appearance of a carbonyl group at δ 179.14 ppm ^13^C (attributable to a saturated five-membered lactone), suggested that **5** was a sesquiterpene lactone, most likely a guaianolide [31,32]. Besides the lactone carbonyl group and the di-substituted olefin, the existence of two spin systems (Figure 3a) in compound **5** was deduced after the combined analysis of the ^1^H, ^13^C, heteronuclear single quantum coherence (HSQC), and 1D TOCSY NMR spectra (see Supporting Information, Figures S14–S16, S18 and S19). Finally, the complete planar structure was deduced by observing the correlations between the aforementioned partial structures in the HMBC spectrum (see Figure S17 at Supporting Information). The location of the lactone ring was confirmed after the HMBC correlations from H11 and H13 to C12. Other key HMBC correlations were those observed between H14 and C1, H2 and C5, H15 and C5, and H15 and C3 (Figure 3a).

Finally, the relative configuration of the chiral centers of the molecule was determined based on the analysis of 1D NOE experiments (Figures S20 and S21 in the Supporting Information). We previously computationally calculated the most stable conformation of **5**, as detailed in the Supporting Information (Table S2). The observed nuclear overhauser effect (NOE) correlations from H6 to H1, H8 and H13 suggested the same orientation of these hydrogens, while the correlations from H11 to H7 and H5 to H15 suggested a different orientation (Figure 3b). These data allowed us to propose the relative configuration for compound **5** as *rel*-1*S*, 5*R*, 6*R*, 7*S*, 8*S*, 11*S* (https://doi.org/10.1351/goldbook.R05260).

The computationally calculated ^13^C NMR spectrum of **5**, following the methodology outlined in Section 4, is in complete agreement with the experimental data (rms = 1.18; Max abs. = 2.29), thereby confirming the structure of this compound (. Furthermore, the absolute configuration of compound **5** was determined by calculating the theoretical optical rotation [α]_D_ values using the Gaussian 16 software (see Section 4). The specific rotation value obtained by the methodology described in Section 4 was −91.93 at a wavelength of 589 nm, which shares the sign of the experimental value ([α]_D_ = −40.12). Therefore, the absolute stereochemistry of this compound is established as 1*S*, 5*R*, 6*R*, 7*S*, 8*S*, 11*S*. To the best of our knowledge, this represents the first report of the absolute configuration of a guaianolide isolated from *D. viscosa*.

The molecular formula of **6** was C_15_H_26_O_2_, as deduced from the HRMS [M-OH]^+^ ion at *m*/*z* 221.1905. The bicyclic structure was evidenced by considering this information together with the ^1^H NMR and ^13^C NMR data (Table 1 and Table 2). Furthermore, partial structures in this compound were inferred after the combined analysis of the ^1^H, ^13^C, HSQC, and 1D TOCSY NMR spectra (see Supporting Information, Figures S22–S24 and S26) (Figure 3a). Assignment of the two sp^3^ quaternary carbons was realized, as shown based on the chemical shifts in the two singlet methyl groups (δ 0.88 and 1.01 ppm ^1^H). Also worthy of mention was the belonging of the methyl group at δ 1.68 ppm ^1^H and the olefinic proton at δ 5.61 ppm ^1^H to the same spin system, as inferred from the analysis of the 1D TOCSY spectrum obtained after irradiating at δ 2.26 ppm ^1^H (see Figure S26 at Supporting Information).

**Figure 4:**
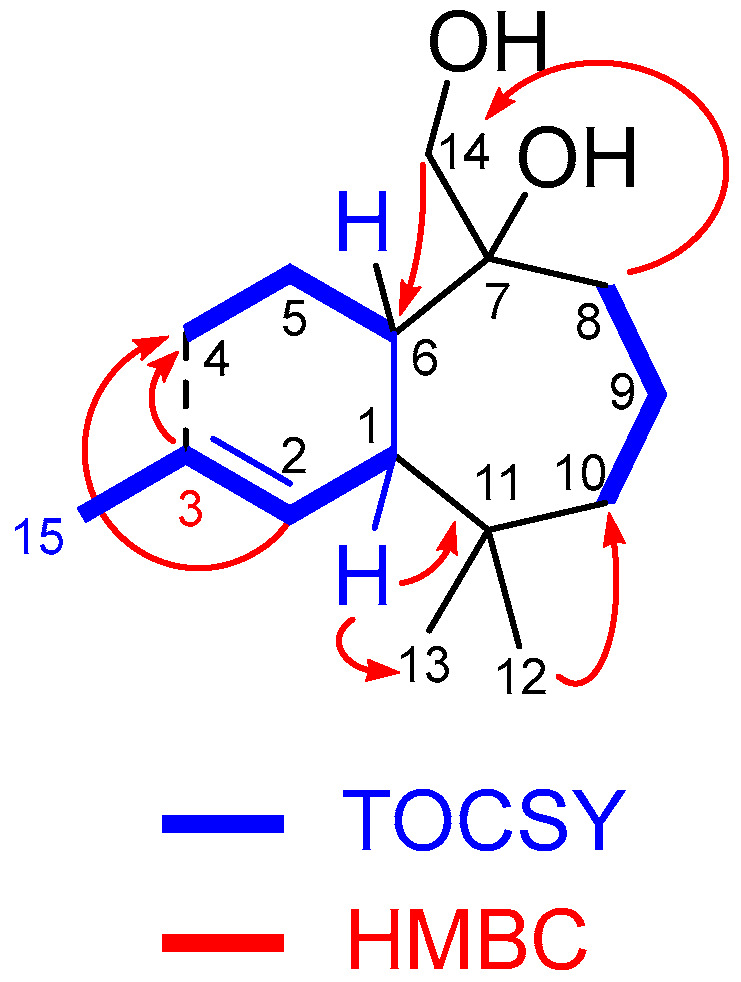
Key TOCSY and HMBC correlations for compound **6**.

Finally, the entire planar structure of **6** was elucidated after analyzing the correlations observed in the HMBC spectrum (see Figure S25 at Supporting Information), which allowed us to bond partial structures, as shown in Figure 4. Key HMBC correlations were those observed between H14 and C6, H1 and C11, H8 and C14, and H12 and C10 (Figure 4).

**Figure 5:**
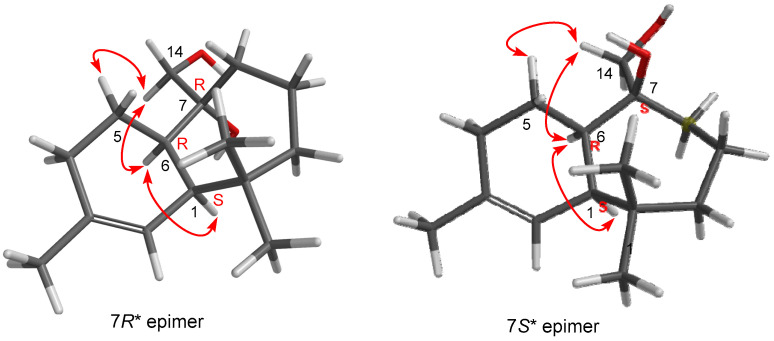
NOEs observed for compound **6** and their analysis in the most stable conformations of the two possible C7 epimers of this compound.

The analysis of 1D NOE spectra (see Figures S27 and S28 at Supporting Information) revealed a *cis*-bonding of the bicyclic system, as evidenced by the correlation between protons H1 and H6, indicating that they were located on the same side of the molecule. Additionally, clear NOEs were observed between H14 and H5 as well as H6. Considering the known conformational mobility of seven-membered rings, we proceeded to computationally determine the most stable conformation of each epimer of **6** (7R* and 7S*, Figure 5) (see Tables S1–S9 in the Supporting Information) and analyze the NOEs observed in these conformations. As shown in Figure 5, the most stable conformations of both epimers at C6 exhibited a spatial disposition consistent with the observed NOEs.

This inconclusive result prompted us to explore alternative methods to assign the configuration at C-7 of compound **6**. GIAO (gauge-independent atomic orbital) NMR chemical shift calculations have become a reliable method to aid in the stereo-structural elucidation of new natural products [33,34]. In addition, this methodology has made it possible to carry out structural revisions of numerous natural products [35]. Due to the flexibility of the seven-membered ring and the dependence of the conformation on chemical shifts, we applied the efficient protocol developed by Hehre et al. [36] to calculate chemical shifts for flexible natural products. Spartan’24 implemented a neural network routine within the multistep NMR spectrum task developed by Hehre et al., which considerably improves its performance and the accuracy of the obtained results.

After building compound **6** (C-7 R*) and its epimer (C-7 S*) at C-7 in Spartan’24, both stereoisomers were subjected to the NMR calculation protocol developed by Hehre et al., which is reported in detail in Section 4. This protocol allows for the prediction of ^13^C chemical shifts with an accuracy quantified using an overall rms (root mean square). The calculated rms deviation between the experimental and calculated ^13^C chemical shifts for compound **6** (C-7 R*) was 1.3 ppm, with a max. absolute of 2.7 corresponding to C-9, whereas for the **6** epimer (C-7 S*) the statistical rmsd term was 2.4, with a max. absolute of 3.7 (Table 3). These values definitively confirm the relative configuration for this molecule, as shown in Figure 5. In fact, the DP4 probability scores, calculated according to Goodman’s procedure [37], provide a value of 100% for the R*-epimer**.** Based on these results, R* was predicted to be the relative configuration at C7 of compound **6**.

Once the spatial disposition of the natural product was computationally established, the observed NOE effects in the corresponding 1D NOE experiments confirmed the conformation proposed for **6** (Figure 5) and consequently the relative 7*R* configuration for compound **6**.

As happened with **5**, the value of the specific rotation of compound **6** was calculated computationally. The optical rotation was computed on the two most populated conformations, M02 and M04 (see Tables S6 and S7 at the Supporting Information), to give an average [α]_D_ value of +86.27 at a wavelength of 589 nm, which again shares the sign with the value calculated experimentally ([α]_D_ = +53.26), allowing us to propose the absolute stereochemistry of this compound as 1*S*6*R*7*S*.

**Figure 6:**
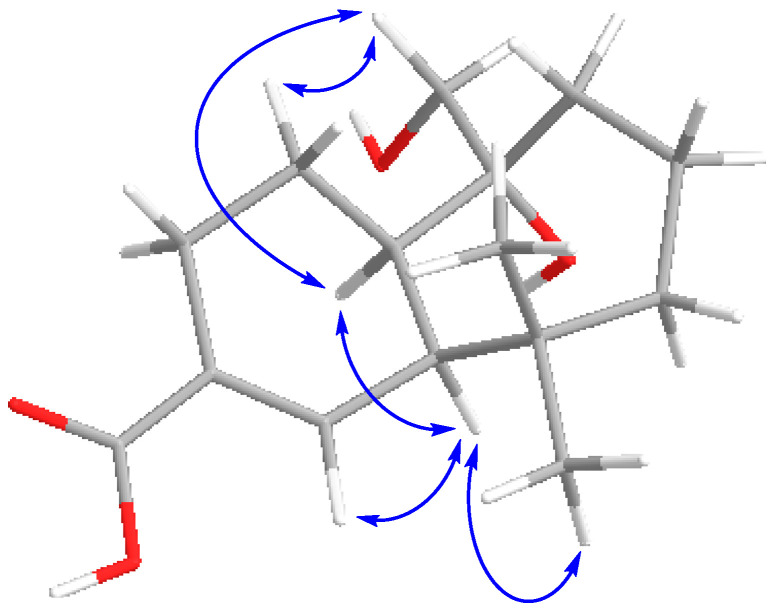
Selected 1D NOE correlations for compound **8**.

Finally, the molecular formula of the new natural product **8** was C_15_H_24_O_4_, as deduced from HRMS [M-OH]^+^ *m*/*z* 293.1751. This compound was identified by comparing its ^1^H and ^13^C NMR data with those of compound **6** (Table 1 and Table 2). Thus, while the C15 methyl in compound **6** (δ 1.68 ppm ^1^H, 26.60 ppm ^13^C) was no longer observed, a new carbonyl signal was observed at δ 171.61 ppm in ^13^C. These data, together with the significant deshielding experienced by H2 (from δ 5.61 ppm ^1^H in compound **6** to 7.32 ppm ^1^H in **8**), prove the existence of a carboxylic acid at C3. As expected, compound **8** shared with diol **6** the same relative conformation, as deduced from the analysis of the corresponding 1D NOE spectra (Figure 6). Thus, NOE correlations were observed from H6 to H14a and H1 see Figures S44 and S45 at Supporting Information. Himachalane **8** was proposed to possess the same absolute configuration as himachalane **6**.

Noteworthy is the fact that this is the first study reporting the occurrence of himachalane sesquiterpenes *D. viscosa*. In this sense, some of this class of natural products have been described as possessing interesting biological activities such as antifungal [38] or insecticidal [39] activities.

### 2.3. Potential Plant Protection Effects

The compounds isolated from *D. viscosa* roots **1**+**2**+**3**, **4**, **5** and the derivative **6a** were tested against insect pests (*R. padi*, *M. persicae*, *S. littoralis*), the root-knot nematode *M. javanica* and the plants ryegrass and lettuce.

Table 4 shows the insect antifeedant effects of the compounds tested (**1**–**3**, **1**, **4**, **5**–**6**, **5** and **6a**). All these molecules showed species-dependent antifeedant effects except the monoterpene alcohol **4**, the effects of which were below the established threshold for dose–response experiments (70% for antifeedant effects at the maximum dose tested). *S. littoralis* was moderately affected by the mixture **1**+**2**+**3** (EC_50_ = 32.35 μg/cm^2^) and by himachalane **6a** (EC_50_ = 22.10 μg/cm^2^), while **1** was inactive*. M. persicae* was the most sensitive insect, affected by the mixture **1**+**2**+**3** (EC_50_ = 16.54 μg/cm^2^) and compounds **1** (EC_50_ = 19.17 μg/cm^2^) and **5** (EC_50_ = 18.71 μg/cm^2^), while *R. padi* was only affected by phenol derivatives **1**+**2**+**3** (EC_50_ = 19.14 μg/cm^2^) and **1** (EC_50_ = 21.77 μg/cm^2^).

Benzyl esters **1**–**3** are thymol derivatives. The activity ranked as follows: mixture **1**:**2**:**3** (rich in 3, 1:3:7) > **1** (inactive to *S. littoralis* and similarly active to both aphids) > 4, indicating that the presence of a carbonyl group in C7 was important for the antifeedant effects observed here. The positive control thymol has been previously reported as an effective antifeedant to *S. littoralis*, *M. persicae* and *R. padi* (EC_50_ values of 21.0, 7.6 and 18.6 μg/cm^2^ respectively), while its precursor *p*-cymene was inactive [40]; however, this is the first report on the antiffeedant effects of these types of phenols. Among the sesquiterpenes, the guaianolide sesquiterpene lactone **5** was active on *M. persicae*. Similarly, the structurally related sesquiterpene lactone tomentosin was the active compound present in *D. viscosa* aerial parts, with antifeedant effects against *M. persicae* [41], while α- and γ-costic acids were identified in an insecticidal fraction of *D. viscosa* with contact toxicity against *Sitophilus granarius* adults [9]. Himachalane **6a** was antifeedant to *S. littoralis* and *M. persicae* respectively. Himachalanes have been reported as being active on insects. For example, essential oil from *Cedrus deodara*, rich in β-himachalene (46%), was deterrent to *Plutella xylostella* [42]. Furthermore, himachalol and β-himachalene were toxic to the pulse beetle *Callosobruchus analis* [39].

The nematicidal effects of the compounds tested are shown in Table 5. Phenol derivative **4** was nematicidal against *M. javanica* J2. Compound **4** did not show a dose–response experiment with a minimal lethal dose (MLD) of 0.5 mg/mL, while the positive control thymol had an MLD of 0.25 with effective doses (LD_50_ and LD_90_) of 0.13 and 0.22 mg/mL.

Phenols with a carboxyl group, such as salicylic and cumic acid, resulted in being effective nematicidal compounds, followed by isopropyl salicylate and alkylated phenols with a *p*-isopropyl group (thymol and carvacrol). The nematicidal action of these compounds was further confirmed by their egg-hatching-inhibitory effects [43]. Previous results have shown the nematicidal effect of extracts from *D. viscosa* aerial parts containing costic and isocostic acids [44]. However, this is the first report on the presence of compounds with promising nematicidal activity in *D. viscosa* roots.

**Figure 7:**
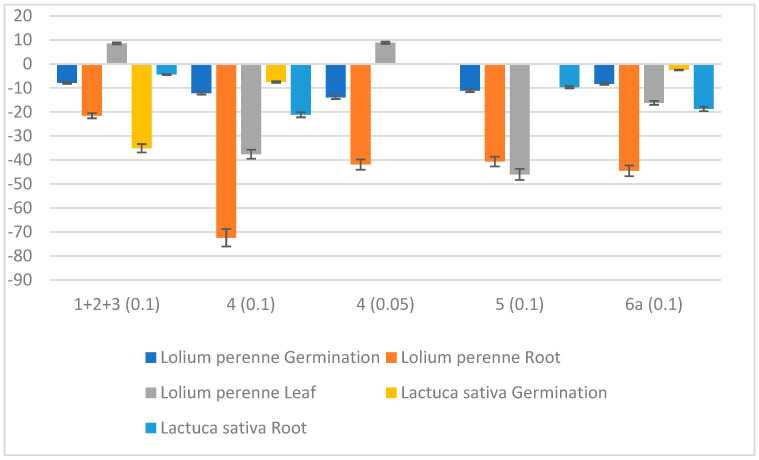
Phytotoxicity (% relative to the control) of the compounds tested (**1**+**2**+**3**, **4**, **5** and **6a**) against *Lolium perenne* and *Lactuca sativa.* The dose tested was 0.1 mg/mL, and this was lower (0.05) for the active compounds (until the effects were < 50%).

*D. viscosa* root compounds (**1**+**2**+**3**, **4**, **5** and **6a**) were tested for phytotoxic effects against two model plant species, the monocotyledoneous ryegrass and the dycotiledoneous lettuce (*L. perenne* and *L. sativa*). All phytotoxic parameters measured for *L. sativa* had inhibition values < 20% (Figure 7), and therefore, none of these compounds were considered phytotoxic to this plant. On the contrary, phytotoxic effects were found against *L. perenne*. Phenol derivative **4** showed dose-dependent phytotoxicity (72 and 37% root and leaf growth inhibition at 0.1 mg/mL, 42% root growth inhibition at 0.05 mg/mL), followed by **5** (41 and 46% root and leaf growth inhibition, 0.1mg/mL) and **6a** (44.5 and 16% at 0.1 mg/mL) (Figure 7).

The allelopathic effects of *D. viscosa* leaf extracts and dry biomass have been described [45,46]. The phytotoxic compounds identified included sesquiterpene lactones (inuloxins A, C and D) and α-costic acid, with herbicidal effects against parasitic weeds (*Orobranche crenata* and *Cuscuta campestris*) [47], and the dihydroflavanol 3-O-acetylpadmatin, which showed inhibition of radicle growth of *Orobranche cumana* and *Phelipanche ramosa* [48]. The inhibition of root elongation and growth of *Lycopersicon esculentum* and *Lepidium sativum* caused by inuloxin A was attributed to an alteration in the cell redox system [49]. Furthermore, thymoxyacetic acid was phytotoxic in preemergence and thymol in postemergence for five plant species, including *L. sativa* [50]. However, this is the first report on the allelopathic effects of *D. viscosa* root compounds **4**, **5** and **6a**.

## 3. Discussion

*Dittrichia viscosa* is a plant well known for its use in traditional medicine. Given its wide range of activities and the limited research on its roots, we focused our study on analyzing the composition of *D. viscosa* roots and evaluating the pesticidal activity of the isolated compounds.

Roots of *D. viscosa* were revealed to be a source of new natural compounds. The structures of these compounds were determined. Four new natural products were found, two of them being new himachalane sesquiterpenes—a scarce group of sesquiterpenes, with, to the best of our knowledge, less than twenty himachalanes (including *seco*-himachalanes) being reported so far. Noteworthy is the fact that the stereochemistry of these compounds was assigned with the aid of recently described computational protocols.

It is noteworthy that the terpenes found in the roots of *D. viscosa* are not found in other parts of the plant, which increases the structural diversity found in this species, a fact that could extend to other species.

Finally, the pesticidal activity of *D. viscosa* was assessed, and different compounds have shown interesting results as potential antifeedants against *Spodoptera littoralis*, *Myzus persicae* and *Rhopalosiphum padi* (pest insects) and as potential nematicides against *Meloidogyne javanica*. Additionally, some compounds showed allelopathic activity against the monocotyledonous *Lolium perenne*. Notably, to our knowledge, this is the first report on the biological activity of natural products with a himachalane skeleton, which may help to explain their biosynthesis in plants.

## 4. Materials and Methods

### 4.1. General Experimental Procedures

Optical rotations were measured on a Perkin-Elmer 141 polarimeter (Perkin-Elmer, Shelton, CT, USA). IR spectra were recorded on a FT/IR-6200 spectrometer (JASCO Inc., Easton, MD, USA). NMR spectra were acquired on BRUKER Avance NEO (^1^H NMR 600 MHz), Varian Direct Drive (^1^H NMR 500 MHz/^13^C NMR 125 MHz) and BRUKER Nanobay Avance III HD (^1^H NMR 400 MHz) spectrometers (Bruker, Billerica, MA, USA). Signal multiplicities are reported using the following abbreviations: s = singlet, d = doublet, t = triplet, q = quartet, quint = quintuplet, hex = hexuplet, hep = heptuplet, bs = broad singlet, bd = broad doublet, bt = broad triplet, dd = doublet of doublets, dt = doublet of triplets, dq = doublet of quartets, dquint = doublet of quintets, td = triplet of doublets, ddd = doublet of doublets of doublets, m = multiplet. High-resolution mass spectra (HRMS) were determined on a BRUKER Autoflex mass spectrometer (Bruker, Billerica, MA, USA). Silica gel 60A (60−200 μm) (Thermo Scientific, Geel, Belgium) was used for column chromatography. Chromatography fractions and the acetylation reaction were monitored by thin-layer chromatography carried out on 0.25 mm E. Merck silica gel plates (60F-254) (Sigma Aldrich, St. Louis, MO, USA) using UV light and a solution of phosphomolybdic acid in ethanol as the visualizing agent. Semipreparative HPLC separation was carried out on a column (5 μm silica, 10 × 250 mm^2^) at a flow rate of 4.0 mL/min in an Agilent Series 1100 instrument (Agilent Technologies, Santa Clara, CA, USA).

### 4.2. Plant Material, Extraction and Isolation

Specimens of *Dittrichia viscosa* (voucher GDA54164) were collected in Granada (37.208108, −3.622045, Spain) in September 2022.

Roots of *D. viscosa* were air-dried for 5 days and subsequently crushed (368 g). Then, the plant material was extracted using a Soxhlet apparatus with hexane for 24 h, affording 2.6 g of extract (0.7% yield from dry roots), followed by MTBE, resulting in 2.9 g of extract (0.8% yield from dry roots).

A hexane extract portion (875 mg) was fractionated by column chromatography on silica gel using mixtures of H/MTBE of increasing polarity as an eluent to, finally, obtain four fractions (FH1–FH4). Fraction FH1 (409 mg) was obtained using H/MTBE (6:1) as an eluent and contained a mixture of geraniol, fatty acid biosynthetic derivatives and compounds **1**, **2** and **3**. FH1 was re-chromatographed using H/MTBE (95:5) to separate a mixture of compounds **1**, **2** and **3** in a 1:3:7 ratio (283 mg). A 15 mg fraction of this mixture was subjected to semipreparative HPLC (normal phase, H/MTBE 9:1) to obtain pure **1** (Rt = 8.1 min, 0.8 mg), **2** (Rt = 8.5 min, 1.9 mg) and **3** (Rt = 9.0 min, 4.4 mg). The remaining material consisted of different mixtures of these compounds. Fraction FH2 (248 mg), obtained using H/MTBE (3:1), contained a complex mixture of products, with fatty acid biosynthetic derivatives again being the main components. Fraction FH3 (156 mg), obtained using H/MTBE (1:1), was mainly composed of sterols, fatty acid biosynthetic derivatives and compound **4**. Re-chromatography of this fraction with H/MTBE (2:1) led to the isolation of 46 mg of pure compound **4**. FH4 (52 mg) was collected with MTBE and constituted fatty acid biosynthetic derivatives and compounds **5** and **6**. FH4 was subjected to a second round of chromatography (H/MTBE (2:1)) to obtain a 2:1 mixture of **5** and **6** (19 mg). A 15 mg portion of this mixture was subjected to semipreparative HPLC (normal phase, H/MTBE 2:1) to give pure **5** (Rt= 8.0 min, 4.7 mg) and **6** (Rt= 9.6 min, 2.6 mg). The remaining material consisted of mixtures of compounds **5** and **6**.

The MTBE extract (2.9 g) was also fractionated by column chromatography eluting with mixtures of H/MTBE of increasing polarity, affording nine fractions (FE1-FE9). Fraction FE1 (313 mg) was obtained using H/MTBE (4:1) as an eluent and was mainly composed of a mixture of fatty acid biosynthetic derivatives, geraniol and compounds **1**–**3**. Fraction FE5 (380 mg) was collected employing H/MTBE (1:1) and constituted a mixture of fatty acid biosynthetic derivatives and compounds **4**–**6**. FE5 was re-chromatographed using H/MTBE (2:1) to obtain 58 mg of compound **4** and 140 mg of a 2:1 mixture of **5** and **6**. Finally, fraction FE7 (120 mg), obtained using MTBE, mainly constituted a mixture of fatty acid biosynthetic derivatives and compounds **7** and **8**. FE7 was re-chromatographed with H/MTBE (1:3), obtaining 20 mg of a 2:1 mixture of **7** and **8**. This mixture was subjected to semipreparative HPLC (normal phase, H/MTBE 1:4) to give 4.8 mg of pure **7** (Rt = 11.6 min), 3.2 mg of **8** (Rt = 13.8 min) and 3.9 mg of a mixture of **7** and **8**.

### 4.3. Derivatization of Natural Product 6

Acetylation of a mixture of **5** and **6** was performed in order to facilitate their separation. A mixture of compounds **5** and **6** (145 mg, 2:1 ratio) was dissolved in acetic anhydride (1 mL) and pyridine (1 mL) under an inert atmosphere stirring at 25 °C for 50 min. Then, the reaction content was mixed with ice, and 30 mL of MTBE was added. Once the ice melted, the two phases were separated, and the organic phase was washed with 2N HCl (15 mL × 3), saturated NaHCO_3_ solution (15 mL × 3) and brine (15 mL × 3) and dried with anhydrous Na_2_SO_4_. The solvent was removed, giving a residue, which was flash-chromatographed (H/MTBE, 3:2) to give 87 mg of **5** and 46 mg of **6a**.

### 4.4. Spectroscopical Data (^1^H and ^13^C NMR) of Natural Products 1–8

Compound **1**. Colorless oil. [α]_D_^25^ = 0.00 (*c* 1.0, DCM). HRMS [M + H]^+^ *m*/*z* 265.1802 (calcd for C_16_H_25_O_3_, 265.1798). IR (ATR) ν_max_ 2962, 2936, 2875, 1732, 1613, 1582, 1508, 1447, 1419, 1259, 1146, and 1042 cm^−1^. ^1^H NMR (400 MHz, CDCl_3_) δ 7.19 (d, *J* = 7.7 Hz, 1H), 6.91 (dd, *J* = 7.7 Hz, 1.7 Hz, 1H), 6.82 (d, 1.7 Hz, 1H), 5.09 (s, 2H), 3.83 (s, 3H), 3.30 (hept, *J* = 6.9 Hz, 1H), 2.43 (hex, *J* = 7.0 Hz, 1H) 1.71 (dquint, *J* = 14.8 Hz, 7.4 Hz, 1H), 1.50 (dquint, *J* = 14.8 Hz, 7.4 Hz, 1H) 1.20 (d, *J* = 6.9 Hz, 6H), 1.17 (d, *J* = 7 Hz, 3H), 0.91 (t, *J* = 7.4 Hz, 3H). ^13^C NMR (101 MHz, CDCl_3_) δ 176.64, 156.83, 137.02, 134.68, 126.11, 120.26, 110.11, 66.08, 55.36, 41.09, 26.81, 26.60, 22.63, 22.63, 16.62, 11.64.

Compound **2**. Colorless oil. ^1^H NMR (400 MHz, CDCl_3_) δ 7.19 (d, *J* = 7.7 Hz, 1H), 6.91 (d, *J* = 7.7 Hz, 1H), 6.82 (s, 1H), 5.08 (s, 2H), 3.83 (s, 3H), 3.30 (hept, *J* = 6.9 Hz, 1H), 2.24 (d, *J* = 6.9 Hz, 2H) 2.13 (hept, *J* = 6.9 Hz, 1H), 1.20 (d, *J* = 6.9 Hz, 6H), 0.96 (d, *J* = 6.9 Hz, 6H). ^13^C NMR (101 MHz, CDCl_3_) δ 173.04, 156.85, 137.13, 134.51, 126.13, 120.48, 110.32, 66.17, 55.37, 43.46, 26.61, 25.75, 22.63, 22.43.

Compound **3**. Colorless oil. ^1^H NMR (400 MHz, CDCl_3_): δ 7.19 (d, *J* = 7.7 Hz, 1H), 6.91 (d, *J* = 7.7 Hz, 1H), 6.82 (s, 1H), 5.08 (s, 2H), 3.83 (s, 3H), 3.30 (hept, *J* = 6.9 Hz, 1H), 2.60 (hept, *J* = 6.9 Hz, 1H), 1.20 (d, *J* = 7.0 Hz, 12H). ^13^C NMR (101 MHz, CDCl_3_) δ 177.02, 156.85, 137.03, 134.67, 126.12, 120.23, 110.08, 66.17, 55.36, 34.05, 26.62, 22.64, 19.02.

Compound **4**. Colorless oil. ^1^H NMR (400 MHz, CDCl_3_): δ 7.19 (d, 1H), 6.91 (d, 1H), 6.90 (s, 1H), 4.66 (s, 2H), 3.85 (s, 3H), 3.31 (hept, *J* = 7.0 Hz, 1H), 1.21 (d, *J* = 7.0 Hz, 6H). ^13^C NMR (126 MHz, CDCl_3_): δ 157.01, 139.45, 136.62, 126.12, 119.07, 109.18, 65.55, 55.39, 26.58, 22.69.

Compound **5**. Colorless oil. [α]_D_^25^ = −40.12 (*c* 1.0, DCM). IR (ATR) ν_max_ 3440, 2933, 2870, 1761, 1706, 1641, 1458, 1381, 1258, 1207, 1074, 1021, 985, and 894 cm^−1^. HRMS [M + H]^+^ *m*/*z* 249.1491 (calcd for C_15_H_21_O_3_, 249.1485). ^1^H and ^13^C NMR data is shown in Table 1 and Table 2.

Compound **6**. Colorless oil. [α]_D_^25^ = +53.26 (*c* 0.3, DCM). IR (ATR) ν_max_ 3359, 2916, 1709, 1440, 1373, 1268, 109, and 795 cm^−1^. HRMS [M-OH]^+^ *m*/*z* 221.1905 (calcd for C_15_H_25_O, 221.1900). ^1^H and ^13^C NMR data is shown in Table 1 and Table 2.

Compound **6a**. Colorless oil. [α]_D_^25^ = +16.32 (*c* 1.0, DCM). ^1^H NMR (500 MHz, CDCl_3_): δ 5.61 (bd, *J* = 4.6 Hz, 1H), 4.07 (d, *J* = 11.3 Hz, 1H), 3.94 (d, *J* = 11.3 Hz, 1H), 2.29 (bs, 1H), 2.10 (s, 3H), 2.01−1.87 (m, 4H), 1.76−1.53 (m, 5H), 1.68 (s, 3H), 1.47−1.41 (m, 1H), 1.38 (ddd, *J* = 14.2, 5.8, 4.0 Hz, 1H), 1.00 (s, 3H), 0.87 (s, 3H). ^13^C NMR (126 MHz, CDCl_3_): δ 171.22, 133.46, 125.47, 76.42, 71.54, 46.06, 43.36, 40.79, 38.44, 33.64, 31.32, 30.82, 26.00, 23.61, 21.51, 20.98, 19.44. HRMS [M-OH]^+^ *m*/*z* 293.1751 (calcd for C_17_H_25_O_4_, 293.1747).

Compound **7**. Colorless oil. ^1^H NMR (500 MHz, CDCl_3_): δ 5.01 (s, 1H), 4.95 (s, 1H), 4.39 (td, *J* = 10.4, 5.0 Hz, 1H), 3.18 (dd, *J* = 15.7, 5.0 Hz, 1H), 2.71 (quint, *J* = 7.5 Hz, 1H), 2.54 (dd, *J* = 15.7, 10.3 Hz, 1H), 2.23 (q, *J* = 10.0, 9.4 Hz, 1H) 2.12 (m, 1H), 2.00−1.85 (m, 2H), 1.85−1.65 (m, 4H), 1.63 (m, 1H) 1.22 (d, *J* = 7.4 Hz, 3H), 1.20 (s, 3H).

Compound **8**. Colorless oil. [α]_D_^25^ = + 22.09 (*c* 0.2, DCM) HRMS [M-OH]^+^ *m*/*z* 251.1647 (calcd for C_15_H_23_O_3_, 251.1642). ^1^H and ^13^C NMR data is shown in Table 1 and Table 2.

### 4.5. Computational Calculations of ^13^C NMR Spectrum and Optical Rotation

Calculations were performed with Spartan’24 (Wavefunction Inc., Irvine, CA, USA). To obtain the ^13^C NMR data by computational calculation, the automated protocol implemented in Spartan’24 was followed [36]. This protocol consists of six steps: (I) a systematic conformational search using MMFF molecular mechanics, eliminating duplicate conformers and those with energy 40 kJ/mol above the global minimum; (II) geometric calculation using HF/3-21G, also eliminating duplicate conformers and those with energy higher than 40 kJ/mol above the global minimum; (III) energy calculation with the ωB97X-D/6-31G* model and removal of conformers above 15 kJ/mol with respect to the global minimum; (IV) geometric calculation with the ωB97X-D/6-31G* model and removal of conformers with energies higher than 10 kJ/mol from that of the global minimum; (V) energy calculation with ωB97X-V/6-311 + G(2df,2p)[6-311G*], and finally; (VI) NMR calculations (following calculation of Boltzmann weights for conformationally flexible molecules) using the ωB97X-D/6-31G* method that has been corrected empirically based on the comparison of calculated and experimental ^13^C shifts for ~2000 rigid molecules. These corrections are on the order of 1−3 ppm.

Finally, compounds **5** and **6** in the best-fit conformer between experimental and calculated ^13^C NMR data were reoptimized with Gaussian 16 using DFT at the wb97xd/6-311 + g(2d,p) level of theory [51,52], and the [α]_D_ optical rotation was computed at the same level of theory [53]. To simulate the effect of the solvent used for the experimental measurements (dichlorometane), the IEFPCM model was considered [54].

### 4.6. Biopesticide Assays

#### 4.6.1. Antifeedant Activity

The insect colonies (*Spodoptera littoralis*, *Myzus persicae* and *Rhopalosiphum padi*) came from laboratory colonies reared on an artificial diet and host plants *(Capsicum annuum*, *Hordeum vulgare*), respectively, at 22 ± 1 °C, >70% relative humidity and a 16:8 h (L:D) photoperiod at ICA-CSIC.

The tests have been described before [43]. Briefly, the upper surface of leaf disks or fragments (1.0 cm2) of *C. annuum* and *H. vulgare* were treated with 10 µL of compound at an initial dose of 5 µg/µL (50 µg/cm^2^). Two sixth-instar *S. littoralis* larvae (>24 h after molting) per Petri dish (5 unities) or 10 apterous aphid adults (24–48 h old) placed in a 2 × 2 cm ventilated plastic box (20 unities) were allowed to feed at room temperature or in the growth chamber respectively. The experiments ended at 75% larval consumption of paired control or treatment disks for *S. littoralis* or after 24 h for aphids. Each experiment was repeated 2 times. Feeding inhibition (%FI), based on the disk surface consumption (digitalized with https://imagej.nih.gov/ij/ (accessed on 12 January 2026) [55], and aphid settling inhibition (%SI), based on the number of aphids on each leaf fragment, were calculated as % FI/SI = [1 − (T/C) × 100], where T and C represent feeding/settling on treated and control leaf disks, respectively. The significance of these effects was analyzed by the nonparametric Wilcoxon paired signed-rank test. Tests with an FI/SI > 70% were further tested in dose–response experiments (range of activities between 100 and <50%, minimum of 3 doses) to calculate their effective EC_50_ dose from linear regression analysis (% FI/SI on Log-dose, STATGRAPHICS Centurion XVI, version 16.1.02). Thymol (T0501, Sigma-Aldrich, St. Louis, MO, USA e) was included as a positive control.

#### 4.6.2. Nematicidal Activity

A *Meloidogyne javanica* population was maintained on *Solanum lycopersicum* plants (var. Marmande) in pot cultures at 25 ± 1 °C and 70% relative humidity. Egg masses of *M. javanica* were handpicked from infected tomato roots. Second-stage juveniles (J2) were obtained from hatched eggs by incubating egg masses in a water suspension at 25 °C for 24 h.

The tests were carried out as described by Andres et al. [56]. Briefly, the compounds were dissolved in distilled water containing 5% of a DMSO–Tween solution (0.5% Tween 20 in DMSO) and evaluated. The initial concentration tested was 0.5 µg/µL. The nematicidal activity data are presented as percentage dead J2 corrected according to Scheider–Orelli’s formula. Effective lethal doses (LC_50_ and LC_90_) were calculated by Probit analysis. Five serial dilutions were used to obtain the LC_50_ and LC_90_, and four replicates were used in each concentration. When dose experiments did not follow a dose–response curve, minimal lethal doses, MLD, to give 100% mortality were used. Thymol (T0501, Sigma-Aldrich) was included as a positive control.

#### 4.6.3. Phytotoxic Effect

The experiments were conducted with *Lactuca sativa* cv. Teresa and *Lolium perenne* Nui seeds (donated by Fito-España and Battle-España, respectively). Filter paper disks (2.5 cm diam.) with 20 µL of the solvent (control) or the test compound (5 mg/mL in EtOH) were placed on 12-well plates (3 replicates/experiment), and then 500 mL H_2_O/well and 10/5 seeds (*L. sativa*/*L. perenne* pre-soaked in distilled water for 12 h) were added to give a final concentration of 0.1 mg/mL in the well. Then, the plates were covered and placed in a plant growth chamber (25 °C, 70% RH, 16:8 L:D).

Germination was monitored for 6 days, and the root/leaf length was measured at the end of the experiment (25 seedlings randomly selected for each experiment) with the application ImageJ 1.54p (http://rsb.info.nih.gov./ij/ (accessed on 5 January 2026)) [55]. Serial dilutions (1:2) were carried out for tests resulting in an inhibition > 50% (with respect to the control) for any parameter measured [56].

## Figures and Tables

**Table 1:** ^1^H NMR spectroscopic data for compounds **5**, **6** and **8** in CDCl_3_ (δ in ppm, *J* in Hz).

Position	5	6	8
1	2.23, m	2.23, bs	2.58, bs
2a	1.93, dt (11.1, 5.3)	5.61, dd (5.6, 2.2)	7.32, d (6.0)
2b	1.69, m		
3a	2.40, m		
3b	2.33, m		
4a		2.01, bd (8.2)	2.43, bd (18.6)
4b			2.26, dt (18.6, 9.0)
5a	2.24, m	1.70, m	1.71, m
5b		1.44, m	1.57, m
6	3.47, bt (9.5)	1.96, m	1.97, d (13.3)
7	2.34, td (10.5, 7.6)		
8a	4.54, td (11.1, 3.4)	1.64, m	1.66, m
8b		1.54, m	1.54, m
9a	3.17, dd (16.3, 3.1)	1.69, m	1.75, m
9b	2.63, dd (16.3, 11.6)		
10a		1.90, m	2.02, m
10b		1.42, dq (14.4, 5.0)	1.46, ddd (14.2, 6.6, 3.3)
11	2.92 p (7.7)		
12		1.01, s	1.12, s
13a	1.34, d (7.7)	0.88, s	0.89, s
13b			
14a	4.93, bs	3.49, d (10.8)	3.57, d (11.0)
14b	4.91, bs	3.39, d (10.8)	3.39, d (11.0)
15a	5.10, bs	1.68, bs	
15b	4.99, bs		

**Table 2:** ^13^C NMR spectroscopic data for compounds **5**, **6** and **8** in CDCl_3_ (δ in ppm).

Position	5	6	8
1	45.93 (CH)	43.63 (CH)	43.78 (CH)
2	30.06 (CH_2_)	125.31 (CH)	146.49 (CH)
3	34.20 (CH_2_)	133.52 (C)	128.93 (C)
4	152.50 (C)	31.22 (CH_2_)	25.38 (CH_2_)
5	54.94 (CH)	21.27 (CH_2_)	20.42 (CH_2_)
6	71.19 (CH)	45.86 (CH)	45.92 (CH)
7	54.83 (CH)	77.49 (C)	77.22 (C)
8	76.66 (CH)	30.49 (CH_2_)	31.36 (CH_2_)
9	39.64 (CH_2_)	19.53 (CH_2_)	19.27 (CH_2_)
10	144.08 (C)	41.05 (CH_2_)	41.38 (CH_2_)
11	39.06 (CH)	38.47 (C)	38.84 (C)
12	179.14 (C)	33.73 (CH_3_)	33.50 (CH_3_)
13	10.48 (CH_3_)	25.74 (CH_3_)	27.04 (CH_3_)
14	110.67 (CH_2_)	70.13 (CH_2_)	69.65 (CH_2_)
15	109.61 (CH_2_)	26.60 (CH_3_)	171.61 (C)

**Table 3:** Theoretical ^13^C NMR data of the two possible C-7 epimers of compound **6** and their comparison with the experimental values.

	Theoretical ^13^C NMR (δ ppm)				Experimental ^13^C NMR (δ ppm)
Carbon	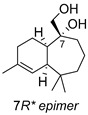		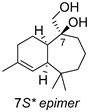		
	Theory	Difference	Theory	Difference	
C1	43.9	0.3	43.6	0.0	43.63
C2	127.8	2.5	127.7	2.4	125.29
C3	133.6	0.1	134.1	0.6	133.50
C4	30.6	−0.6	29.8	−1.4	31.22
C5	22.2	0.9	24.6	3.3	21.27
C6	44.9	−1	42.7	−3.2	45.88
C7	77.5	0	77.0	−0.5	77.49
C8	29.6	−0.9	34.2	3.7	30.50
C9	20.9	−2.7	20.0	−3.6	23.61
C10	39.1	−2	43.1	2	41.06
C11	37.9	−0.6	36.6	−1.9	38.48
C12	24.4	−1.3	25.8	0.1	33.72
C13	33.9	0.2	31.0	−2.7	25.74
C14	71.1	1	69.4	−0.7	70.13
C15	23.9	0.3	23.8	0.2	23.61
RMSD		1.3		2.4	
Maximal absolute		2.7		3.7	
DP4	100%		0%		
Nº of conformations		14		15	

Maximal absolute expresses the largest deviations between the calculated and experimental chemical shifts; rmsd is a statistical parameter: δ_13C_ root mean square deviation. The DP4 (%) probability scores were calculated for the two epimers according to Goodman’s procedure.

**Table 4:** Insect antifeedant effects against *Spodoptera littoralis*, *Myzus persicae* and *Ropalosiphum padi* of compounds **1**+**2**+**3**, **1**, **4**, **5** and **6a**. Thymol is included as a positive control.

Compound	μg/cm^2^	*S. littoralis*	*M. persicae*	*R. padi*
		%FI ^2^ (N = 6–10)	%SI ^2^ (N = 20)	
**1**+**2**+**3**	50	73.13 ± 10.01	70.33 ± 7.72	71.30 ± 6.12
	EC_50_^1^	32.35 (23.82–43.93)	16.54 (9.17–29.84)	19.14 (11.41–32.08)
**1**	50		71.90 ± 4.42	71.78 ± 4.40
	EC_50_		19.17 (14.26–25.75)	21.77 (15.89–29.85)
**4**	50	41.70 ± 8.91	57.65 ± 8.49	56.53 ± 8.03
	EC_50_^1^	>50	≈50	≈50
**5**	50	3.60 ± 3.65	81.69 ± 5.21	65.41 ± 5.45
	EC_50_^1^	>50	18.71 (14.00–25.01)	≈50
**6a**	50	86.69 ± 4.26	55.31 ± 7.43	52.45 ± 7.38
	EC_50_^1^	22.10 (15.34–31.84)	>50	≈50
Thymol	50	52.4 ± 10.1	81.8 ± 7.7	92.1 ± 2.6
	EC_50_	∼50	17.6 (14.1–18.7)	18.6 (4.1–23.3.5)

^1^ EC_50_: effective dose (μg/cm^2^) to give 50% effect (95% Confidence Limits). ^2^ %FI/SI = [1 − (consumption/settling on treated disk/consumption/settling on control disk)] × 100.

**Table 5:** In vitro nematicidal effects against ***M. javanica*** J2 juveniles of compounds **1**+**2**+**3**, **1**, **4**, **5** and **6a**. Thymol is included as a positive control.

Compound	mg/mL		MLD ^b^	LD_50_^c^	LD_90_^d^
**1**–**3**	0.5	25.83 ± 7.20 ^a^	>0.5		
**1**	0.5	7.11 ± 1.98	>0.5		
**4**	0.5	100.00	0.5	nc	nc
**5**	0.5	0.28 ± 0.12	>0.5		
**6a**	0.5	0.00	>0.5		
**Thymol**	0.5	100	0.25	0.13	0.22
	0.25	99 ± 0.42		(0.13–0.14) ^c^	(0.21–0.23) ^d^
	0.12	32.65 ± 2			
	0.06	19.63 ± 1			

^a^ Corrected according to Scheider–Orelli’s formula, where values are means of four replicates; ^b^ MLD, minimal lethal dose (mg/mL) to give 100% mortality; ^c^ doses (mg/mL) to give 50% and 90% mortality (Probit analysis); ^d^ 95% confidence limits.

## Data Availability

The original contributions presented in this study are included in the article/Supplementary Material. Further inquiries can be directed to the corresponding author.

## Supplementary Materials

The following supporting information can be downloaded at: https://www.mdpi.com/article/10.3390/ijms27072949/s1.
