# Supplementary material for: Isolation and Characterization of Terpenoids with Promising Biopesticide Activity from Dittrichia viscosa (L.) Roots
Source: Int J Mol Sci. 2026 Mar 24;27(7):2949. doi: 10.3390/ijms27072949 (PMC13073822; doi:10.3390/ijms27072949)
Supplement: Supplementary file 1 [file ijms-27-02949-s001.zip › ijms-4157005-supplementary.pdf]

# SUPPORTING INFORMATION

## Isolation and characterization of terpenoids with promising biopesticide activity from Dittrichia viscosa (L.) roots

*María José Segura Navarro, José Francisco Quílez del Moral, Alberto Galisteo, José Luis López-Pérez, María Fé Andrés, Azucena González-Coloma, Alejandro Fernández Barrero*

## Table of contents


Figure S1. $^{1}H$ NMR spectrum (400 MHz, $CDCl_3$) of compound 1. ........ 4
Figure S2. $^{13}C$ NMR spectrum (101 MHz, $CDCl_3$) of compound 1. ........ 4
Figure S3. HSQC spectrum (400/101 MHz, $CDCl_3$) of compound 1. ........ 5
Figure S4. HMBC spectrum (500/126 MHz, $CDCl_3$) of compound 1. ........ 5
Figure S5. 1D TOCSY spectrum (400 MHz, $CDCl_3$) of compound 1. ........ 6
Figure S6. $^{1}H$ NMR spectrum (400 MHz, $CDCl_3$) of compound 2. ........ 7
Figure S7. $^{13}C$ NMR spectrum (101 MHz, $CDCl_3$) of compound 2. ........ 7
Figure S8. HSQC spectrum (400/101 MHz, $CDCl_3$) of compound 2. ........ 8
Figure S9. $^{1}H$ NMR spectrum (400 MHz, $CDCl_3$) of compound 3. ........ 8
Figure S10. $^{13}C$ NMR spectrum (101 MHz, $CDCl_3$) of compound 3. ........ 9
Figure S11. HSQC spectrum (400/101 MHz, $CDCl_3$) of compound 3. ........ 9
Figure S12. $^{1}H$ NMR spectrum (400 MHz, $CDCl_3$) of compound 4. ........ 10
Figure S13. $^{13}C$ NMR spectrum (101 MHz, $CDCl_3$) of compound 4. ........ 10
Figure S14. $^{1}H$ NMR spectrum (500 MHz, CDCl3) of compound 5. ........ 11
Figure S15. $^{13}C$ NMR spectrum (126 MHz, $CDCl_3$) of compound 5. ........ 11
Figure S16. HSQC spectrum (500/126 MHz, $CDCl_3$) of compound 5. ........ 12
Figure S17. HMBC spectrum (500/126 MHz, CDCl3) of compound 5. ........ 12
Figure S18. 1D TOCSY spectrum (500 MHz, CDCl3) of compound 5. ........ 13
Figure S19. 1D TOCSY spectrum (500 MHz, $CDCl_3$) of compound 5. ........ 13
Figure S20. 1D NOE spectrum (500 MHz, $CDCl_3$) of compound 5. ........ 14
Figure S21. 1D NOE spectrum (500 MHz, $CDCl_3$) of compound 5. ........ 14
Figure S22. $^{1}H$ NMR spectrum (500 MHz, $CDCl_3$) of compound 6. ........ 15
Figure S23. $^{13}C$ NMR spectrum (126 MHz, $CDCl_3$) of compound 6. ........ 15
Figure S24. HSQC spectrum (500/126 MHz, $CDCl_3$) of compound 6. ........ 16
Figure S25. HMBC spectrum (500/126 MHz, CDCl3) of compound 6. ........ 16
Figure S26. 1D TOCSY spectrum (600 MHz, $CDCl_3$) of compound 6. ........ 17
Figure S27. 1D NOE spectrum (600 MHz, $CDCl_3$) of compound 6. ........ 17
Figure S28. 1D NOE spectrum (600 MHz, $CDCl_3$) of compound 6. ........ 18
Figure S29. $^{1}H$ NMR spectrum (500 MHz, $CDCl_3$) of compound 6a. ........ 18
Figure S30. $^{13}C$ NMR spectrum (126 MHz, $CDCl_3$) of compound 6a. ........ 19
Figure S31. HSQC spectrum (500/126 MHz, $CDCl_3$) of compound 6a. ........ 19
Figure S32. HMBC spectrum (500/126 MHz, $CDCl_3$) of compound 6a. ........ 20
Figure S33. 1D TOCSY spectrum (500 MHz, $CDCl_3$) of compound 6a. ........ 20
Figure S34. 1D TOCSY spectrum (500 MHz, $CDCl_3$) of compound 6a. ........ 21
Figure S35. 1D NOE spectrum (500 MHz, $CDCl_3$) of compound 6a. ........ 21

Figure S36. 1D NOE spectrum (500 MHz, $CDCl_3$) of compound 6a. ........................................... 22
Figure S37. $^1$H NMR spectrum (500 MHz, $CDCl_3$) of compound 7. ........................................... 22
Figure S38. $^1$H NMR spectrum (500 MHz, $CDCl_3$) of compound 8. ........................................... 23
Figure S39. $^{13}$C NMR spectrum (126 MHz, $CDCl_3$) of compound 8. ........................................... 23
Figure S40. HSQC spectrum (500/126 MHz, $CDCl_3$) of compound 8. ........................................... 24
Figure S41. HMBC spectrum (500/126 MHz, $CDCl_3$) of compound 8. ........................................... 24
Figure S42. 1D TOCSY spectrum (500 MHz, $CDCl_3$) of compound 8. ........................................... 25
Figure S43. 1D TOCSY spectrum (500 MHz, $CDCl_3$) of compound 8. ........................................... 25
Figure S44. 1D NOE spectrum (500 MHz, $CDCl_3$) of compound 8. ........................................... 26
Figure S45. 1D NOE spectrum (500 MHz, $CDCl_3$) of compound 8. ........................................... 26
Table S1. Cartesian coordinates (mol2) of the conformer with the lowest energy out of all possible conformers of 5. ........................................... 27
Table S2. Comparation between the theorical $^{13}$C RMN of the lower energy conformer of 5 and the corresponding experimental values. ........................................... 28
Optical Rotation calculation of 5. ........................................... 29
Table S3. Lower energy conformations of 6. ........................................... 31
Table S4. Lower energy conformations of 6 epimer at C-7. ........................................... 32
Table S5. Cartesian coordinates (mol2) of the global minimum conformer (M01) of 6. ........................................... 33
Table S6. Cartesian coordinates (mol2) of the conformer M02 of 6. ........................................... 35
Table S7. Cartesian coordinates (mol2) of the conformer M04 of 6. ........................................... 37
Table S8. Cartesian coordinates (mol2) of the global minimum conformer M01 of 6 epimer at C-7. ........................................... 39
Table S9. Cartesian coordinates (mol2) of the conformer M14 of conformer M01 of 6 epimer at C-7. ........................................... 41

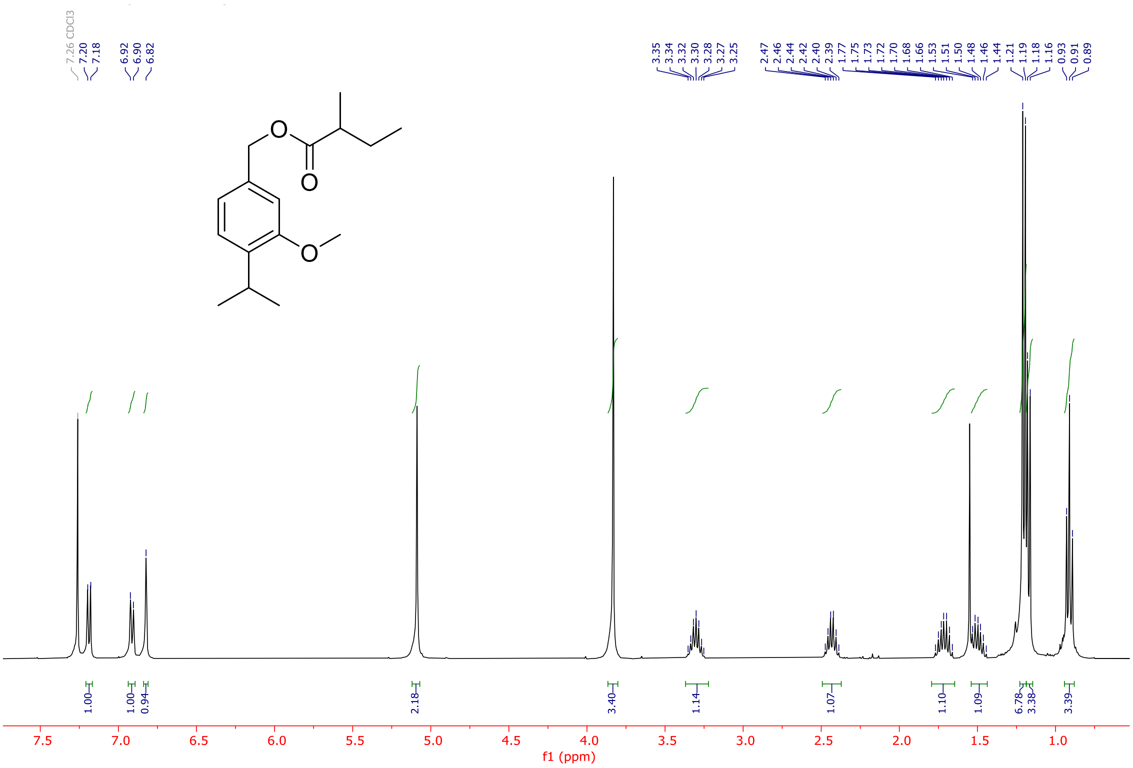

**Figure S1.** $^1H$ NMR spectrum (400 MHz, $CDCl_3$) of compound **1**.

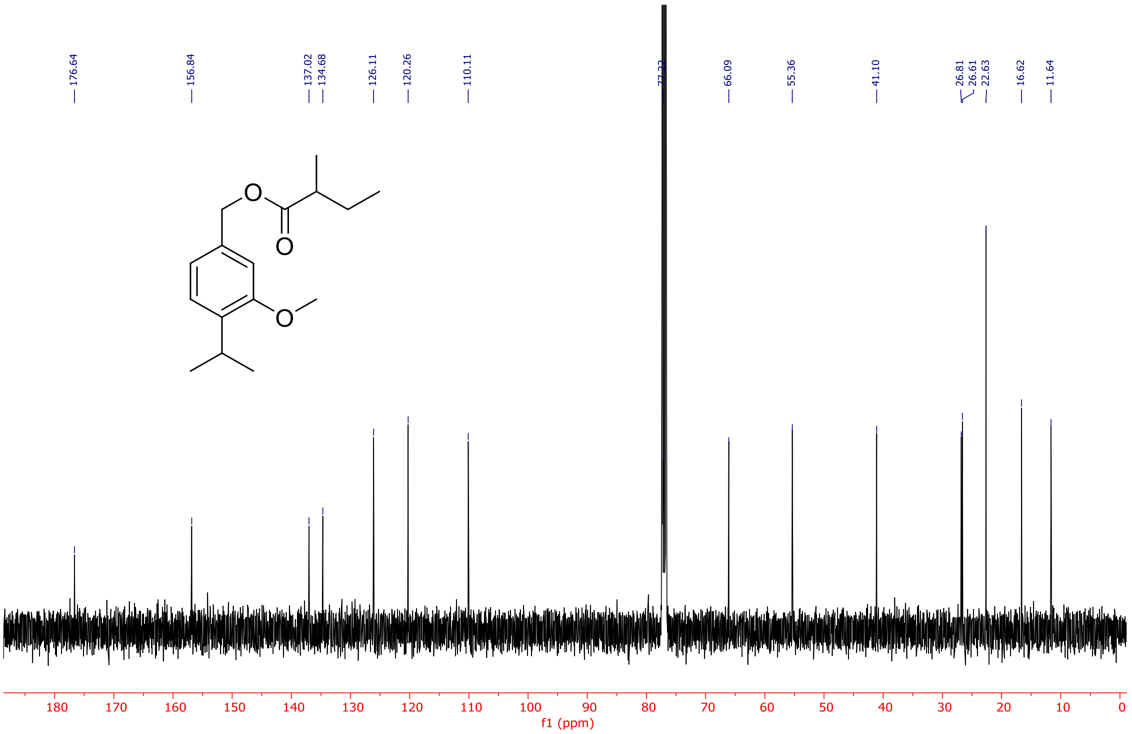

**Figure S2.** $^{13}C$ NMR spectrum (101 MHz, $CDCl_3$) of compound **1**.

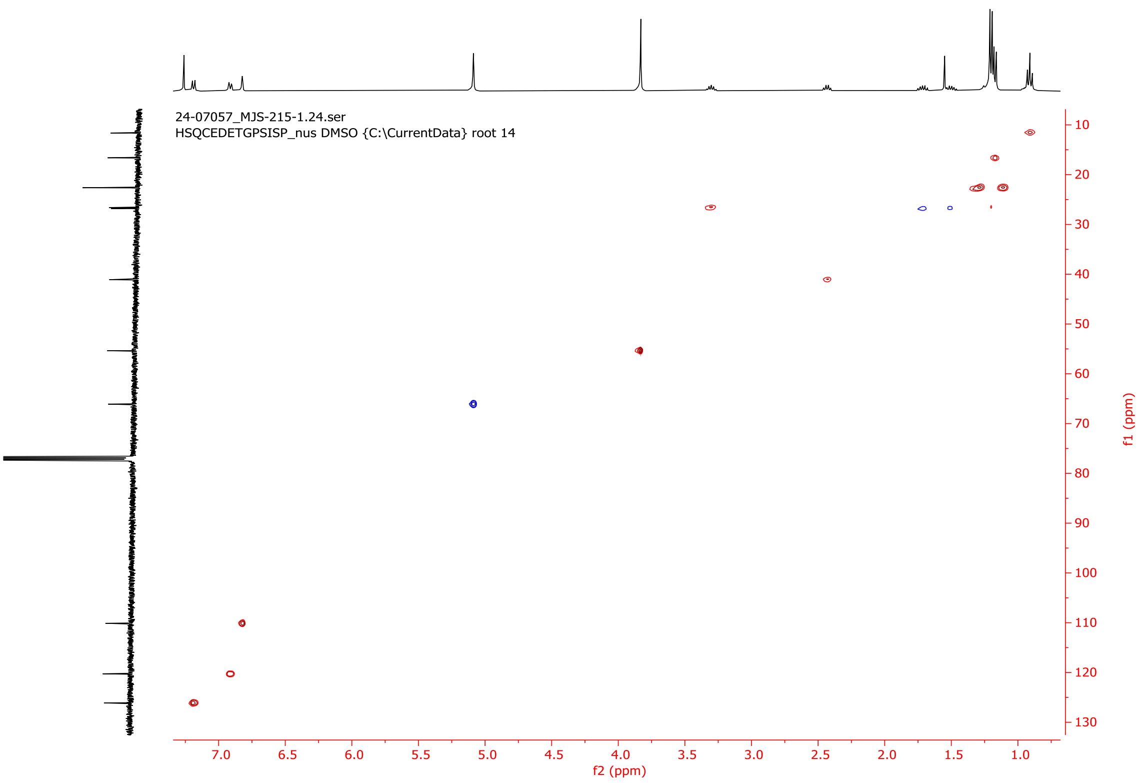


**Figure S3.** HSQC spectrum (400/101 MHz, $CDCl_3$) of compound **1**.

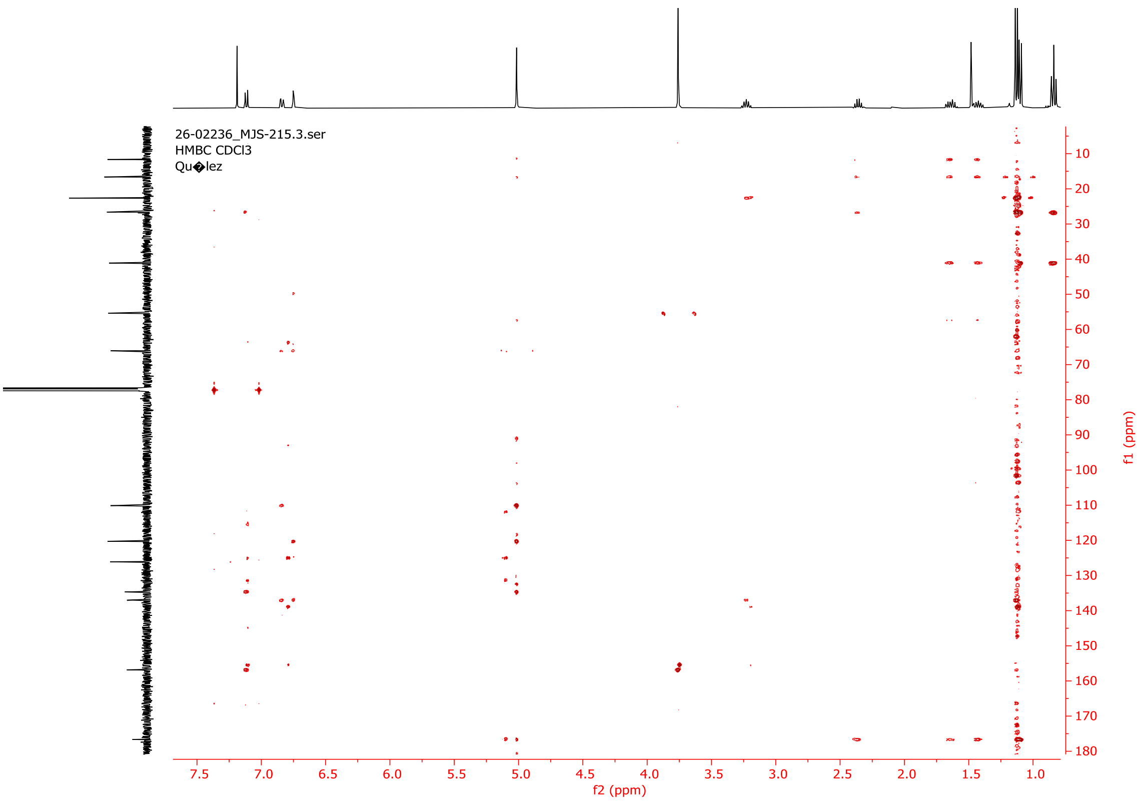


**Figure S4**. HMBC spectrum (500/126 MHz, $CDCl_3$) of compound **1**.

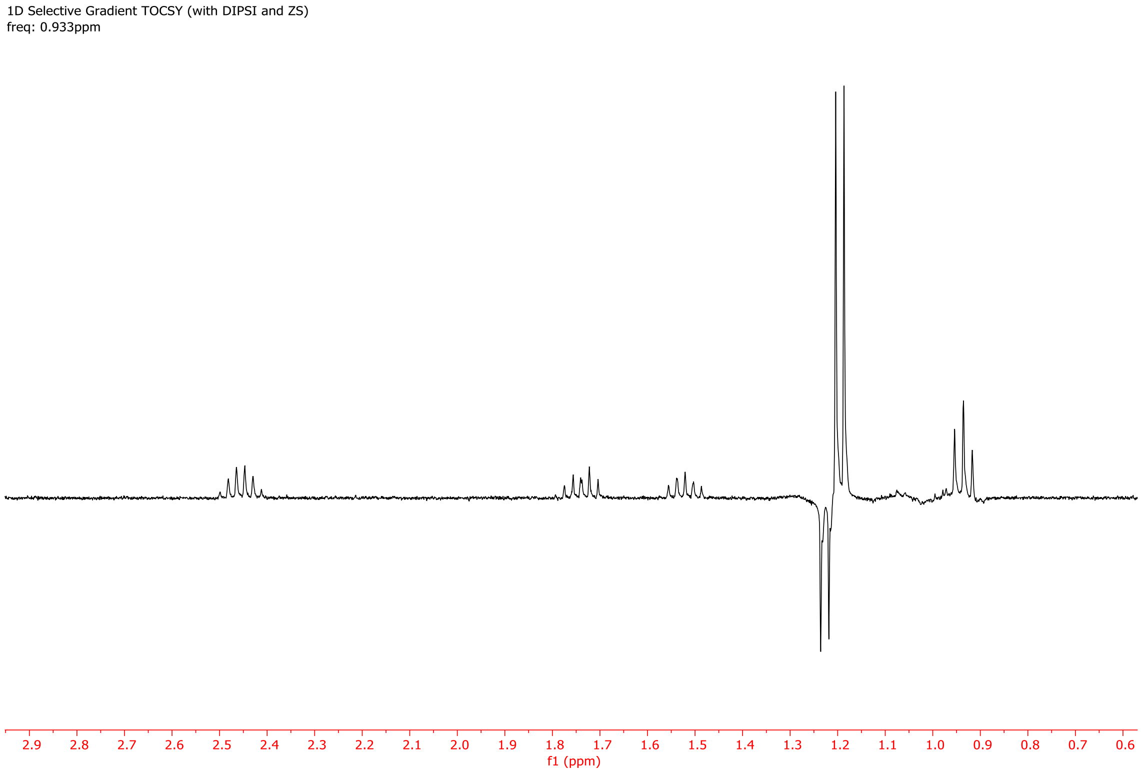


**Figure S5.** 1D TOCSY spectrum (400 MHz, $CDCl_3$) of compound **1**.

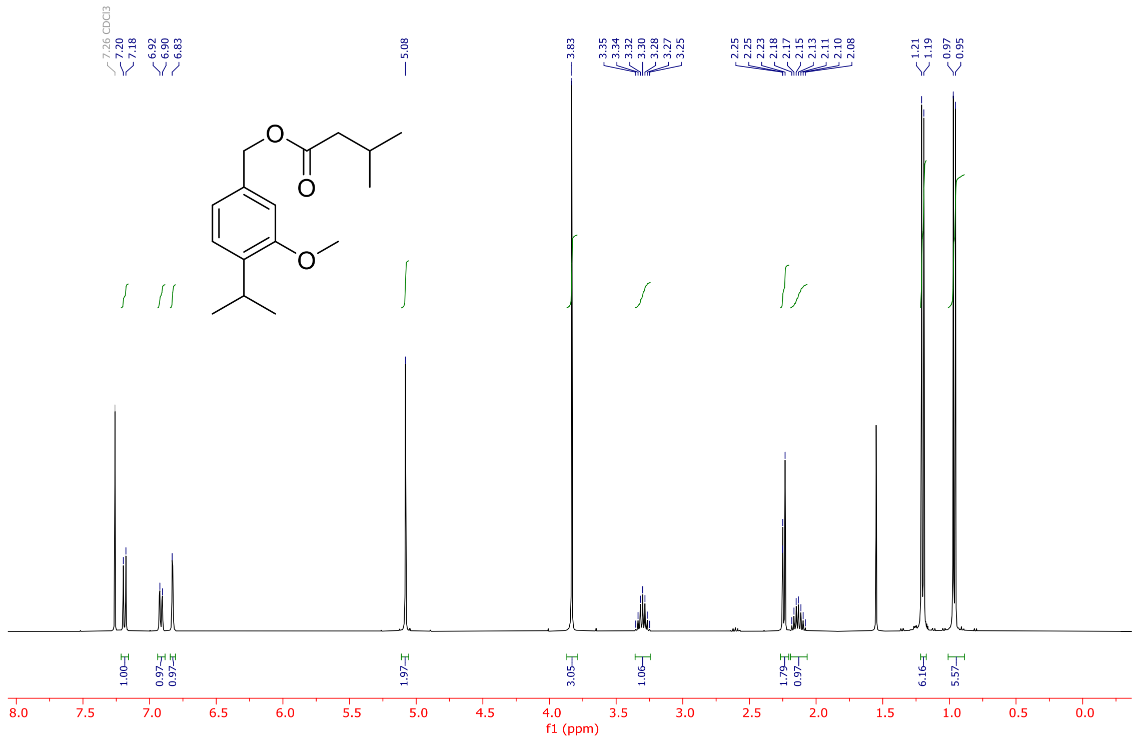


**Figure S6.** $^{1}$H NMR spectrum (400 MHz, $CDCl_3$) of compound **2**.

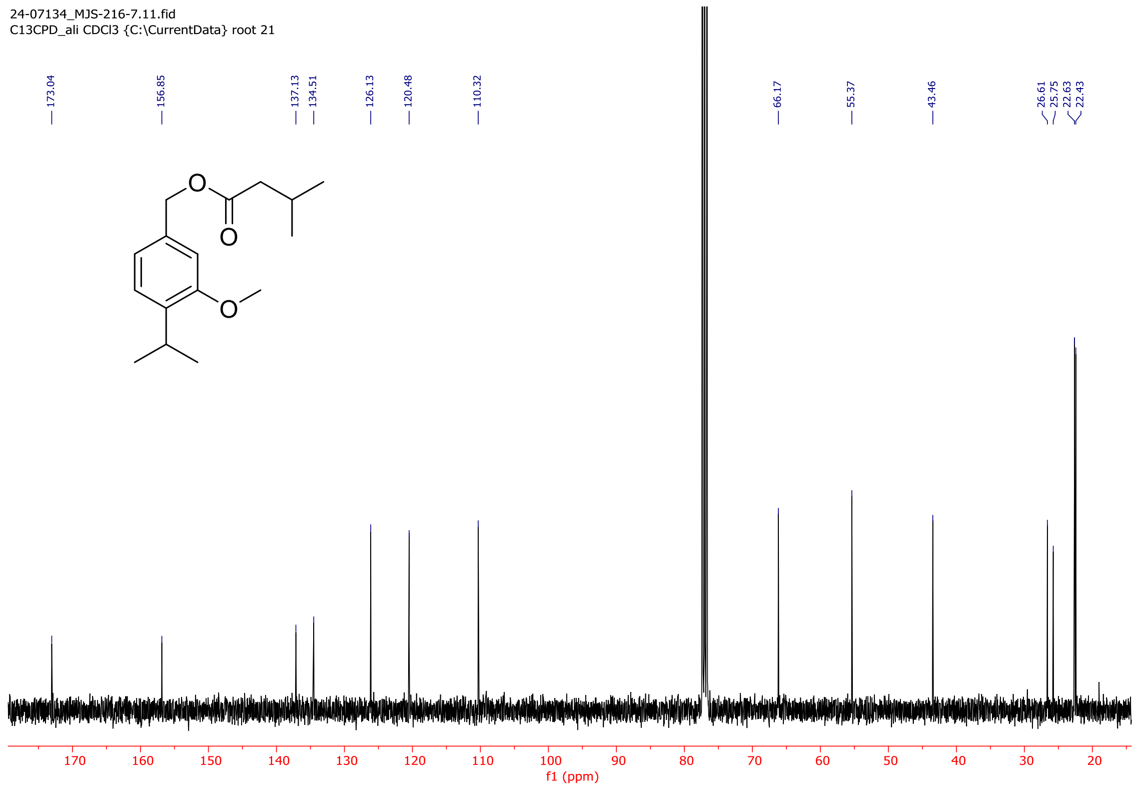


**Figure S7**. $^{13}$C NMR spectrum (101 MHz, $CDCl_3$) of compound **2**.

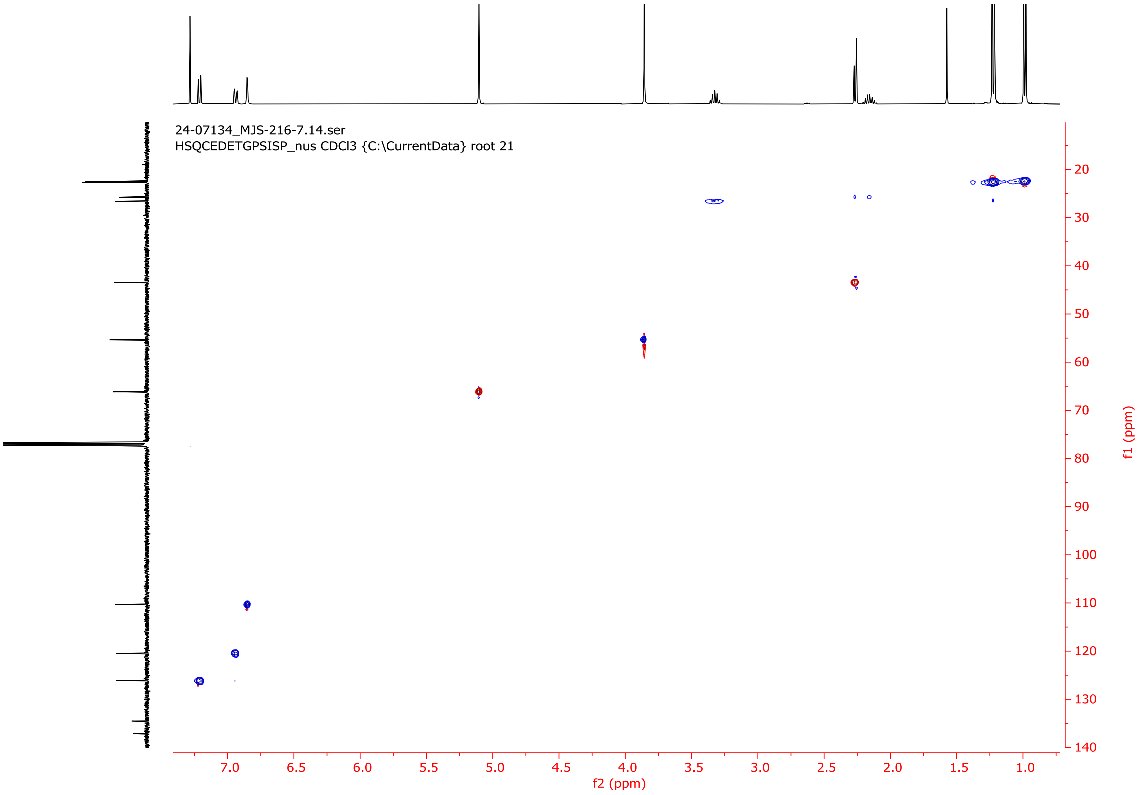


**Figure S8.** HSQC spectrum (400/101 MHz, $CDCl_3$) of compound **2**.

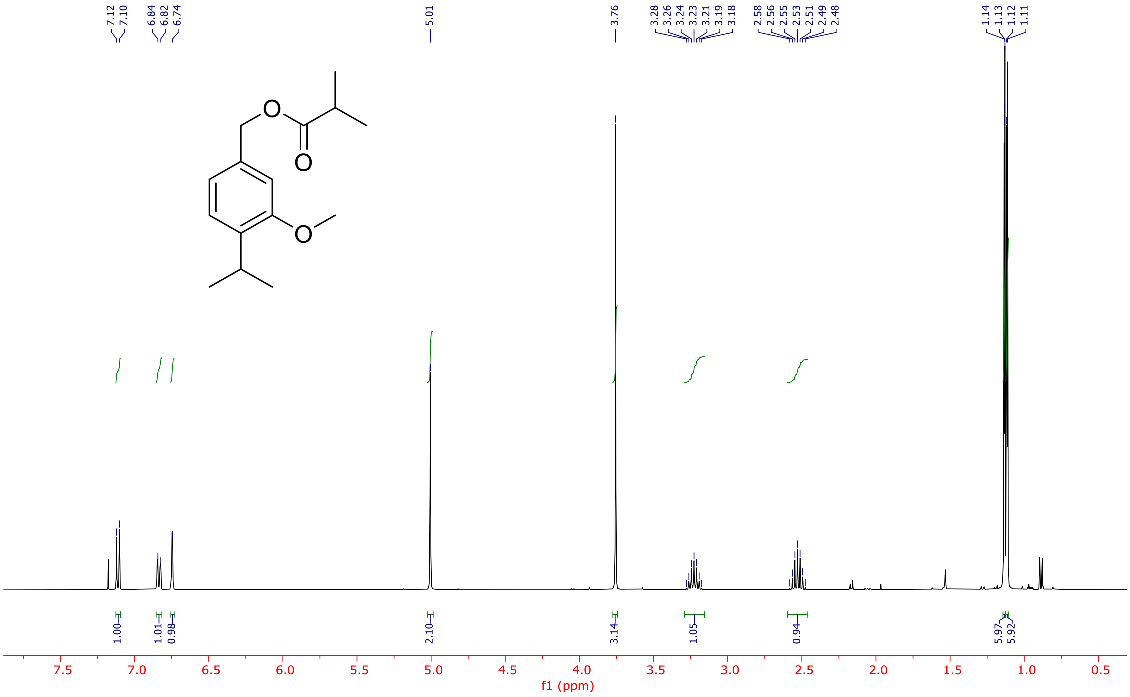


**Figure S9.** $^1$H NMR spectrum (400 MHz, $CDCl_3$) of compound **3**.

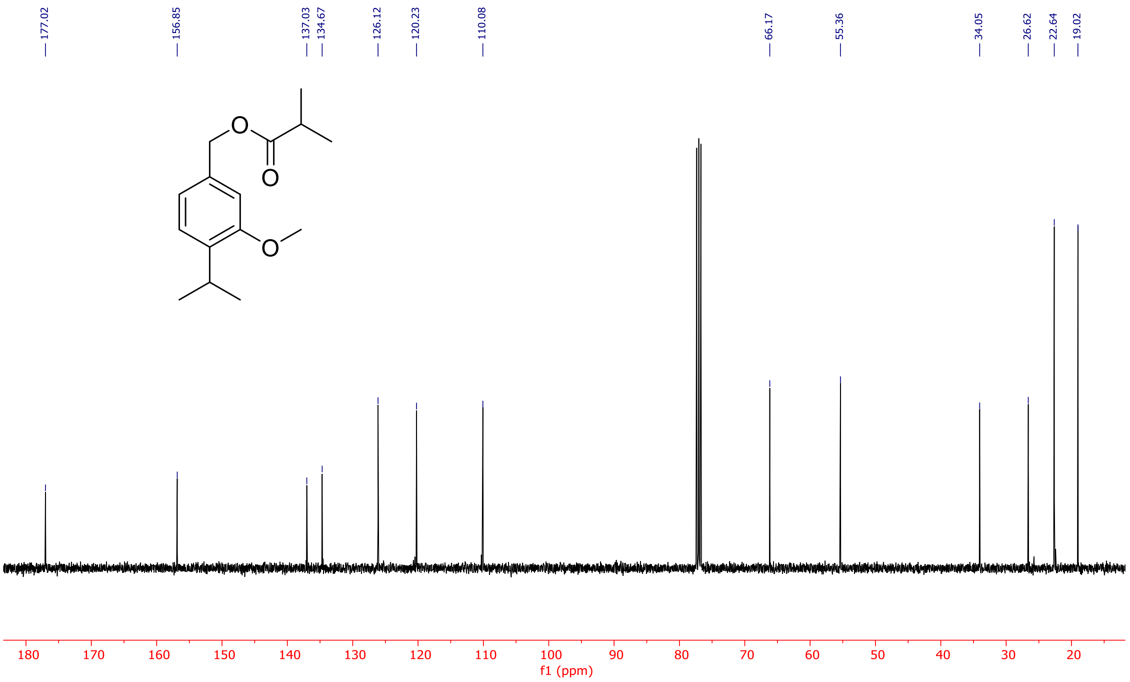


**Figure S10.** $^{13}C$ NMR spectrum (101 MHz, $CDCl_3$) of compound **3**.

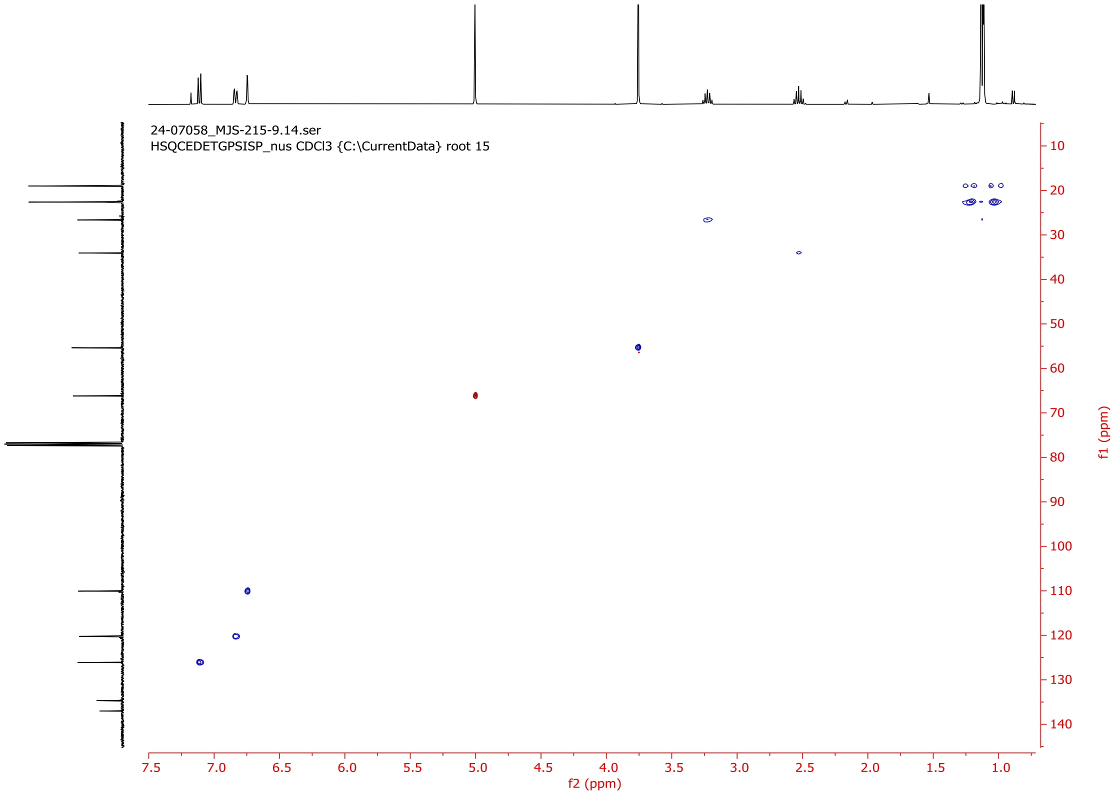


**Figure S11.** HSQC spectrum (400/101 MHz, $CDCl_3$) of compound **3**.

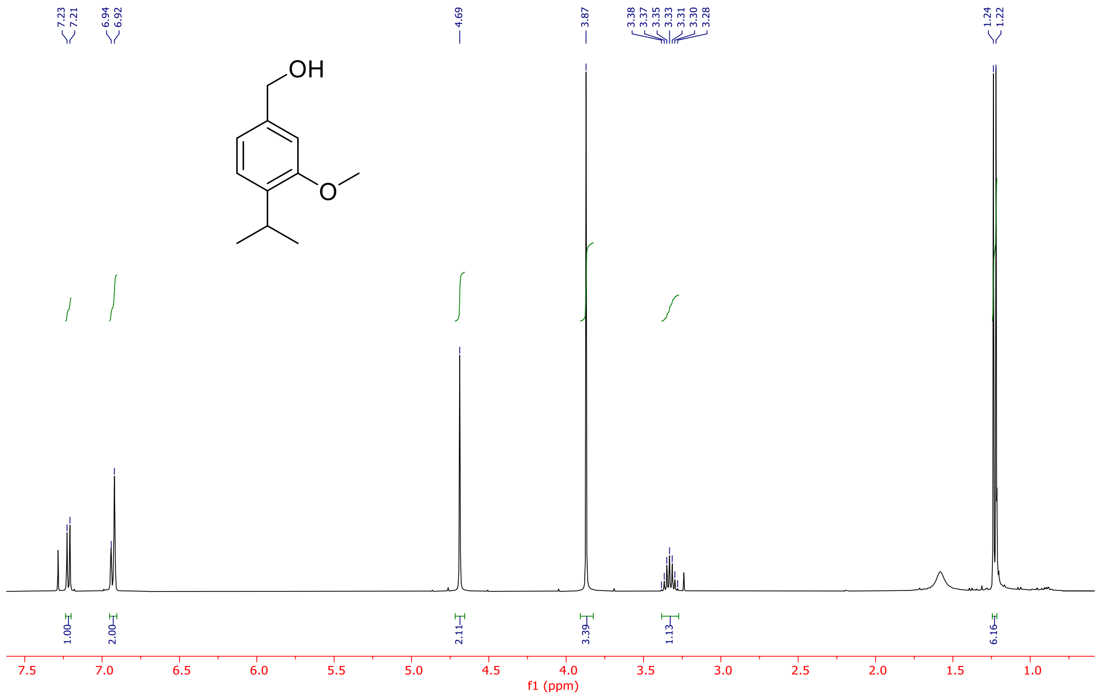

**Figure S12.** $^{1}$H NMR spectrum (400 MHz, $CDCl_3$) of compound **4**.

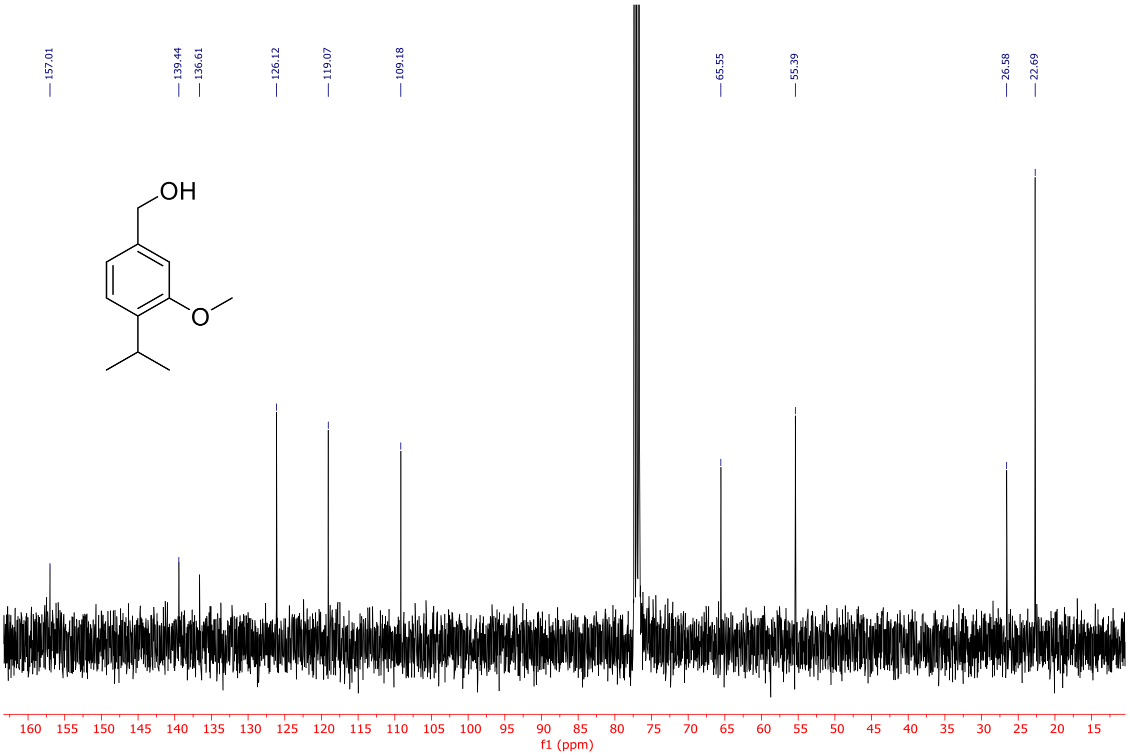

**Figure S13.** $^{13}$C NMR spectrum (101 MHz, $CDCl_3$) of compound **4**.

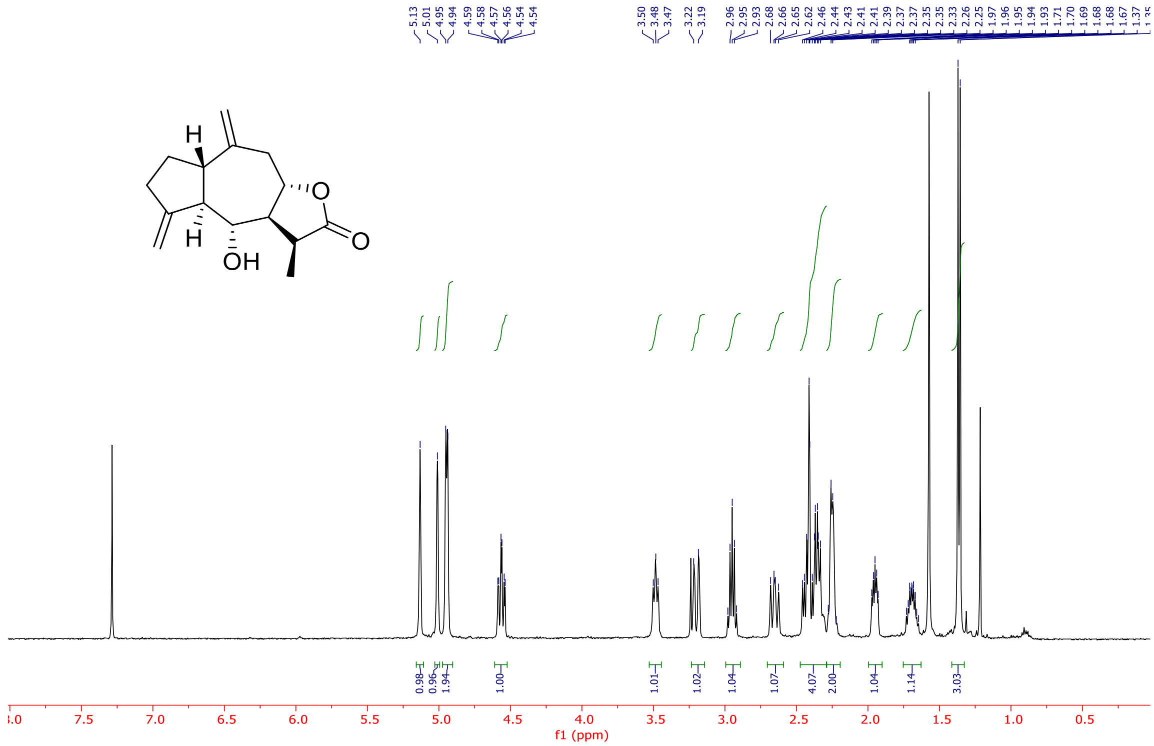

**Figure S14.** $^{1}$H NMR spectrum (500 MHz, CDCl3) of compound **5**.

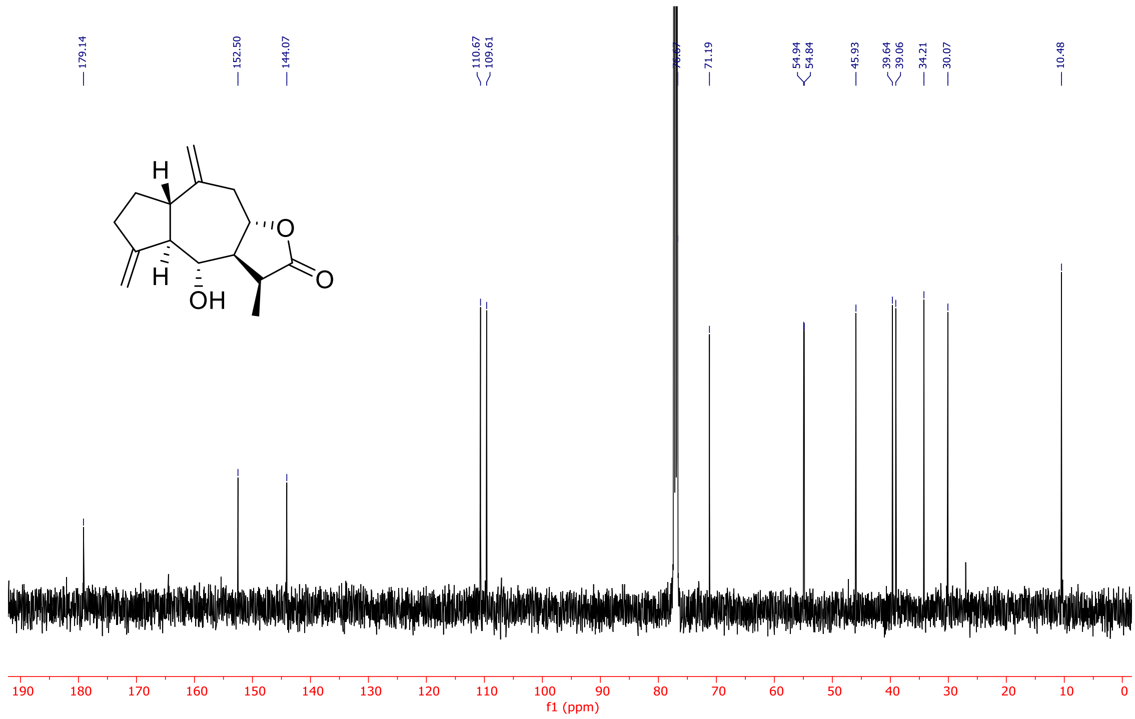

**Figure S15.** $^{13}$C NMR spectrum (126 MHz, $CDCl_3$) of compound **5**.

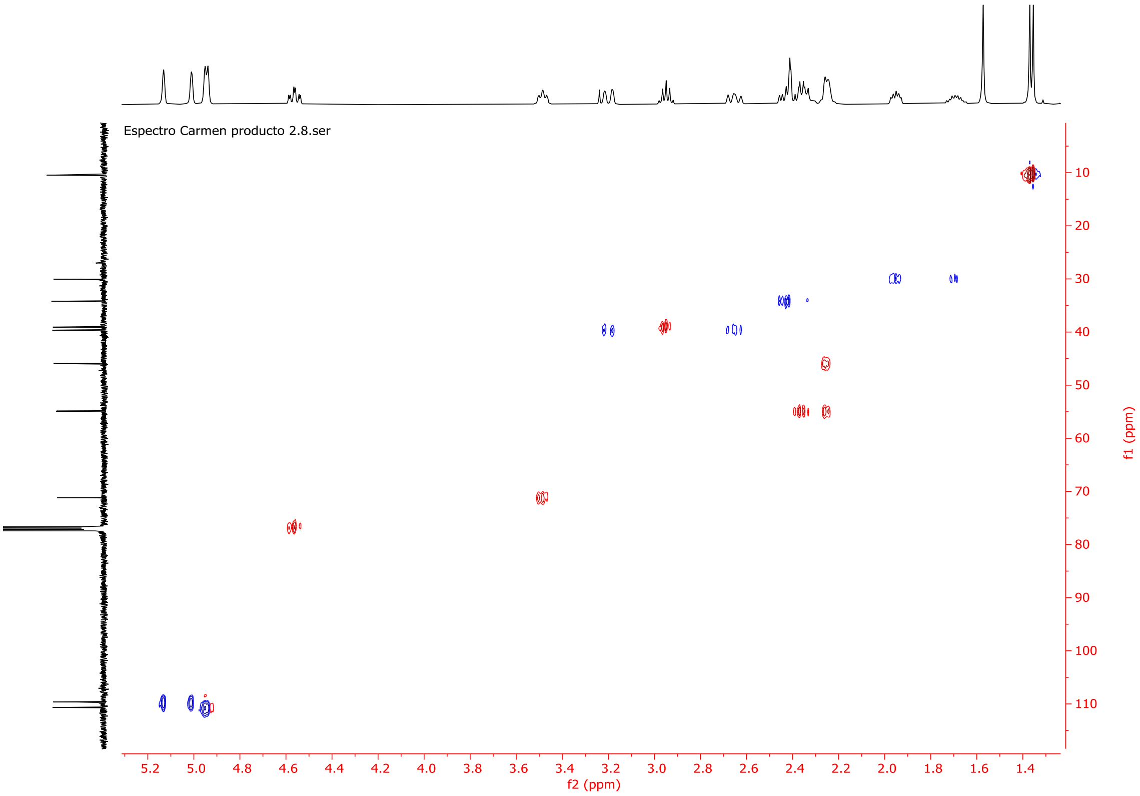


**Figure S16.** HSQC spectrum (500/126 MHz, $CDCl_3$) of compound **5**.

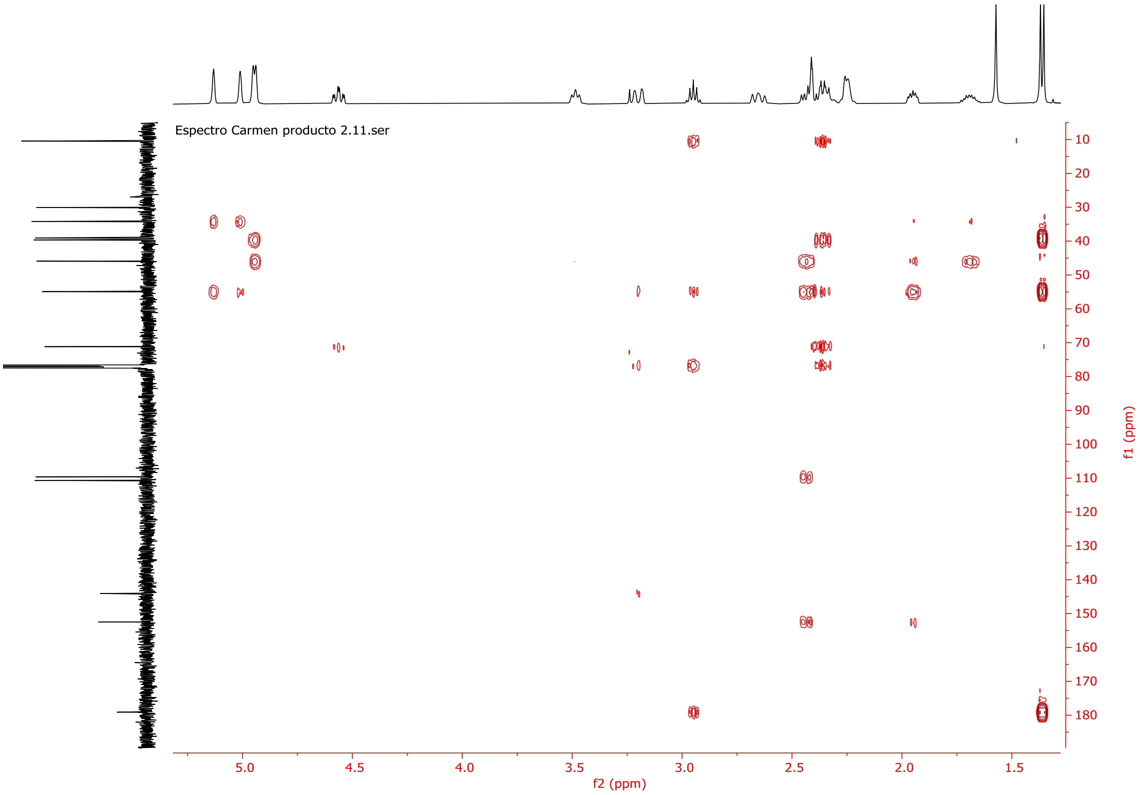


**Figure S17.** HMBC spectrum (500/126 MHz, CDCl3) of compound **5**.

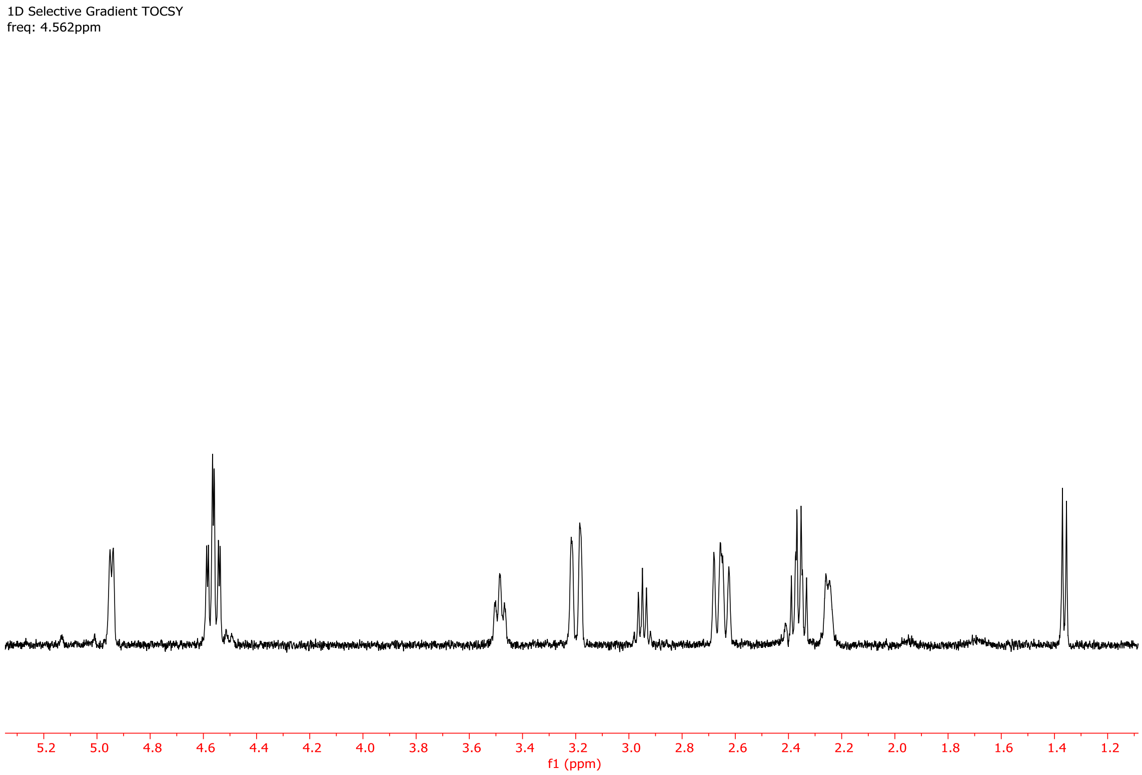


**Figure S18.** 1D TOCSY spectrum (500 MHz, CDCl3) of compound **5**.

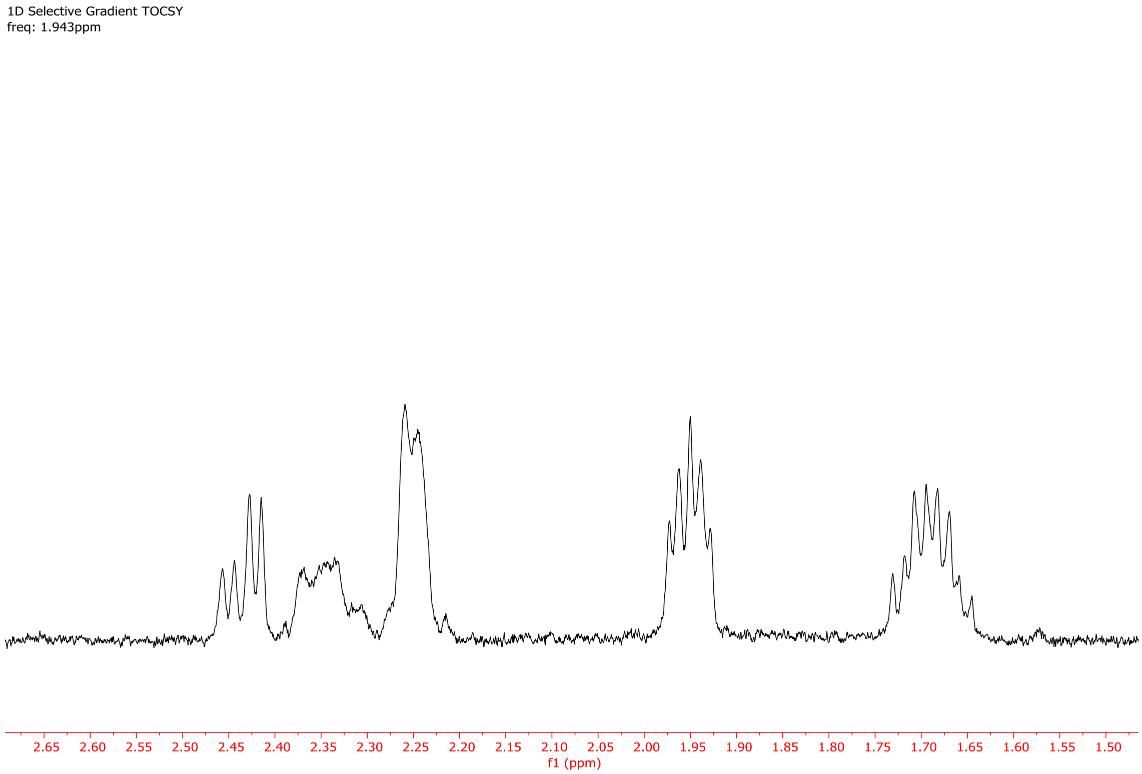


**Figure S19.** 1D TOCSY spectrum (500 MHz, $CDCl_3$) of compound **5**.

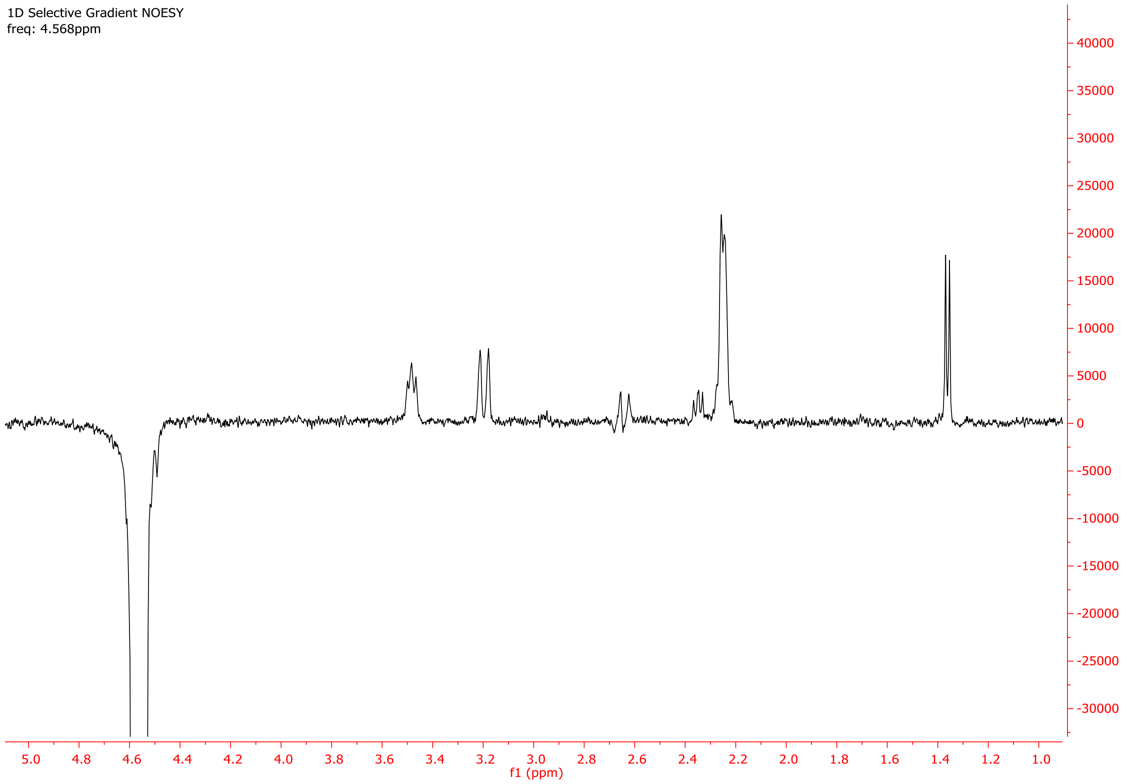


**Figure S20.** 1D NOE spectrum (500 MHz, $CDCl_3$) of compound **5**.

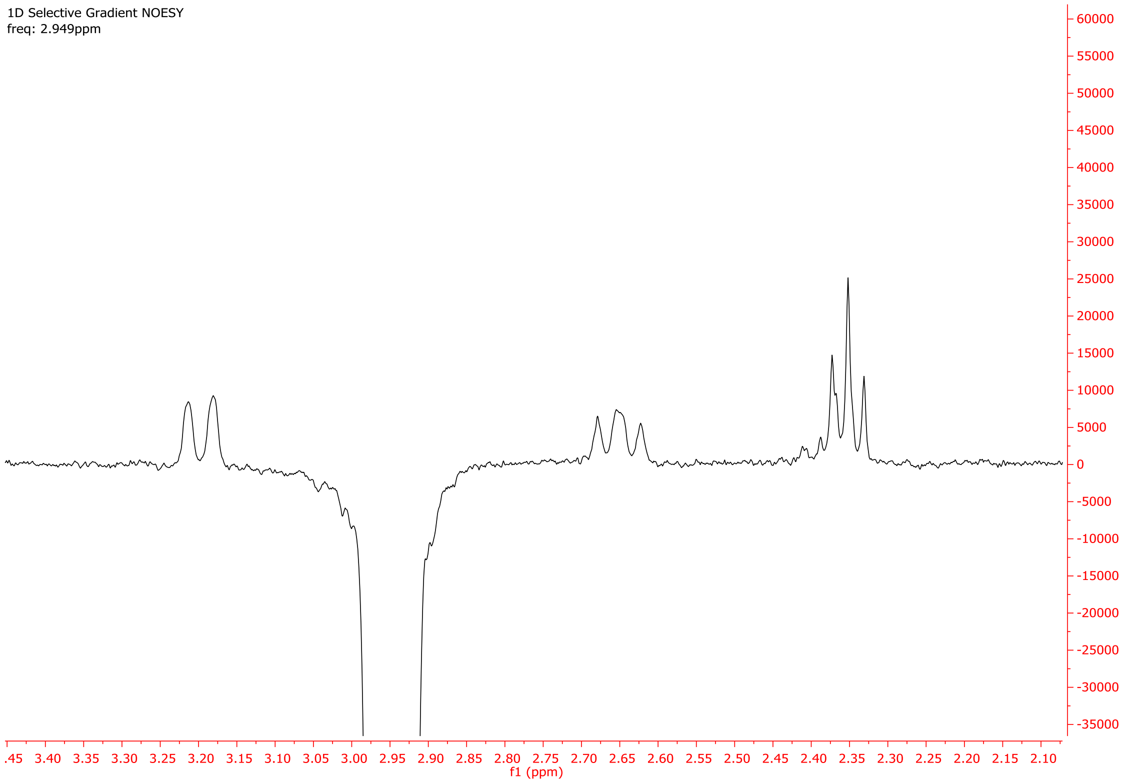


**Figure S21.** 1D NOE spectrum (500 MHz, $CDCl_3$) of compound **5**.

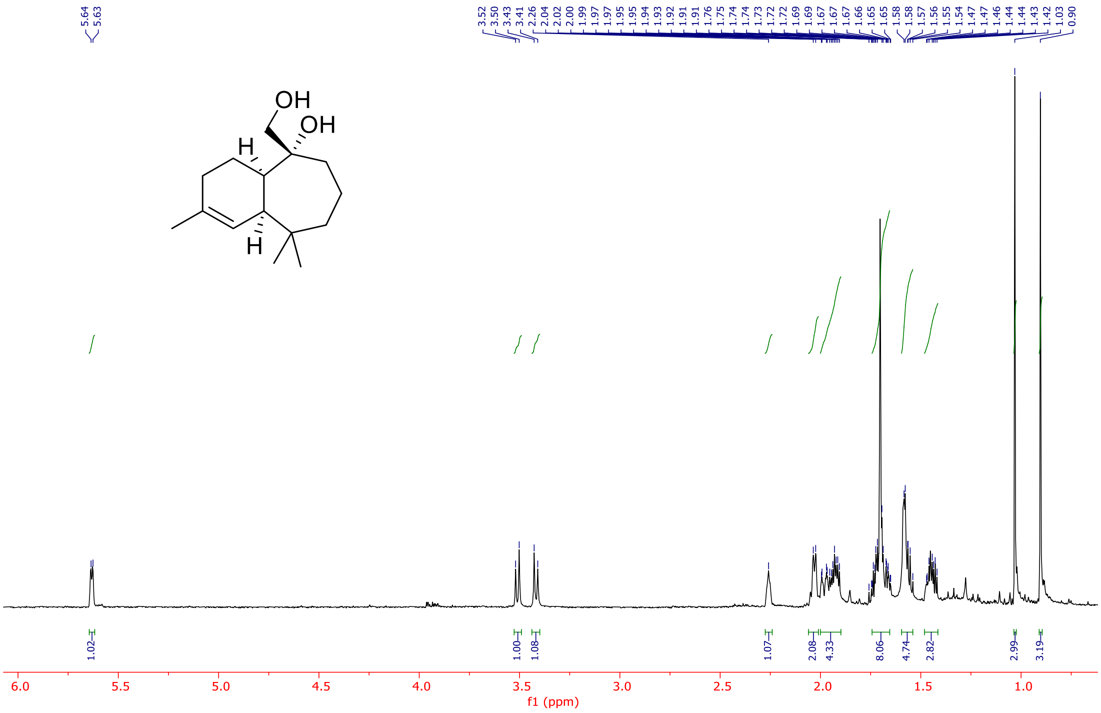

**Figure S22.** $^{1}$H NMR spectrum (500 MHz, $CDCl_3$) of compound **6**.

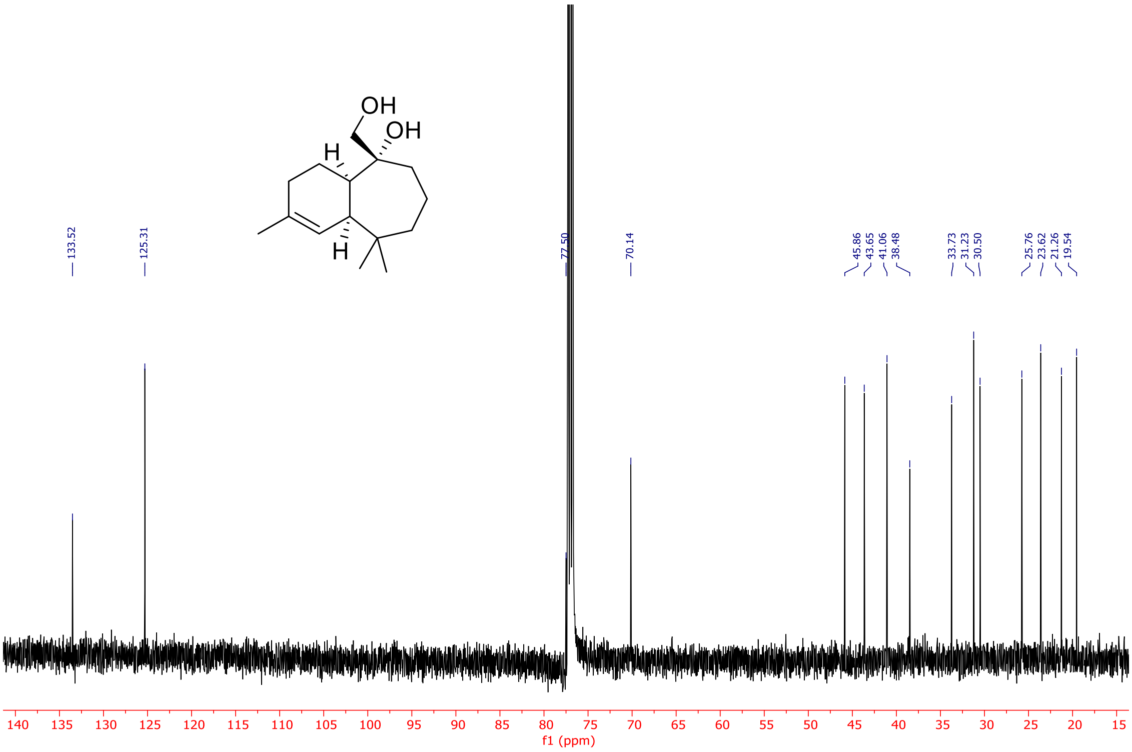

**Figure S23.** $^{13}$C NMR spectrum (126 MHz, $CDCl_3$) of compound **6**.

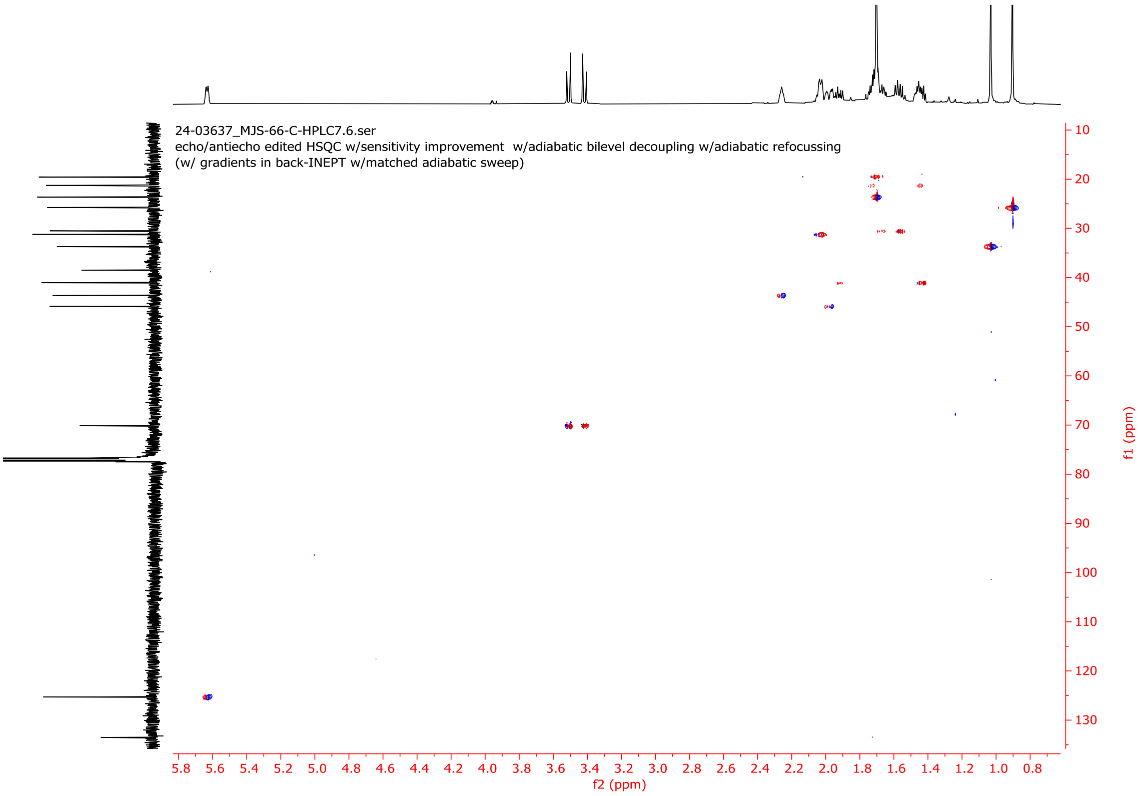


**Figure S24.** HSQC spectrum (500/126 MHz, $CDCl_3$) of compound **6**.

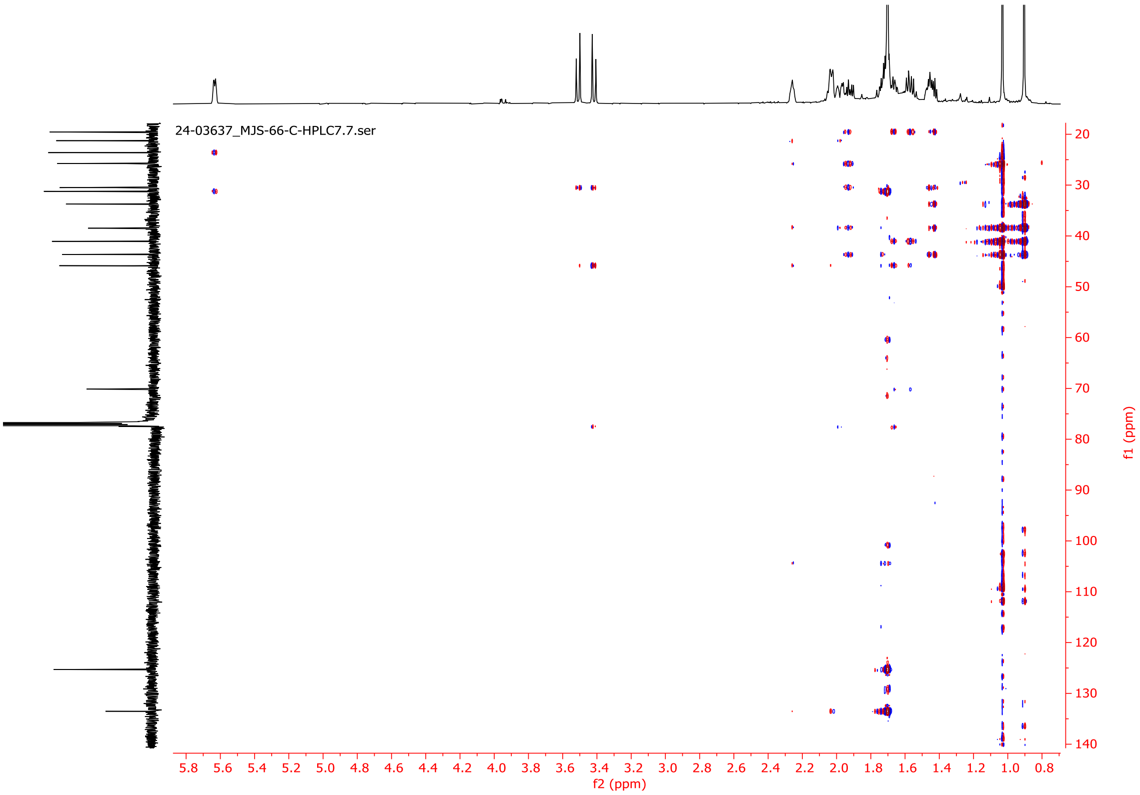


**Figure S25.** HMBC spectrum (500/126 MHz, CDCl3) of compound **6**.

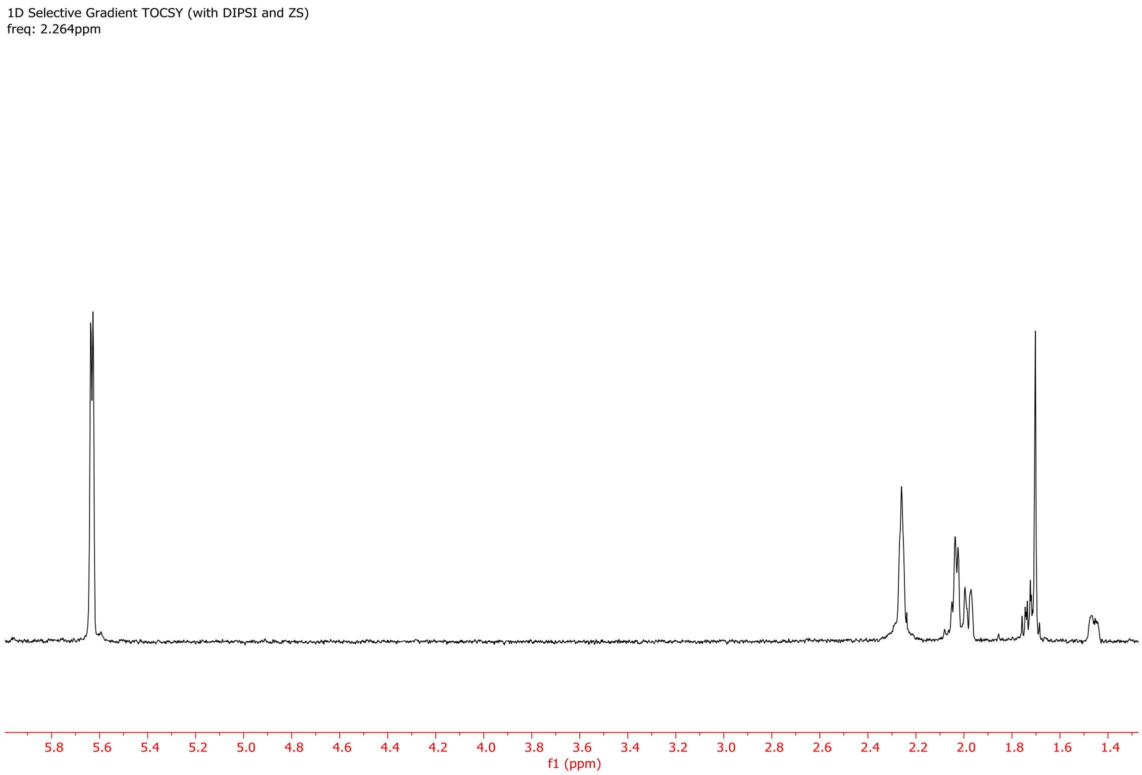


**Figure S26.** 1D TOCSY spectrum (600 MHz, $CDCl_3$) of compound **6**.

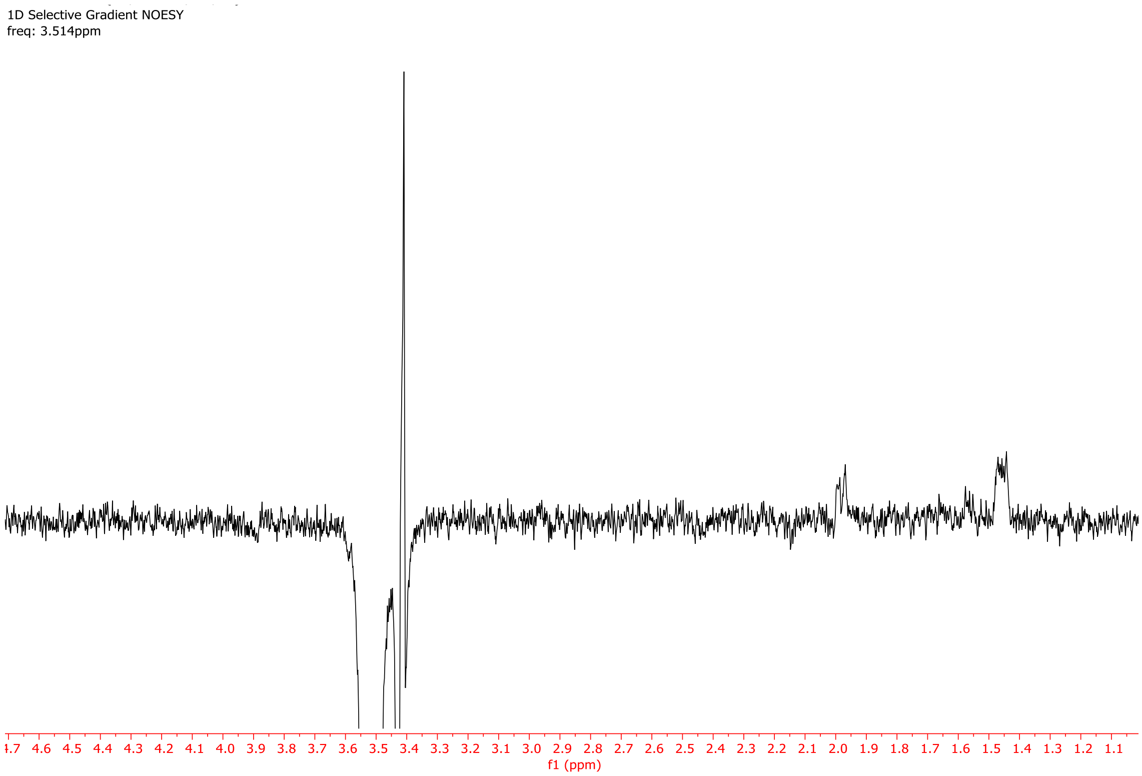


**Figure S27.** 1D NOE spectrum (600 MHz, $CDCl_3$) of compound **6**.

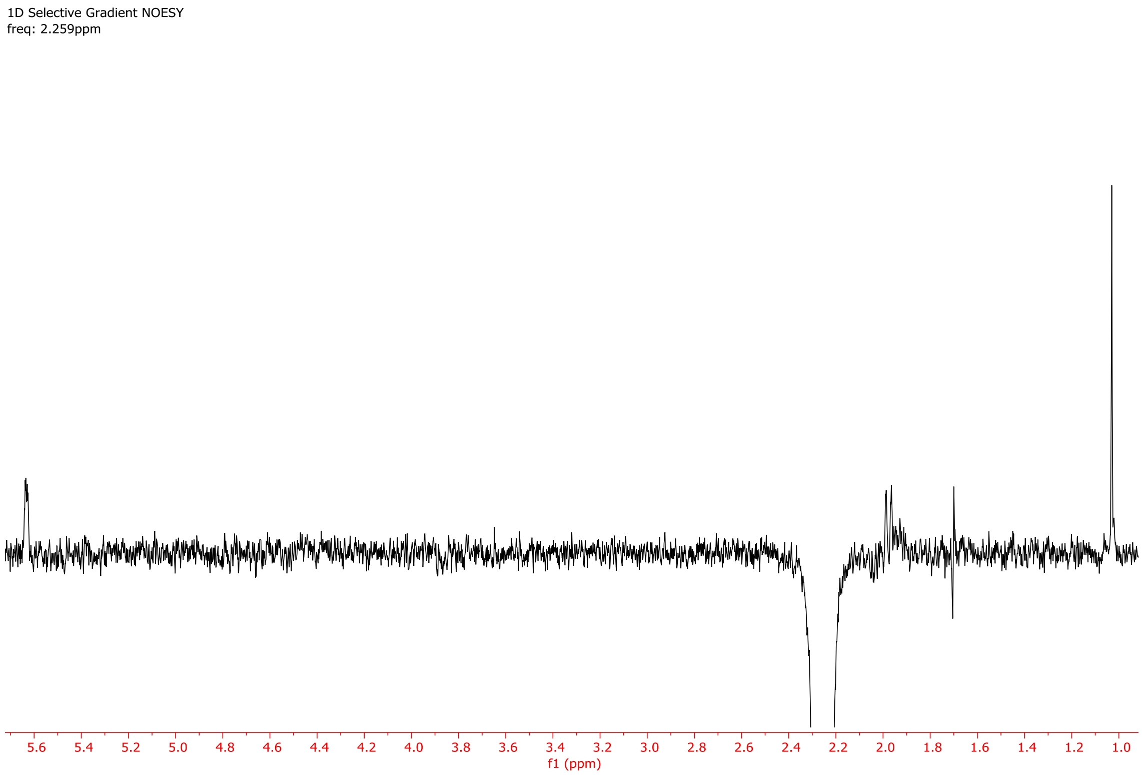


**Figure S28.** 1D NOE spectrum (600 MHz, $CDCl_3$) of compound **6**.

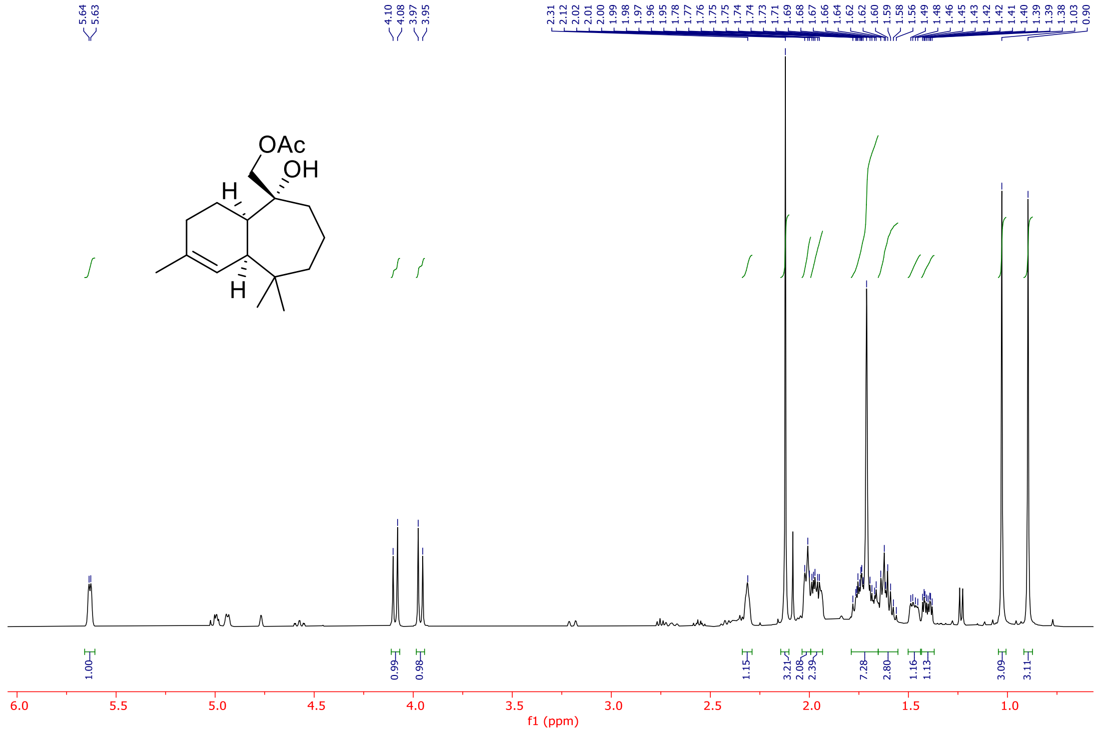


**Figure S29.** $^1H$ NMR spectrum (500 MHz, $CDCl_3$) of compound **6a**.

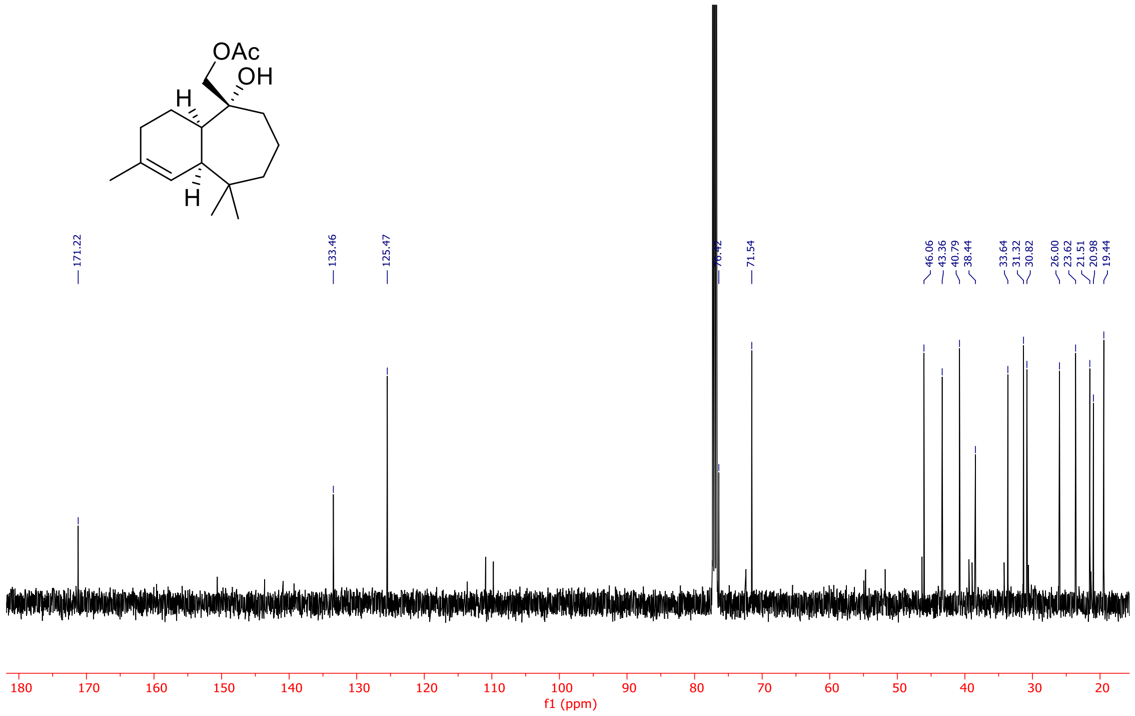


**Figure S30.** $^{13}C$ NMR spectrum (126 MHz, $CDCl_3$) of compound **6a**.

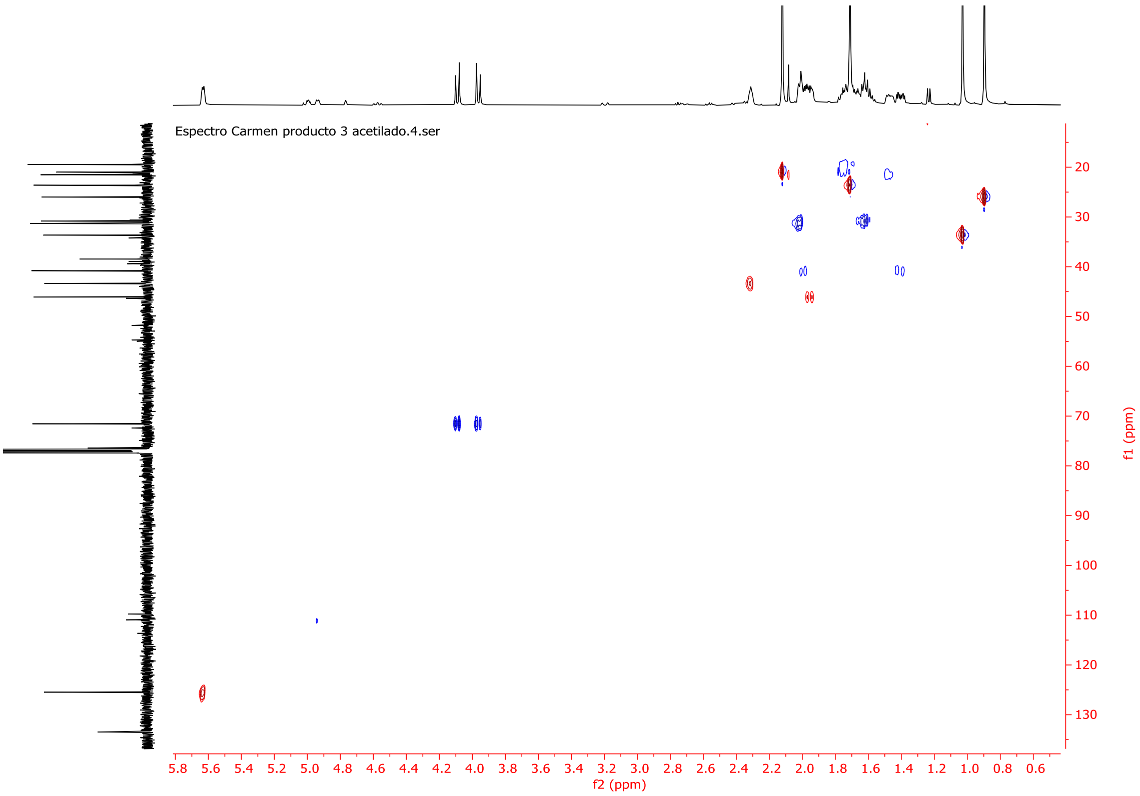


**Figure S31.** HSQC spectrum (500/126 MHz, $CDCl_3$) of compound **6a**.

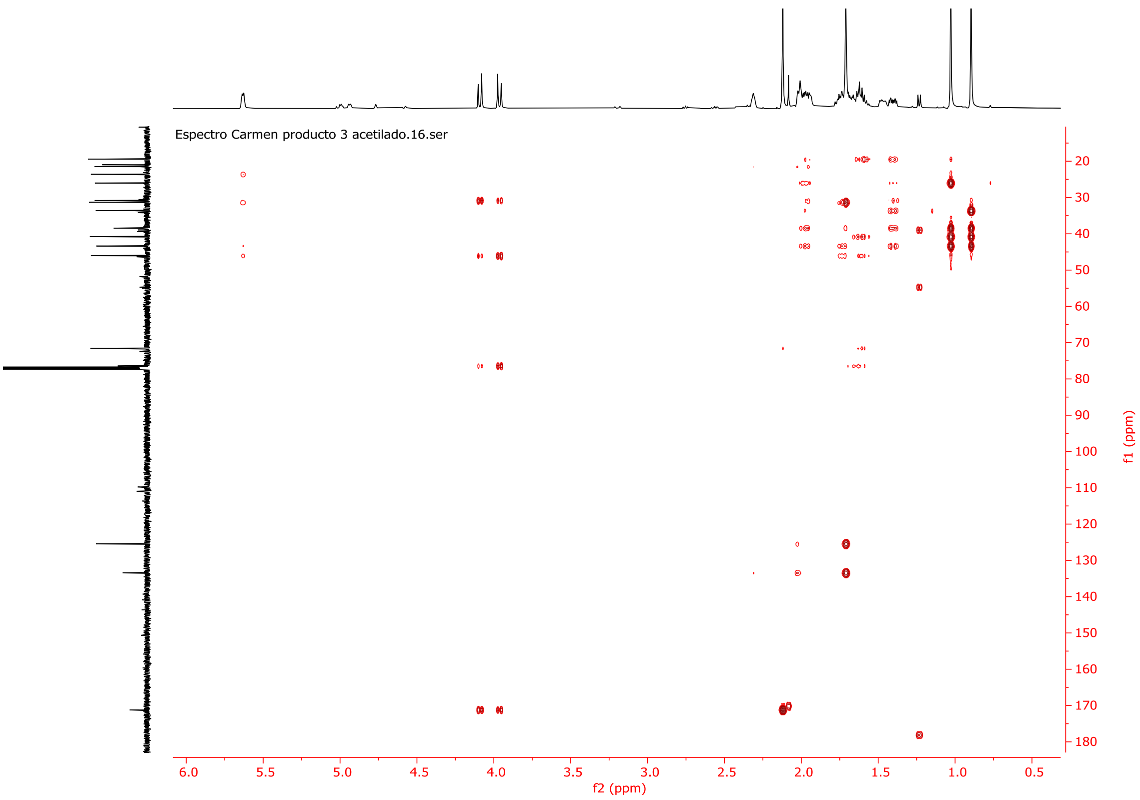


**Figure S32.** HMBC spectrum (500/126 MHz, $CDCl_3$) of compound **6a**.

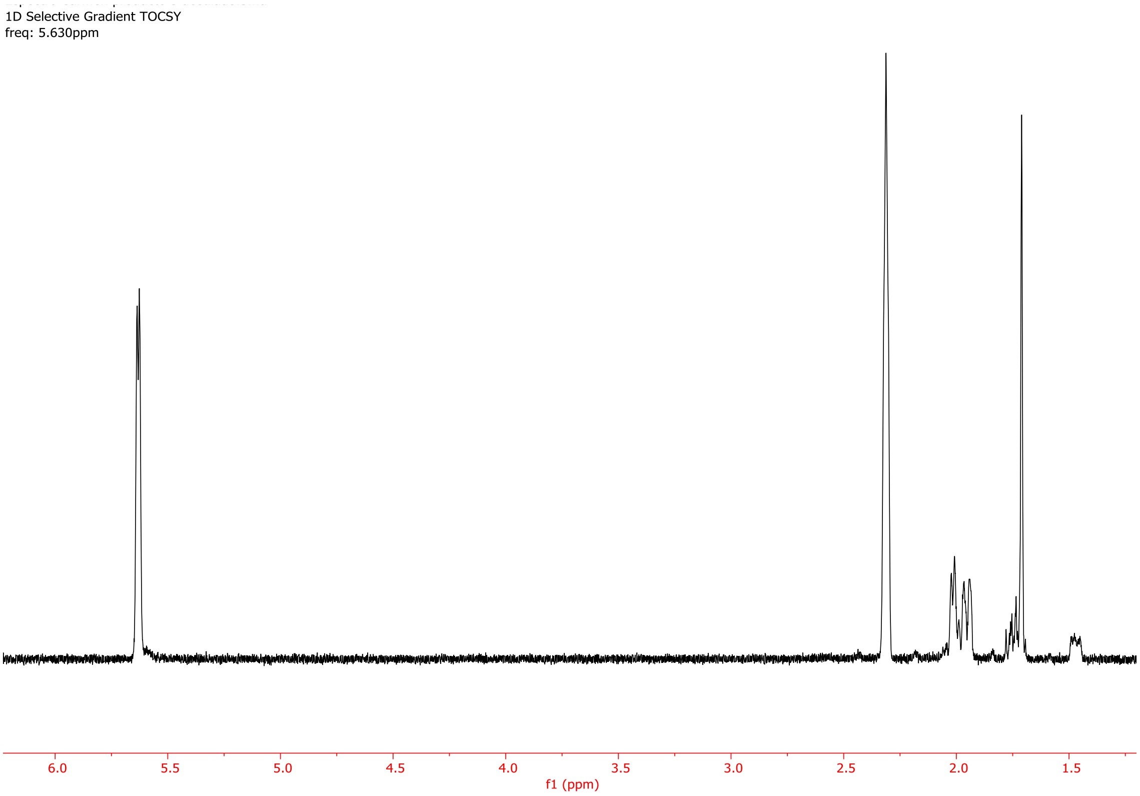


**Figure S33.** 1D TOCSY spectrum (500 MHz, $CDCl_3$) of compound **6a**.

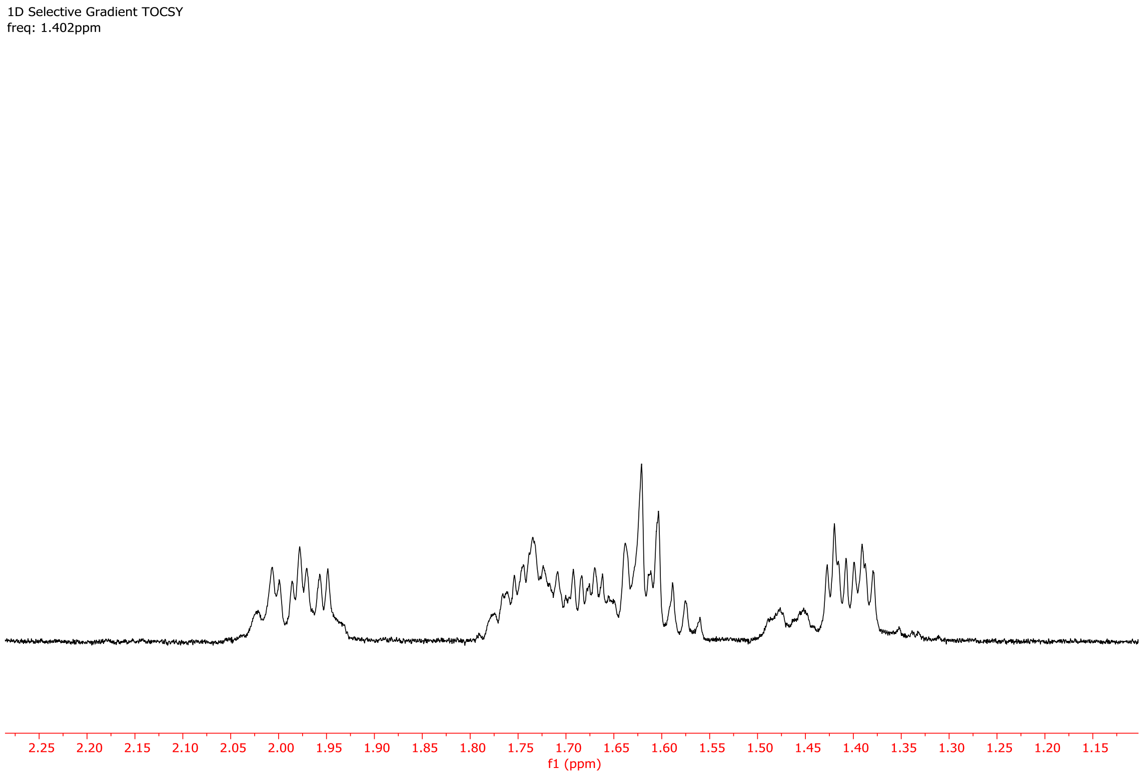


**Figure S34.** 1D TOCSY spectrum (500 MHz, $CDCl_3$) of compound **6a**.

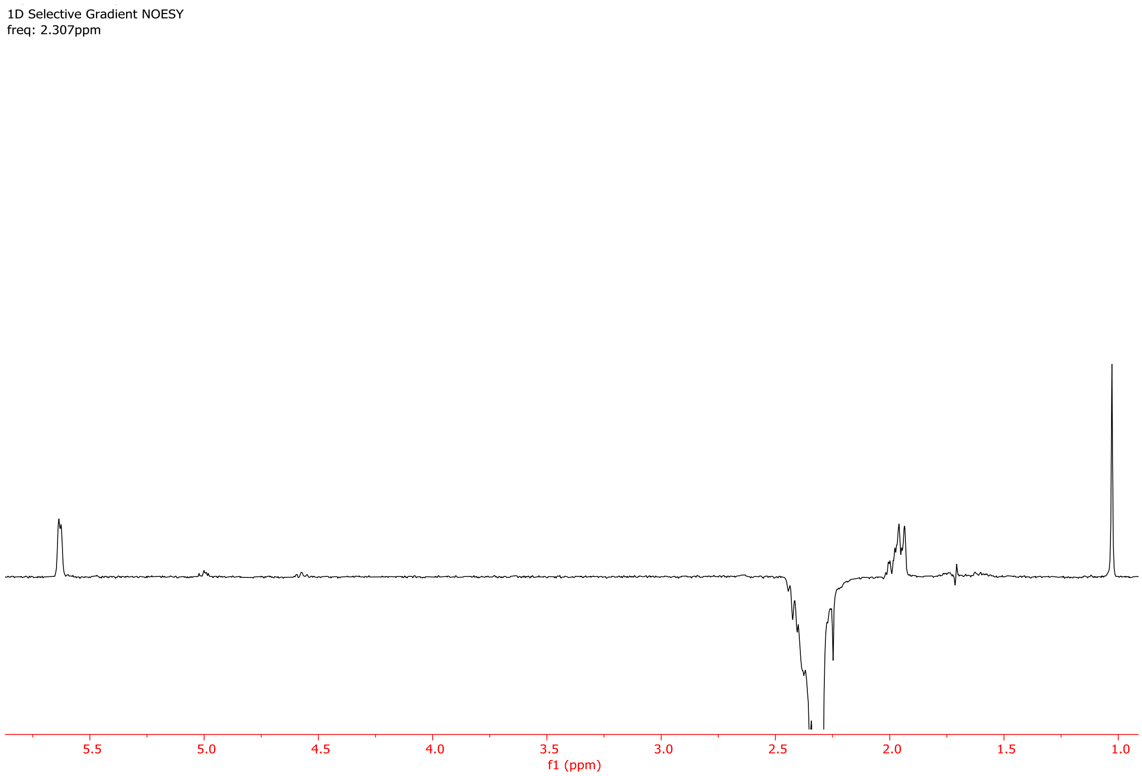


**Figure S35.** 1D NOE spectrum (500 MHz, $CDCl_3$) of compound **6a**.

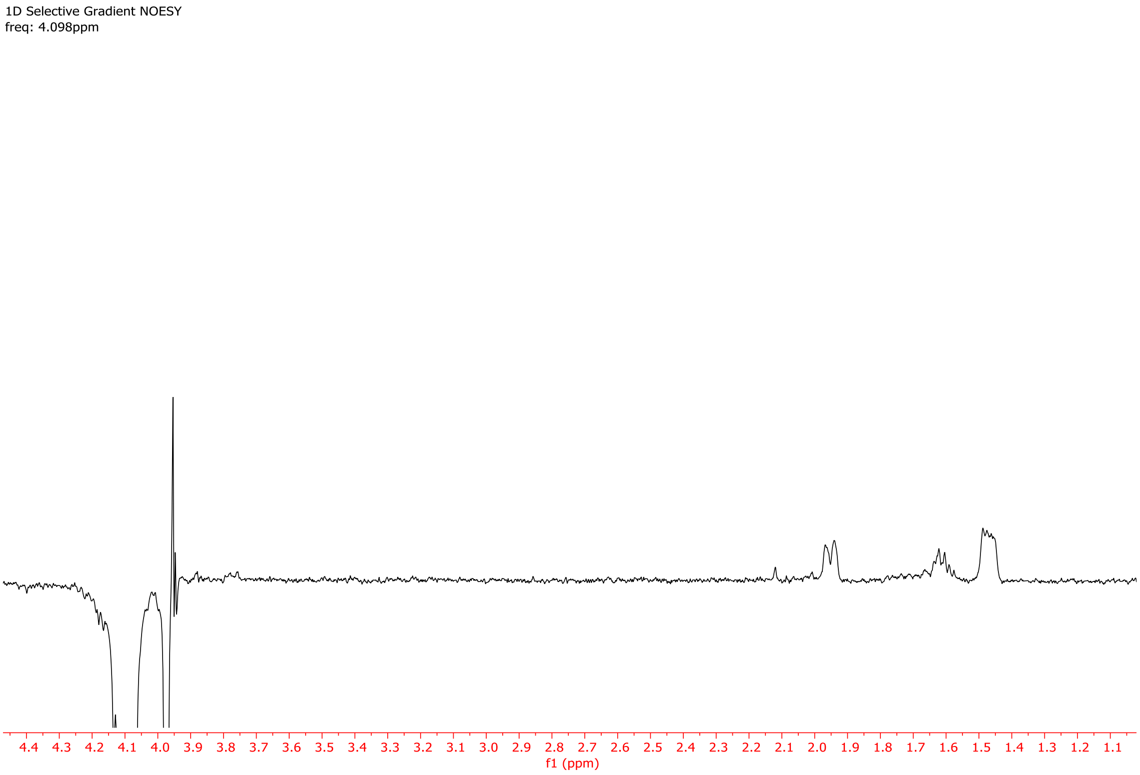


**Figure S36.** 1D NOE spectrum (500 MHz, $CDCl_3$) of compound **6a**.

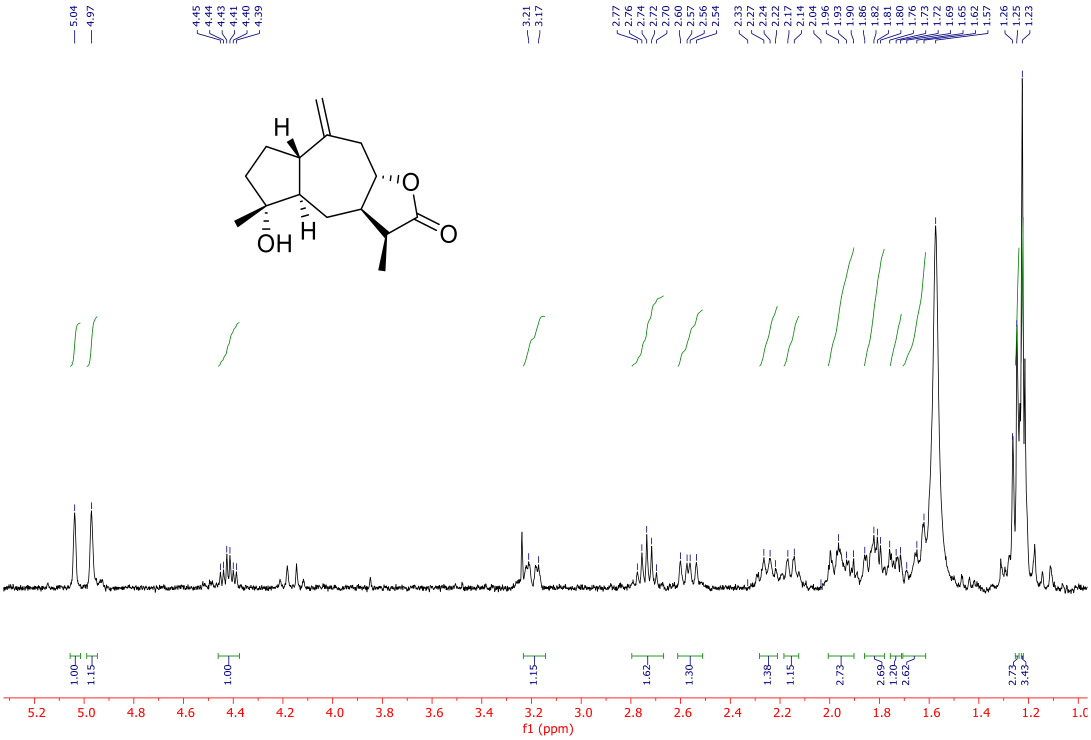


**Figure S37.** $^1H$ NMR spectrum (500 MHz, $CDCl_3$) of compound **7**.

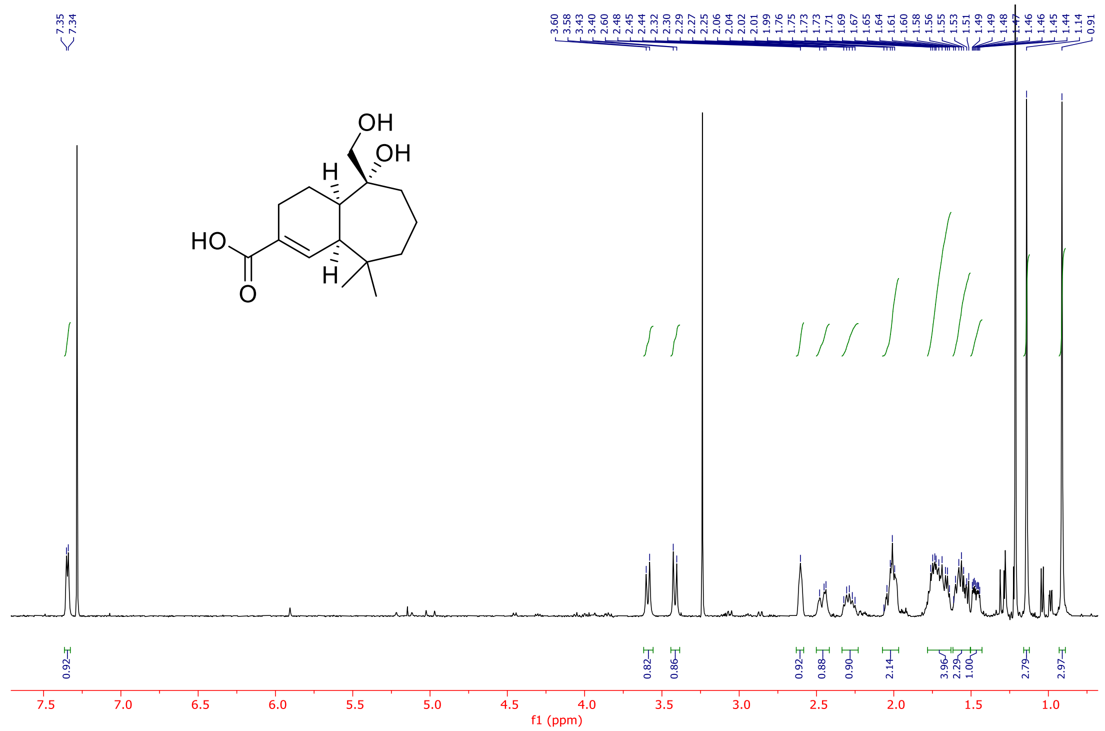


**Figure S38.** $^{1}H$ NMR spectrum (500 MHz, $CDCl_3$) of compound **8**.

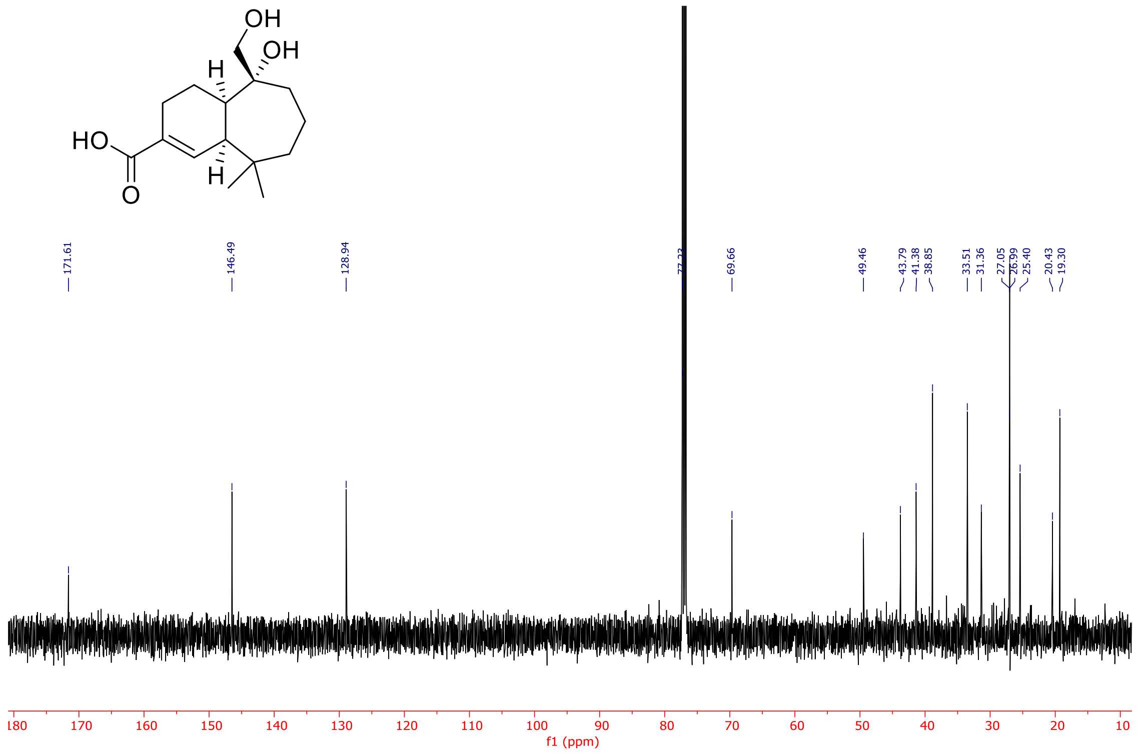


**Figure S39.** $^{13}C$ NMR spectrum (126 MHz, $CDCl_3$) of compound **8**.

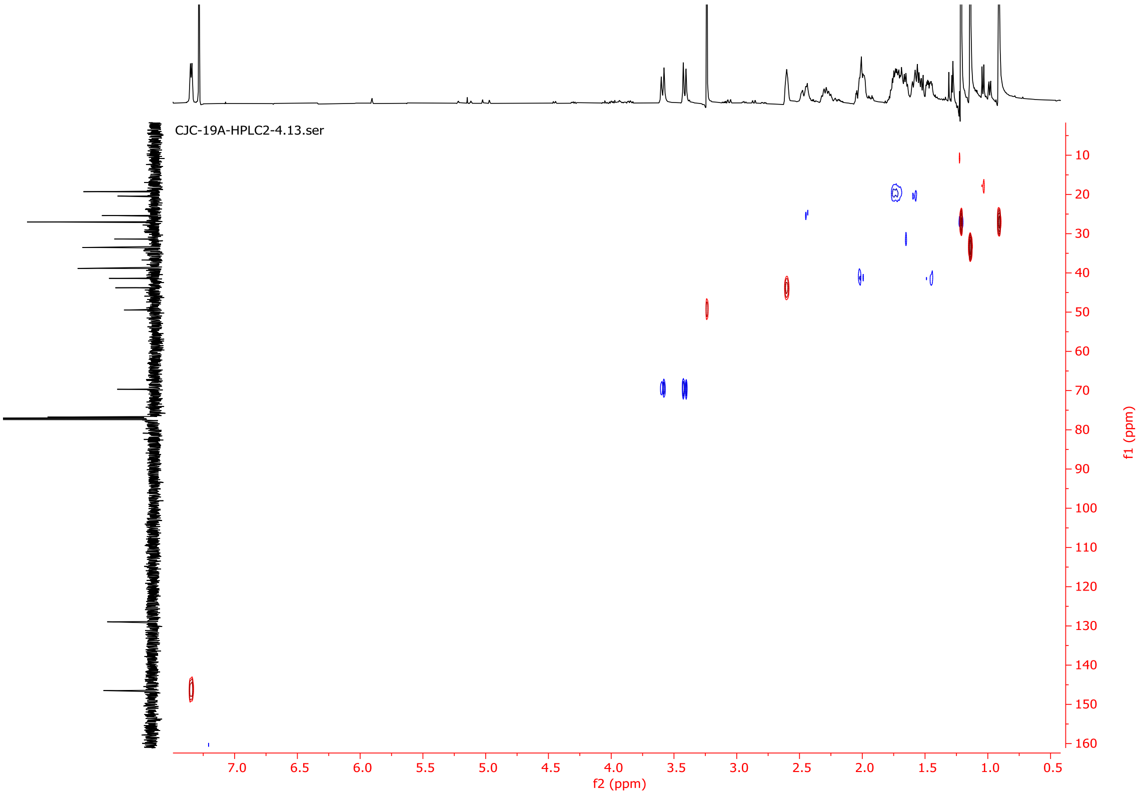


**Figure S40.** HSQC spectrum (500/126 MHz, $CDCl_3$) of compound **8**.

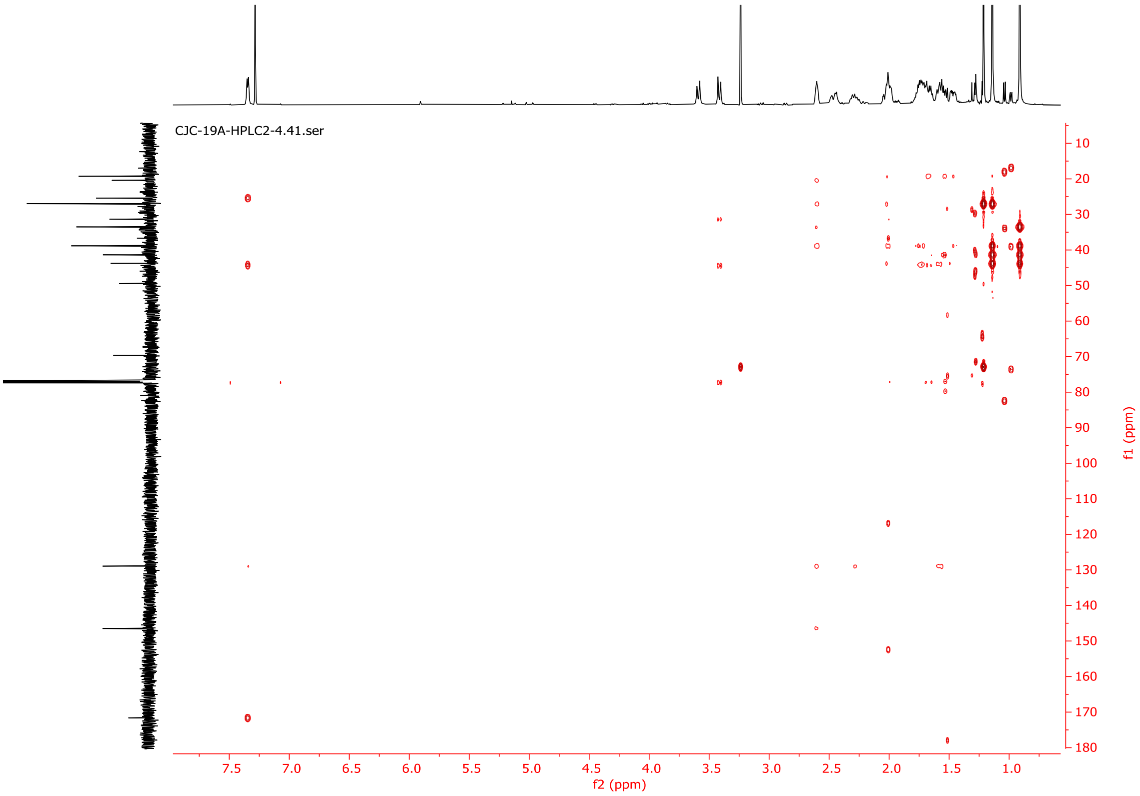


**Figure S41.** HMBC spectrum (500/126 MHz, $CDCl_3$) of compound **8**.

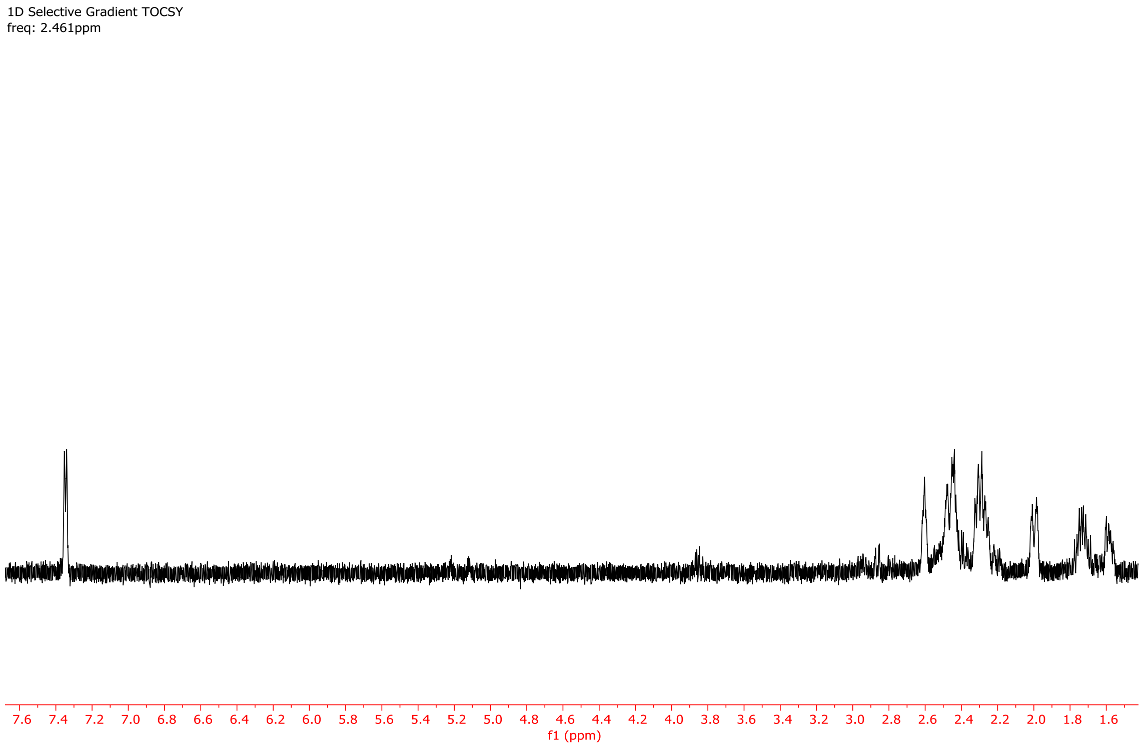


**Figure S42.** 1D TOCSY spectrum (500 MHz, $CDCl_3$) of compound **8**.

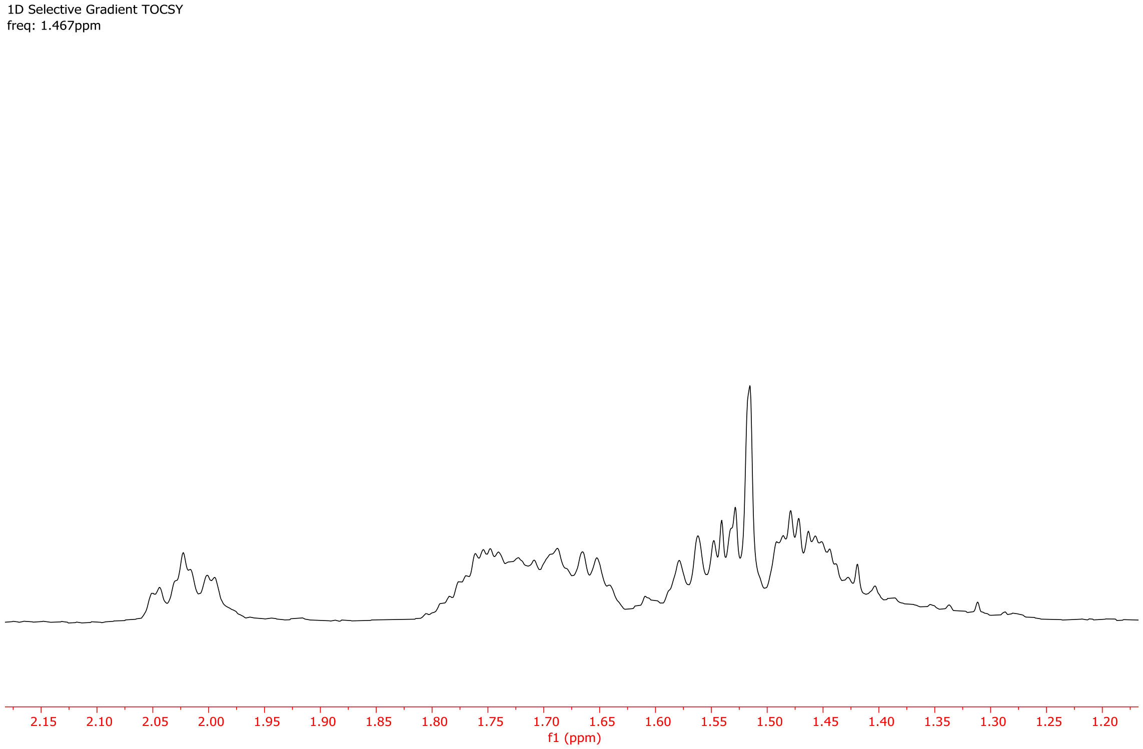


**Figure S43.** 1D TOCSY spectrum (500 MHz, $CDCl_3$) of compound **8**.

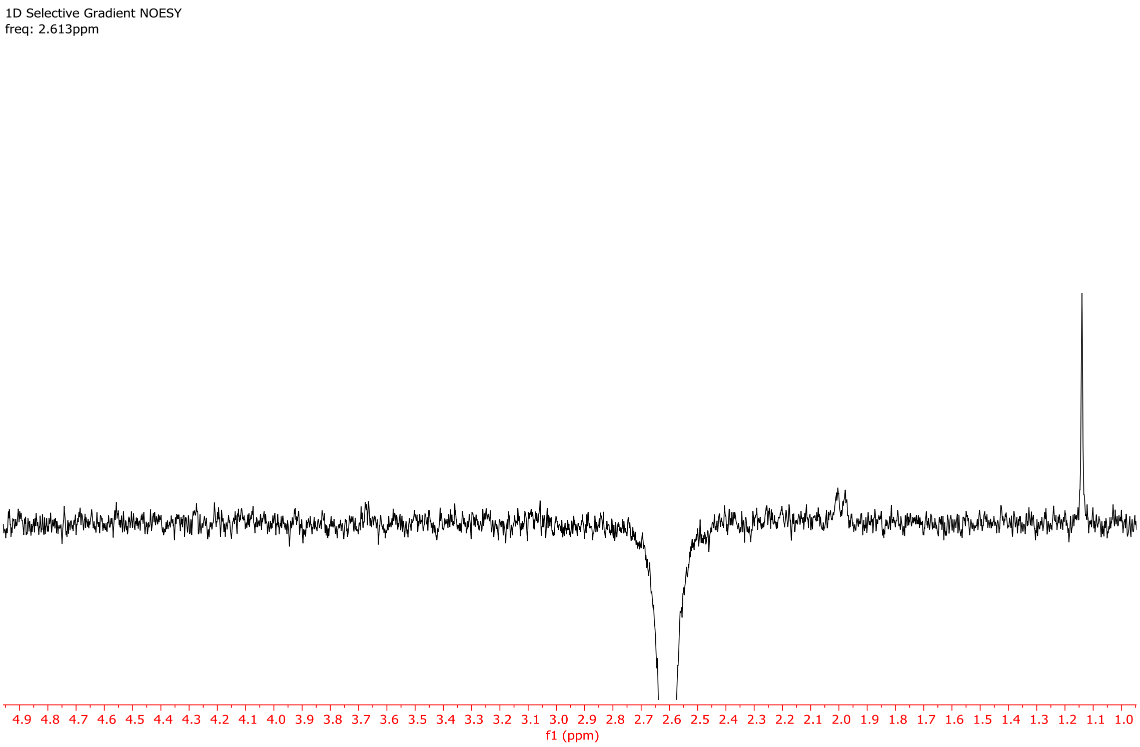


**Figure S44.** 1D NOE spectrum (500 MHz, $CDCl_3$) of compound **8**.

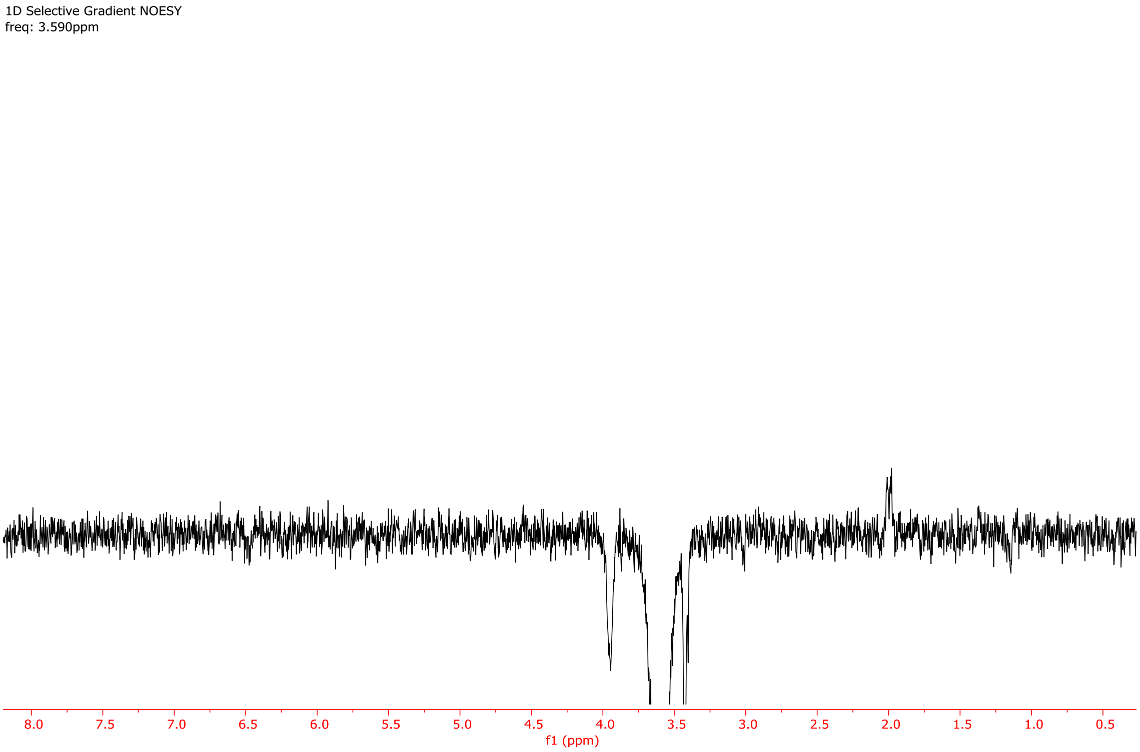


**Figure S45.** 1D NOE spectrum (500 MHz, $CDCl_3$) of compound **8**.

**Table S1.** Cartesian coordinates (mol2) of the conformer with the lowest energy out of all possible conformers of **5**.

| | | X AXIS | Y AXIS | Z AXIS |
|---|---|---|---|---|
| 1 | C1 | 0.419938024 | 1.568234917 | -0.480247428 |
| 2 | C2 | -0.574710954 | 0.987502469 | 0.563361060 |
| 3 | C3 | 0.316732738 | 3.077603256 | -0.228113223 |
| 4 | C4 | 0.306597634 | 3.178391452 | 1.300750139 |
| 5 | C5 | -0.418109710 | 1.922374368 | 1.759976777 |
| 6 | C6 | -0.434573027 | -0.505788569 | 0.888720037 |
| 7 | C7 | -0.314848619 | -1.422926924 | -0.336086168 |
| 8 | C8 | 0.964163117 | -1.233856091 | -1.169692239 |
| 9 | C9 | 0.873679676 | -0.195024947 | -2.288254644 |
| 10 | C10 | 0.162194429 | 1.076531651 | -1.886993492 |
| 11 | C11 | -0.666458344 | 1.684364810 | -2.735786233 |
| 12 | C12 | -0.820573520 | 1.690941471 | 3.006554637 |
| 13 | C13 | -0.267954487 | -2.925248922 | -0.006404005 |
| 14 | C14 | 0.512056405 | -3.495309794 | -1.181886579 |
| 15 | O15 | 1.221861340 | -2.507135548 | -1.780153863 |
| 16 | O16 | 0.570956311 | -4.636170939 | -1.548737047 |
| 17 | C17 | 0.467934275 | -3.305956001 | 1.287732366 |
| 18 | H18 | 1.435095240 | 1.263455590 | -0.181822191 |
| 19 | H19 | -1.578909647 | 1.133524597 | 0.129540316 |
| 20 | O20 | -1.520449269 | -0.944017393 | 1.696011639 |
| 21 | H21 | 1.128915480 | 3.634876584 | -0.702432055 |
| 22 | H22 | -0.628626835 | 3.462410443 | -0.627209498 |
| 23 | H23 | 1.335153502 | 3.171468622 | 1.684346339 |
| 24 | H24 | -0.167459313 | 4.092804695 | 1.669410102 |
| 25 | H25 | 0.450856144 | -0.636963074 | 1.520267112 |
| 26 | H26 | -1.176609685 | -1.236708940 | -0.994552234 |
| 27 | H27 | 1.812260010 | -1.017737667 | -0.506759793 |
| 28 | H28 | 0.356339357 | -0.663795490 | -3.131694715 |
| 29 | H29 | 1.890238342 | 0.038953034 | -2.630031147 |

| | | | | |
|---|---|---|---|---|
| 30 | H30 | -1.171156128 | 2.614964560 | -2.496229272 |
| 31 | H31 | -0.861893190 | 1.271419576 | -3.721522494 |
| 32 | H32 | -1.317754901 | 0.768466716 | 3.283224080 |
| 33 | H33 | -0.653766265 | 2.428656303 | 3.788014457 |
| 34 | H34 | -1.262585326 | -3.374776751 | 0.011379908 |
| 35 | H35 | 1.480258334 | -2.886811177 | 1.318098785 |
| 36 | H36 | -0.087118734 | -2.946248936 | 2.156686112 |
| 37 | H37 | 0.555653235 | -4.394130565 | 1.347619604 |
| 38 | H38 | -2.337325640 | -0.738337386 | 1.222914850 |

**Table S2.** Comparation between the theorical $^{13}C$ RMN of the lower energy conformer of **5** and the corresponding experimental values.

| **Carbon** | **Type** | **Theorical $^{13}C$ RMN (δ ppm)** | **Experimental $^{13}C$ RMN (δ ppm)** | **Difference** |
|---|---|---|---|---|
| C1 | CH | 45.7 | 45.9 | -0.3 |
| C2 | $CH_2$ | 30.6 | 30.1 | 0.5 |
| C3 | $CH_2$ | 33.9 | 34.2 | -0.3 |
| C4 | =C | 152.6 | 152.5 | 0.1 |
| C5 | CH | 53.4 | 54.9 | -1.5 |
| C6 | CH | 72.1 | 71.2 | 0.9 |
| C7 | CH | 55.4 | 54.8 | 0.6 |
| C8 | CH | 74.8 | 76.7 | **-1.8** |
| C9 | $CH_2$ | 40 | 39.6 | **0.4** |
| C10 | =C | 143.9 | 144.1 | -0.2 |
| C11 | CH | 38.9 | 39.6 | -0.7 |
| C12 | =C | 177.3 | 179.1 | -1.9 |
| C13 | $CH_3$ | 11 | 10.5 | 0.5 |
| C14 | $=CH_2$ | 113 | 110.7 | 2.3 |
| C15 | $=CH_2$ | 111.5 | 109.6 | 1.9 |
| RMSD | | | | 1.18 |
| Max. abs. | | 2.29 | | |

**Max abs.** expresses the largest deviations between the calculated and experimental chemical shifts; **RMSD** is a statistical parameter: $\delta_{13C}$ root-mean-square deviation.

**Optical Rotation calculation of 5.**

%nprocshared=28

%mem=1800MB

%chk=5.chk

# opt freq wb97xd/6-311+g(2d,p) geom=connectivity int=(ultrafine,acc2e=12)

Compound 5

0 1

| | | | |
|---|---|---|---|
| C | 1.69621400 | 0.94224300 | -0.37545800 |
| C | 1.52600400 | -0.41724300 | 0.35292600 |
| C | 3.21704500 | 1.10530700 | -0.40532100 |
| C | 3.71258500 | -0.28966900 | -0.78532800 |
| C | 2.70434400 | -1.23644500 | -0.16011300 |
| C | 0.16460400 | -1.10868500 | 0.23801700 |
| C | -1.04884300 | -0.20191300 | 0.45353100 |
| C | -1.21836400 | 0.91000500 | -0.58937100 |
| C | -0.51754100 | 2.22485300 | -0.27135000 |
| C | 0.89148600 | 2.05980300 | 0.24083600 |
| C | 1.35899200 | 2.85535000 | 1.19542000 |
| C | 2.84147400 | -2.55334100 | -0.10931300 |
| C | -2.40492600 | -0.92291100 | 0.38235600 |
| C | -3.34081700 | 0.18124400 | -0.07474800 |
| O | -2.62825900 | 1.17348500 | -0.65259700 |
| O | -4.53193100 | 0.21416500 | -0.00320400 |
| C | -2.50080500 | -2.07612000 | -0.62377800 |
| H | 1.36781200 | 0.80925500 | -1.41522300 |
| H | 1.69196500 | -0.20175200 | 1.41986600 |
| O | 0.09006200 | -2.20953000 | 1.13582800 |
| H | 3.53674600 | 1.88853000 | -1.09348600 |
| H | 3.58551400 | 1.36701800 | 0.59000700 |
| H | 3.69827800 | -0.41047400 | -1.87340900 |
| H | 4.73167100 | -0.49089100 | -0.45222700 |

| | | | |
|---|---|---|---|
| H | 0.09652000 | -1.55798400 | -0.75707000 |
| H | -0.95840600 | 0.27036100 | 1.44053600 |
| H | -0.91954900 | 0.54178400 | -1.57699200 |
| H | -1.12223900 | 2.75622000 | 0.46650200 |
| H | -0.50733100 | 2.84134900 | -1.17637000 |
| H | 2.37671600 | 2.79094500 | 1.55980600 |
| H | 0.73025100 | 3.61706700 | 1.64280500 |
| H | 2.08663300 | -3.19022300 | 0.33239700 |
| H | 3.72800800 | -3.02686900 | -0.51868700 |
| H | -2.73209300 | -1.27880600 | 1.35791300 |
| H | -2.18670100 | -1.76717400 | -1.62450100 |
| H | -1.87747500 | -2.91053100 | -0.30505100 |
| H | -3.53546100 | -2.41432200 | -0.68925700 |
| H | 0.28144700 | -1.89332600 | 2.02239700 |

--Link1--

%nprocshared=28

%mem=1600MB

%chk= 5

# wb97xd/6-311+g(2d,p) scrf=(iefpcm,solvent=dichloromethane) Polar=OptRot CPHF=RdFreq Geom=Check Guess=Read

Compound_5

0 1

589nm

| | 1 | 2 | 3 |
|---|---|---|---|
| 1 | -0.121576D+02 | -0.299568D+02 | -0.975750D+01 |
| 2 | -0.465279D+01 | 0.153813D+02 | 0.587177D+02 |
| 3 | 0.443992D+02 | -0.107397D+03 | -0.145360D+01 |

w= 0.077357 a.u., Optical Rotation Beta= -0.5900 au.

Molar Mass = 248.3212 grams/mole, [Alpha] ( 5890.0 A) = -91.93 deg.

Dipole-magnetic dipole polarizability for W= 0.078829:

**Table S3.** Lower energy conformations of **6**.

| Label | Energy (kcal/mol) | Boltzmann Weights | RMSD | Max. Absolute | DP4 (%) |
|---|---|---|---|---|---|
| M01 | -462776.40 | 0.271 | 2.32 | 5.08 | 1.2 |
| M02 | -462775.74 | 0.089 | 1.74 | 3.23 | 30.5 |
| M03 | -462775.94 | 0.125 | 2.14 | 5.24 | 10.0 |
| M04 | -462774.94 | 0.023 | 1.78 | 3.20 | 33.0 |
| M05 | -462775.80 | 0.098 | 2.18 | 4.26 | 0.2 |
| M06 | -462775.12 | 0.031 | 1.97 | 3.47 | 2.5 |
| M07 | -462775.76 | 0.092 | 2.07 | 4.29 | 7.5 |
| M08 | -462775.89 | 0.113 | 2.91 | 6.56 | 0.0 |
| M09 | -462775.03 | 0.027 | 2.26 | 4.21 | 0.1 |
| M10 | -462775.35 | 0.046 | 2.26 | 4.94 | 0.2 |
| M11 | -462775.15 | 0.033 | 2.71 | 6.15 | 0.0 |
| M12 | -462775.24 | 0.038 | 2.35 | 5.29 | 0.5 |
| M13 | -462774.23 | 0.007 | 2.62 | 5.83 | 0.0 |
| M14 | -462774.12 | 0.006 | 1.98 | 3.92 | 14.4 |

**RMSD**: $\delta_{13C}$ root-mean-square deviation. **Max Absolute**: *expresses the largest deviations between the calculated and experimental chemical shifts*. **DP4 (%)**: Score for predicting the correct conformer based on theoretical NMR data according to Goodman's procedure.

**Table S4.** Lower energy conformations of **6** epimer at C-7.

| Label | Energy (kcal/mol) | Boltzmann Weights | RMSD | Max. Absolute | DP4 (%) |
|---|---|---|---|---|---|
| M01 | -462777.08 | 0.118 | 2.49 | 5.55 | 0.0 |
| M02 | -462777.37 | 0.192 | 4.71 | 7.9 | 0.0 |
| M03 | -462777.83 | 0.420 | 2.21 | 5.33 | 0.2 |
| M04 | -462776.72 | 0.064 | 1.95 | 4.48 | 1.1 |
| M05 | -462776.21 | 0.027 | 5.21 | 8.04 | 0.0 |
| M06 | -462776.55 | 0.048 | 5.45 | 8.37 | 0.0 |
| M07 | -462775.97 | 0.018 | 3.79 | 9.74 | 0.0 |
| M08 | -462776.29 | 0.031 | 3.88 | 8.33 | 0.0 |
| M09 | -462775.86 | 0.015 | 5.44 | 9.79 | 0.0 |
| M10 | -462775.65 | 0.011 | 4.43 | 7.52 | 0.0 |
| M11 | -462775.61 | 0.010 | 3.12 | 6.22 | 0.0 |
| M12 | -462775.75 | 0.013 | 4.45 | 8.56 | 0.0 |
| M13 | -462775.88 | 0.016 | 2.20 | 4.61 | 1.9 |
| M14 | -462775.52 | 0.008 | 1.66 | 4.18 | 96 |
| M15 | -462775.48 | 0.008 | 2.22 | 4.97 | 0.9 |

**RMSD**: $\delta^{13}C$ root-mean-square deviation. **Max Absolute**: *expresses the largest deviations between the calculated and experimental chemical shifts*. **DP4 (%)**: Score for predicting the correct conformer based on theoretical NMR data according to Goodman's procedure.

**Table S5.** Cartesian coordinates (mol2) of the global minimum conformer (M01) of **6**.

| | | X AXIS | Y AXIS | Z AXIS |
|---|---|---|---|---|
| 1 | C1 | -0.257215006 | -0.751955662 | 0.584154770 |
| 2 | C2 | -1.637028060 | -0.701361602 | 1.203784856 |
| 3 | C3 | -2.683521333 | -0.017862474 | 0.733725684 |
| 4 | C4 | -2.603552221 | 0.804633843 | -0.526365679 |
| 5 | C5 | -1.174622654 | 0.990257145 | -1.036885398 |
| 6 | C6 | -0.378674703 | -0.318665739 | -0.900901254 |
| 7 | C7 | 0.908288848 | -0.356759893 | -1.750372745 |
| 8 | C8 | 1.955223721 | 0.689966858 | -1.369703167 |
| 9 | C9 | 2.836377627 | 0.389507993 | -0.147952845 |
| 10 | C10 | 2.248848491 | -0.433813147 | 1.005741763 |
| 11 | C11 | 0.830113631 | -0.103972657 | 1.518385879 |
| 12 | C12 | 0.656119282 | 1.405908563 | 1.734508056 |
| 13 | C13 | 0.704939897 | -0.775547513 | 2.903476661 |
| 14 | C14 | 0.566498190 | -0.195758743 | -3.252217059 |
| 15 | C15 | -4.031069052 | -0.059718886 | 1.401254240 |
| 16 | H16 | 0.028041504 | -1.814457937 | 0.562100011 |
| 17 | H17 | -1.782987776 | -1.300849837 | 2.099910993 |
| 18 | H18 | -3.213347634 | 0.309865267 | -1.298302164 |
| 19 | H19 | -3.075240862 | 1.782891708 | -0.362536149 |
| 20 | H20 | -1.211206890 | 1.321453460 | -2.079057132 |
| 21 | H21 | -0.689242598 | 1.790234583 | -0.473219380 |
| 22 | H22 | -1.026523099 | -1.094725812 | -1.347365437 |
| 23 | H23 | 2.608579264 | 0.831791295 | -2.236907834 |
| 24 | H24 | 1.424567805 | 1.639502272 | -1.238825516 |
| 25 | H25 | 3.728761052 | -0.146975261 | -0.487453918 |
| 26 | H26 | 3.197337475 | 1.350224720 | 0.240062452 |
| 27 | H27 | 2.269625322 | -1.493125890 | 0.729515736 |
| 28 | H28 | 2.944335492 | -0.323943555 | 1.848463896 |
| 29 | H29 | -0.370432749 | 1.642859913 | 2.034707849 |
| 30 | H30 | 0.895442244 | 1.993693423 | 0.845432191 |

| | | | | |
|---|---|---|---|---|
| 31 | H31 | 1.327548202 | 1.745153995 | 2.532355582 |
| 32 | H32 | 1.533905203 | -0.463061527 | 3.547721533 |
| 33 | H33 | 0.740759773 | -1.869248921 | 2.822905354 |
| 34 | H34 | -0.223186091 | -0.499121892 | 3.411987087 |
| 35 | H35 | -0.309637114 | -0.827088306 | -3.489254826 |
| 36 | H36 | 0.305964107 | 0.838757088 | -3.492696112 |
| 37 | H37 | -4.343003462 | 0.943997814 | 1.717245508 |
| 38 | H38 | -4.029953007 | -0.709318278 | 2.281505295 |
| 39 | H39 | -4.799791464 | -0.424774648 | 0.707586564 |
| 40 | O40 | 1.538859981 | -1.645437817 | -1.639858168 |
| 41 | O41 | 1.668645509 | -0.539082236 | -4.051911032 |
| 42 | H42 | 0.842812996 | -2.314720199 | -1.692671241 |
| 43 | H43 | 2.078640158 | -1.289351503 | -3.592074901 |

**Table S6.** Cartesian coordinates (mol2) of the conformer M02 of **6.**

| | | X AXIS | Y AXIS | Z AXIS |
|---|---|---|---|---|
| 1 | C1 | -0.754654791 | 0.190197404 | 0.573040479 |
| 2 | C2 | -0.709822623 | 1.576154736 | 1.179347815 |
| 3 | C3 | -0.021764558 | 2.618796791 | 0.707313160 |
| 4 | C4 | 0.817115521 | 2.525038360 | -0.541569000 |
| 5 | C5 | 1.014577622 | 1.087345713 | -1.024979047 |
| 6 | C6 | -0.297932966 | 0.294193807 | -0.905250103 |
| 7 | C7 | -0.350189440 | -1.015922054 | -1.734184292 |
| 8 | C8 | 0.716747449 | -2.034297531 | -1.331581430 |
| 9 | C9 | 0.401043609 | -2.909140102 | -0.106958495 |
| 10 | C10 | -0.441042260 | -2.311017485 | 1.028140246 |
| 11 | C11 | -0.121787244 | -0.886003089 | 1.531031073 |
| 12 | C12 | 1.383646172 | -0.709168137 | 1.775855874 |
| 13 | C13 | -0.819517857 | -0.748572930 | 2.902101887 |
| 14 | C14 | -0.196511260 | -0.719767246 | -3.240451676 |
| 15 | C15 | -0.072335179 | 3.974213743 | 1.358732074 |
| 16 | H16 | -1.813990839 | -0.096267740 | 0.526416327 |
| 17 | H17 | -1.319696118 | 1.732283445 | 2.066852874 |
| 18 | H18 | 0.329165464 | 3.121979894 | -1.328984040 |
| 19 | H19 | 1.791045594 | 3.004653988 | -0.373061445 |
| 20 | H20 | 1.377591436 | 1.107093110 | -2.059019593 |
| 21 | H21 | 1.803621256 | 0.615587296 | -0.434079313 |
| 22 | H22 | -1.075678804 | 0.932627830 | -1.356177139 |
| 23 | H23 | 0.901304601 | -2.707246954 | -2.179331090 |
| 24 | H24 | 1.655745105 | -1.489796382 | -1.179731114 |
| 25 | H25 | -0.133675299 | -3.800939568 | -0.451160078 |
| 26 | H26 | 1.356408761 | -3.267703507 | 0.296895540 |
| 27 | H27 | -1.494977499 | -2.335276292 | 0.733523768 |
| 28 | H28 | -0.341964328 | -2.998380221 | 1.878991844 |
| 29 | H29 | 1.615226637 | 0.321703001 | 2.066366181 |
| 30 | H30 | 1.990968637 | -0.960677839 | 0.902596204 |

| | | | | |
|---|---|---|---|---|
| 31 | H31 | 1.706754421 | -1.370157718 | 2.589580668 |
| 32 | H32 | -0.519877233 | -1.571774173 | 3.559765115 |
| 33 | H33 | -1.911078687 | -0.784272215 | 2.800348112 |
| 34 | H34 | -0.552241210 | 0.183880428 | 3.407999844 |
| 35 | H35 | 0.787918755 | -0.302281660 | -3.486075271 |
| 36 | H36 | -0.299580826 | -1.669636338 | -3.772202190 |
| 37 | H37 | 0.927262383 | 4.290739022 | 1.683454177 |
| 38 | H38 | -0.732741655 | 3.982170533 | 2.231038670 |
| 39 | H39 | -0.430168032 | 4.735553440 | 0.652866010 |
| 40 | O40 | -1.631331516 | -1.619257590 | -1.589623823 |
| 41 | O41 | -1.247648779 | 0.100359628 | -3.726660653 |
| 42 | H42 | -2.222573092 | -1.099581996 | -2.155340990 |
| 43 | H43 | -1.063361328 | 1.012566598 | -3.475837161 |

**Table S7.** Cartesian coordinates (mol2) of the conformer M04 of **6**.

| | | X AXIS | Y AXIS | Z AXIS |
|---|---|---|---|---|
| 1 | C1 | -0.772158083 | 0.206369495 | 0.563640212 |
| 2 | C2 | -0.744251132 | 1.589443072 | 1.178168555 |
| 3 | C3 | -0.034762988 | 2.630553437 | 0.735694815 |
| 4 | C4 | 0.844143944 | 2.539519221 | -0.485190156 |
| 5 | C5 | 1.057625945 | 1.103553307 | -0.965753011 |
| 6 | C6 | -0.261810196 | 0.316976505 | -0.896614953 |
| 7 | C7 | -0.284144524 | -0.992521060 | -1.727914736 |
| 8 | C8 | 0.763162272 | -2.016322544 | -1.287224827 |
| 9 | C9 | 0.395854319 | -2.894543427 | -0.080110743 |
| 10 | C10 | -0.484030824 | -2.296849756 | 1.025840306 |
| 11 | C11 | -0.179100580 | -0.873970446 | 1.542491782 |
| 12 | C12 | 1.316986066 | -0.702494325 | 1.842907727 |
| 13 | C13 | -0.925446911 | -0.738377975 | 2.887982780 |
| 14 | C14 | -0.064833148 | -0.689446297 | -3.218384362 |
| 15 | C15 | -0.103256819 | 3.983188932 | 1.391373732 |
| 16 | H16 | -1.830779312 | -0.075585412 | 0.480123978 |
| 17 | H17 | -1.385028042 | 1.744008779 | 2.044069619 |
| 18 | H18 | 0.379555612 | 3.132779297 | -1.287905673 |
| 19 | H19 | 1.811553663 | 3.019876744 | -0.283673793 |
| 20 | H20 | 1.447444767 | 1.130854665 | -1.988451992 |
| 21 | H21 | 1.827197838 | 0.629605541 | -0.350502609 |
| 22 | H22 | -1.007095850 | 0.958147346 | -1.388445813 |
| 23 | H23 | 0.981602117 | -2.687251540 | -2.128400338 |
| 24 | H24 | 1.694518130 | -1.472870334 | -1.094654632 |
| 25 | H25 | -0.129247508 | -3.782392093 | -0.448313157 |
| 26 | H26 | 1.333854272 | -3.259245376 | 0.357133504 |
| 27 | H27 | -1.526178054 | -2.316614069 | 0.693375911 |
| 28 | H28 | -0.417271052 | -2.986837911 | 1.877852032 |
| 29 | H29 | 1.540040925 | 0.327986962 | 2.140787604 |
| 30 | H30 | 1.955308589 | -0.954704238 | 0.992402289 |

| | | | | |
|---|---|---|---|---|
| 31 | H31 | 1.608160662 | -1.363888290 | 2.668441536 |
| 32 | H32 | -0.652764807 | -1.564718929 | 3.553566072 |
| 33 | H33 | -2.012832919 | -0.769187940 | 2.746517199 |
| 34 | H34 | -0.673394137 | 0.191634763 | 3.405947631 |
| 35 | H35 | 0.939166440 | -0.288696688 | -3.408514701 |
| 36 | H36 | -0.162747993 | -1.640706895 | -3.758586462 |
| 37 | H37 | 0.886341700 | 4.296937033 | 1.748160631 |
| 38 | H38 | -0.790442501 | 3.988943242 | 2.242762511 |
| 39 | H39 | -0.437466133 | 4.747601985 | 0.677446696 |
| 40 | O40 | -1.569181289 | -1.601961293 | -1.637564333 |
| 41 | O41 | -1.072010211 | 0.222489770 | -3.627827738 |
| 42 | H42 | -2.160564382 | -1.025070188 | -2.142332542 |
| 43 | H43 | -1.101717868 | 0.233786931 | -4.590320556 |

**Table S8.** Cartesian coordinates (mol2) of the global minimum conformer M01 of **6** epimer at C-7.

| | | X AXIS | Y AXIS | Z AXIS |
|---|---|---|---|---|
| 1 | C1 | -0.689314321 | 0.182719260 | 0.609211190 |
| 2 | C2 | -0.802063243 | 1.545843422 | 1.262670756 |
| 3 | C3 | -0.248182267 | 2.684566471 | 0.839451353 |
| 4 | C4 | 0.613120316 | 2.741162161 | -0.394141432 |
| 5 | C5 | 1.025814651 | 1.358163857 | -0.886310380 |
| 6 | C6 | -0.163793483 | 0.384055020 | -0.843273776 |
| 7 | C7 | 0.086325765 | -0.856284119 | -1.739475775 |
| 8 | C8 | -0.744339697 | -2.099935016 | -1.367013214 |
| 9 | C9 | -0.214401117 | -2.884809537 | -0.168663461 |
| 10 | C10 | -0.537086702 | -2.299981087 | 1.203858461 |
| 11 | C11 | -0.018618789 | -0.885961706 | 1.549392789 |
| 12 | C12 | 1.514140804 | -0.847507066 | 1.518236100 |
| 13 | C13 | -0.457483222 | -0.633504975 | 3.006182542 |
| 14 | C14 | -0.272937832 | -0.458983611 | -3.183954369 |
| 15 | C15 | -0.479413773 | 3.995055565 | 1.541612802 |
| 16 | H16 | -1.729431829 | -0.166379444 | 0.511596268 |
| 17 | H17 | -1.439570413 | 1.592163759 | 2.143157297 |
| 18 | H18 | 0.056078275 | 3.273663383 | -1.181749477 |
| 19 | H19 | 1.503188656 | 3.354527061 | -0.197591629 |
| 20 | H20 | 1.424236871 | 1.431239335 | -1.902715433 |
| 21 | H21 | 1.843370928 | 0.974115338 | -0.274949283 |
| 22 | H22 | -1.001657704 | 0.910075173 | -1.330765824 |
| 23 | H23 | -1.789980848 | -1.802500750 | -1.204078272 |
| 24 | H24 | -0.743666024 | -2.759799435 | -2.243025133 |
| 25 | H25 | -0.641898853 | -3.894638452 | -0.203258733 |
| 26 | H26 | 0.867524583 | -3.003433973 | -0.286307767 |
| 27 | H27 | -1.630103925 | -2.298898056 | 1.334773856 |
| 28 | H28 | -0.144152807 | -2.991860078 | 1.961402224 |
| 29 | H29 | 1.880890510 | 0.139828142 | 1.822358856 |

| | | | | |
|---|---|---|---|---|
| 30 | H30 | 1.914765733 | -1.078161060 | 0.528249824 |
| 31 | H31 | 1.916303997 | -1.582129037 | 2.227716344 |
| 32 | H32 | -0.104450957 | -1.450388331 | 3.645790962 |
| 33 | H33 | -1.550144877 | -0.589178537 | 3.099776090 |
| 34 | H34 | -0.041968510 | 0.298529618 | 3.400751367 |
| 35 | H35 | -1.364163785 | -0.366661994 | -3.282249797 |
| 36 | H36 | 0.183315575 | 0.510281304 | -3.424551180 |
| 37 | H37 | 0.466369206 | 4.413370727 | 1.910136977 |
| 38 | H38 | -1.159737472 | 3.888718796 | 2.391992837 |
| 39 | H39 | -0.905026654 | 4.737548475 | 0.853573235 |
| 40 | O40 | 1.471787992 | -1.171680084 | -1.728300364 |
| 41 | O41 | 0.231395802 | -1.473524521 | -4.040693557 |
| 42 | H42 | 1.622270466 | -1.684224881 | -2.536948577 |
| 43 | H43 | 0.252688971 | -1.135201115 | -4.941874697 |

**Table S9.** Cartesian coordinates (mol2) of the conformer M14 of conformer M01 of **6** epimer at C-7.

| | | X AXIS | Y AXIS | Z AXIS |
|---|---|---|---|---|
| 1 | C1 | -0.192048915 | -0.596684857 | 0.659161620 |
| 2 | C2 | -1.486643648 | -0.532803479 | 1.443109308 |
| 3 | C3 | -2.642294669 | -0.026816826 | 1.005921433 |
| 4 | C4 | -2.794864869 | 0.539991293 | -0.381926964 |
| 5 | C5 | -1.462368316 | 0.723097746 | -1.108864587 |
| 6 | C6 | -0.538976520 | -0.478574071 | -0.849120516 |
| 7 | C7 | 0.620656758 | -0.590215109 | -1.867262643 |
| 8 | C8 | 1.630037507 | 0.558470025 | -1.797685465 |
| 9 | C9 | 2.685728460 | 0.520540516 | -0.682483340 |
| 10 | C10 | 2.305238682 | 0.003717410 | 0.716713635 |
| 11 | C11 | 0.914696497 | 0.320160963 | 1.305433899 |
| 12 | C12 | 0.621453707 | 1.825207349 | 1.228294719 |
| 13 | C13 | 0.997156285 | -0.044679491 | 2.804676165 |
| 14 | C14 | 1.298343214 | -1.966930864 | -1.811041162 |
| 15 | C15 | -3.888058351 | -0.033063015 | 1.849490707 |
| 16 | H16 | 0.196754986 | -1.616435824 | 0.805745133 |
| 17 | H17 | -1.464209034 | -0.961307844 | 2.442690037 |
| 18 | H18 | -3.441309015 | -0.139389165 | -0.958468244 |
| 19 | H19 | -3.336651733 | 1.494387860 | -0.336212557 |
| 20 | H20 | -1.660924995 | 0.819140165 | -2.180367993 |
| 21 | H21 | -0.997274918 | 1.655807942 | -0.780664129 |
| 22 | H22 | -1.156374679 | -1.364827481 | -1.058726685 |
| 23 | H23 | 2.170081333 | 0.568318392 | -2.750288413 |
| 24 | H24 | 1.065012569 | 1.498756305 | -1.754687172 |
| 25 | H25 | 3.520497536 | -0.097766850 | -1.033118792 |
| 26 | H26 | 3.084923432 | 1.537612331 | -0.586333524 |
| 27 | H27 | 2.439782295 | -1.084378830 | 0.760350721 |
| 28 | H28 | 3.058262152 | 0.406176757 | 1.406570230 |
| 29 | H29 | -0.393352988 | 2.044253488 | 1.577787397 |

| | | | | |
|---|---|---|---|---|
| 30 | H30 | 0.730531132 | 2.229411113 | 0.219309709 |
| 31 | H31 | 1.324186737 | 2.370699758 | 1.869788530 |
| 32 | H32 | 1.116456617 | -1.125652669 | 2.949315130 |
| 33 | H33 | 0.106852527 | 0.277314634 | 3.351887864 |
| 34 | H34 | 1.859058819 | 0.449869754 | 3.265666729 |
| 35 | H35 | 1.819721805 | -2.123173417 | -0.866875076 |
| 36 | H36 | 0.512240058 | -2.734846496 | -1.893801140 |
| 37 | H37 | -4.699916081 | -0.572092688 | 1.343908367 |
| 38 | H38 | -4.248521269 | 0.989084548 | 2.022176031 |
| 39 | H39 | -3.719862907 | -0.504376769 | 2.822256089 |
| 40 | O40 | 0.047474949 | -0.604282333 | -3.190976988 |
| 41 | O41 | 2.255640397 | -2.104142078 | -2.834156114 |
| 42 | H42 | -0.046231520 | 0.308038085 | -3.487629533 |
| 43 | H43 | 1.789095973 | -1.837616280 | -3.639562417 |
